# Electroceramics for High-Energy Density Capacitors: Current Status and Future Perspectives

**DOI:** 10.1021/acs.chemrev.0c01264

**Published:** 2021-04-28

**Authors:** Ge Wang, Zhilun Lu, Yong Li, Linhao Li, Hongfen Ji, Antonio Feteira, Di Zhou, Dawei Wang, Shujun Zhang, Ian M Reaney

**Affiliations:** † Department of Materials Science and Engineering, University of Sheffield, Sheffield S1 3JD, U.K.; & The Henry Royce Institute, Sir Robert Hadfield Building, Sheffield S1 3JD, U.K.; § Inner Mongolia Key Laboratory of Ferroelectric-related New Energy Materials and Devices, School of Materials and Metallurgy, Inner Mongolia University of Science and Technology, Baotou 014010, China; ∥ Laboratory of Thin Film Techniques and Optical Test, Xi’an Technological University, Xi’an 710032, China; ⊥ Christian Doppler Laboratory for Advanced Ferroic Oxides, Sheffield Hallam University, Sheffield S1 1WB, U.K.; # Electronic Materials Research Lab, Key Lab of Education Ministry/International Center for Dielectric Research, School of Electronic and Information Engineering, Xi’an Jiaotong University, Xi’an 710049, China; ∇ Shenzhen Institute of Advanced Electronic Materials, Shenzhen Institute of Advanced Technology, Chinese Academy of Sciences, Shenzhen 518055, China; @ Institute for Superconducting and Electronic Materials, Australian Institute for Innovative Materials, University of Wollongong, Wollongong, NSW 2500, Australia

## Abstract

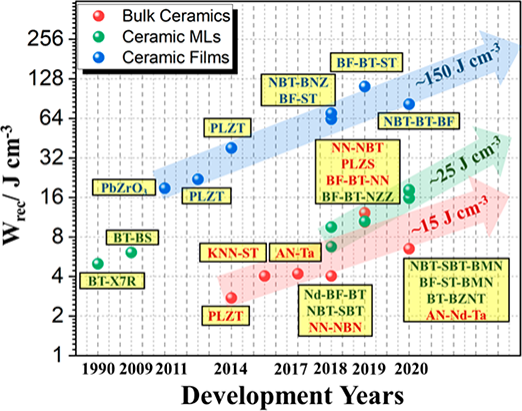

Materials exhibiting high energy/power density are currently needed to meet the growing demand of portable electronics, electric vehicles and large-scale energy storage devices. The highest energy densities are achieved for fuel cells, batteries, and supercapacitors, but conventional dielectric capacitors are receiving increased attention for pulsed power applications due to their high power density and their fast charge–discharge speed. The key to high energy density in dielectric capacitors is a large maximum but small remanent (zero in the case of linear dielectrics) polarization and a high electric breakdown strength. Polymer dielectric capacitors offer high power/energy density for applications at room temperature, but above 100 °C they are unreliable and suffer from dielectric breakdown. For high-temperature applications, therefore, dielectric ceramics are the only feasible alternative. Lead-based ceramics such as La-doped lead zirconate titanate exhibit good energy storage properties, but their toxicity raises concern over their use in consumer applications, where capacitors are exclusively lead free. Lead-free compositions with superior power density are thus required. In this paper, we introduce the fundamental principles of energy storage in dielectrics. We discuss key factors to improve energy storage properties such as the control of local structure, phase assemblage, dielectric layer thickness, microstructure, conductivity, and electrical homogeneity through the choice of base systems, dopants, and alloying additions, followed by a comprehensive review of the state-of-the-art. Finally, we comment on the future requirements for new materials in high power/energy density capacitor applications.

## 1 Introduction

To limit global warming to <1.50 °C, as set out in the Paris agreement, carbon dioxide emissions need to decrease ∼45% by 2030 and reach net-zero by 2050.^1,2^ Technologies based on renewable resources such as sun, wind, and tides will play a pivotal role to meet these targets. Although the increasing deployment of renewable energies is encouraging, there still are many barriers to the replacement of power generation from traditionally high CO_2_-emitting sectors based on coal and gas, which is still a critical and large portion of the energy generation, due to the intermittent nature of renewables. Hence, to simultaneously move away from fossil fuels and to circumvent the unpredictability inherent in clean energy resources, it is necessary to integrate energy-harvesting technologies with energy storage devices.

Energy storage, therefore, is emerging as a key enabler for sustainable renewable technologies, particularly for the electrification of transportation but also in more specialized applications such as heart defibrillators and active armor.^3^ Technologies already exist to store energy, such as batteries, electrochemical supercapacitors, and electrostatic capacitors.^4−16^ The latter are electrical energy-storage devices belonging to the category of passive components, which are ubiquitous in electronics. Indeed, every year more than 3 trillion multilayer ceramic capacitors (MLCCs) are manufactured from BaTiO_3_ (BT), the prototypical ferroelectric (FE) ceramic.^17−22^

**Figure 1:**
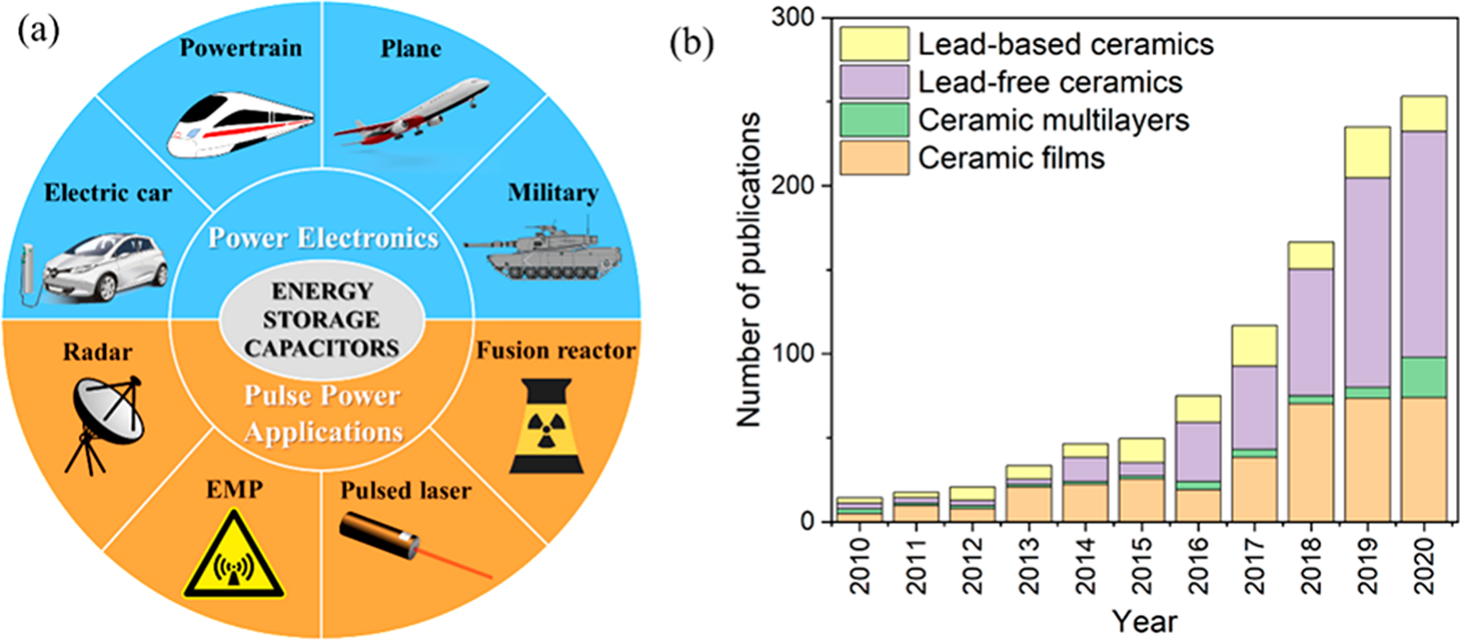
(a) Applications for energy storage capacitors. *EMP: electromagnetic pulse. (b) Number of annual publications on lead-based ceramics, lead-free ceramics, ceramic multilayers, and ceramic films for energy storage capacitors from 2010 to 2020. (Collected from Web of Science, search “energy storage/density lead-based ceramic, lead-free ceramic, multilayer ceramic, ceramic capacitor, ceramic films but NOT polymer”). Reproduced with permission from PixaBay, Creative Commons License.

In comparison with Li-ion batteries or fuel cells, the nonpolarized electrostatic or dielectric capacitors possess high power density (∼10^4^–10^5^ W/kg) resulting from their faster charging/discharging characteristics (∼μs), which are advantageous for power electronics in electrical vehicles (EVs) and pulse power applications (Figure 1a).^4,23−27^ Hence, electrostatic capacitors are emerging as promising candidates for energy storage devices, where high power density in combination with high energy density are important technological requirements, as illustrated by the exponential rise in publications devoted to energy storage involving electrostatic ceramic capacitors, Figure 1b. Apart from high energy density and fast charging–discharging rate, other properties such as temperature/frequency stability, fatigue resistance, lifetime reliability, equivalent series resistance, and manufacturing cost are equally important for dielectric capacitors used in practical applications. New electroceramics are, therefore, required to facilitate near-engine power electronics, exhibit ultrafast charging, and have more durable EV performance at high temperature and voltage. Thus, future electroceramics must (i) deliver high energy density (*W*_rec_ > 10 J cm^–3^) and conversion efficiencies (η > 90%); (ii) endure wider temperature ranges (−50–250 °C) and frequency ranges (1–1000 Hz); (iii) exhibit greater reliability (>10^5^ cycles) and fatigue resistance (<5% change over capacitor lifetime); and (iv) be compatible with cost-effective internal electrodes and be easily integrated with other components.

Historically, many different dielectric materials, ranging from paper and plastic to ceramics, have been employed in the fabrication of electrostatic capacitors. Nowadays, capacitors are fabricated from either polymers or ceramics because they offer the best combination of properties in terms of capacitance, dielectric loss, breakdown strength (BDS), and for the latter, thermal stability.

The prospects of employing ceramic capacitors for energy storage can be traced back to the 1960s work by Jaffe^28^ from the Clevite Corp., USA. One decade later, Burn and Smyth^29^ from Sprague Electric Company evaluated the energy storage performance in SrTiO_3_ (ST) and BT with applied electric fields up to 400 kV cm^–1^. Until that point, quantitative data of energy storage on these materials were limited to fields generally smaller than 150 kV cm^–1^ due to the relatively low dielectric BDS of the fabricated ceramics. They emphasized that the maximum energy density for a ceramic should be obtained for thinner dielectric layers due to the lower probability for the occurrence of defects (such as pores, voids, or microcracks), which are well-known sources of dielectric breakdown. Later in 1990, Love,^30^ also from Sprague Electric Company, revisited energy storage in ceramic capacitors and highlighted empirical design principles to achieve enhanced energy storage in capacitors, as shown in Table 1. Commercial *C0G*-type capacitors are manufactured from low relative permittivity (*ε*_r_) linear dielectrics but may achieve an energy storage of 1 J cm^–3^, by virtue of their intrinsically high BDS. The significance of the BDS, to achieve high energy storage becomes apparent in the case of *X7R*-type capacitors, fabricated from high *ε*_r_ BT. An important correlation between dielectric BDS and the thickness (*t*) can be extracted from Table 1. Indeed, by halving the *t* of the dielectric layers, the energy storage appears to increase >3 fold. This effect has been recently captured by Yang and co-workers,^31^ who compiled BDS data from literature for several dielectric materials of different *t* and observed decay inversely proportional to (*t*)^*a*^, where *a* was determined as 0.5. Finally, when comparing the energy storage of *Z5U* and *X7R*, it becomes apparent that high *ε*_r_ alone is not a sufficient parameter to achieve high energy storage. Interestingly, Love^30^ stressed that the capacitor industry was rather conservative in terms of perfecting the BDS of ceramics to reach values near those of single-crystals, which would significantly enhance the energy storage in ceramic capacitors.

**Table 1:** General Characteristics of Commercial Type Ceramic Materials Relevant for Energy Storage (Adapted from Love^30^) Using Electronic Industries Alliance (EIA) Classifications^a^

dielectric type	dielectric BDS (V μm^–1^)	relative permittivity, *ε*_r_	*t* (μm)	energy at 1 kV (J cm^–3^)
*C0G* (temperature coefficient 0 with tolerance ±30 × 10^–6^/K)	65	75	18.5	0.88
*Z5U* (+10/+85 °C, Δ*C*/*C*_0_ = +22/–56%)	13.2	7500	95	0.02
*X7T* (−55/+125 °C, Δ*C*/*C*_0_ = +22/–33%)	16	2800	70	0.71
*X7R* (−55/+125 °C, Δ*C*/*C*_0_ = ± 15%)	30	2000	38	1.40
*X7R*	40	2000	30	1.34
*X7R*	90	1800	20	4.82

a Class I ceramic capacitors are accurate, temperature-compensating capacitors, *C0G* will have 0 drift with a tolerance of ±30 × 10^–6^/K. Class II ceramic capacitors have a dielectric with a high permittivity. *C* and *C*_0_ are represented capacitance value and capacitance value at 25 °C.

Love^30^ proposed that maximum energy storage density can be achieved in intermediate rather than high ε_r_ materials since they exhibit larger BDS. Fletcher and co-workers^32^ convincingly postulated that greater energy storage densities can indeed be achieved in FE materials, whose Curie temperature (*T*_c_) is adjusted to ensure that the material is operated in the paraelectric regime, where it shows a relatively small zero-field ε_r_, an approach already mentioned by Jaffe in 1961.^28^

In 2009, Ogihara and co-workers^33^ proposed the use of so-called weakly coupled relaxors, such as 0.7BaTiO_3_–0.3BiScO_3_ (0.7BT–0.3BS), to fabricate energy storage devices. This new conceptual approach aimed at exploiting the extraordinary temperature stability of *ε*_r_ exhibit by this family of materials. When compared with commercial *X7R* capacitors, 0.7BT–0.3BS capacitors displayed superior performance, reaching a recoverable energy density (*W*_rec_) of 6.1 J cm^–3^ at 730 kV cm^–1^. Again, the large dielectric BDS played a decisive role in this performance. More recently, in 2019 Wang, Reaney and co-workers^34^ unveiled a novel approach to enhance energy storage characteristics via the fabrication of chemically heterogeneous but electrically homogeneous ceramics, with *W*_rec_ reaching 10.5 J cm^–3^, as detailed later in this review.

Here, we present the principles of energy storage performance in ceramic capacitors, including an introduction to electrostatic capacitors, key parameters for evaluating energy storage properties, microstructural considerations, and critical electrical factors. Second, we will review the current state-of-the-art for lead and lead-free electroceramics for energy storage capacitors with bulk ceramics, ceramic multilayers (MLs), ceramic films and glass ceramics evaluated separately. Third, we will describe strategies for optimizing energy storage in electroceramics. Finally, we will demonstrate, with appropriate examples, a guide to the future development for electroceramics in energy storage capacitors.

## 2 Principles of Energy Storage in Electroceramics

### 2.1 Electrostatic Capacitors

**Figure 2:**
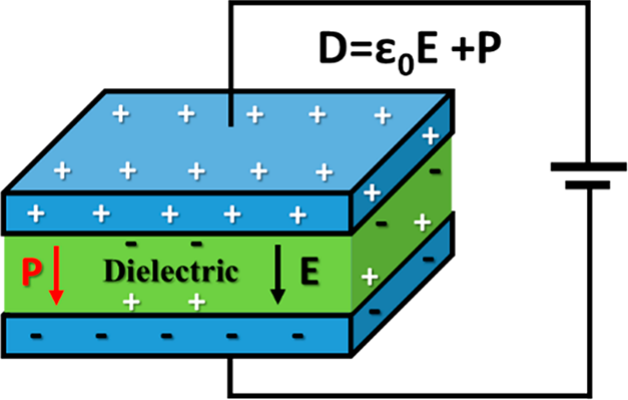
Schematic representation of an electrostatic capacitor, where *D, P*, and ε_0_ are electric displacement, polarization, and electric permittivity of free space (electric constant), respectively.

The simplest dielectric capacitor consists of two parallel metallic plates separated by an insulator, which becomes polarized under the application of an electric field. This is the defining behavior of a dielectric material. The actual capacitance, *C* (i.e., ability to store charge), of an ideal capacitor is given by the ratio of the charge, *Q*, stored on each metallic plate and the applied voltage, *V*, as shown by eq 1.
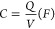
1Nevertheless, from a practical viewpoint, a more useful equation to compute the *C* of a real device, as illustrated in Figure 2, encompassing a dielectric material between two parallel plates of area, *A*, separated by a distance, *d*, subject to a *V*, can be obtained through the application of Gauss’s law
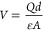
2where ε is the permittivity of the dielectric, and a measure of its polarizability. Combination of eqs 1 and 2 provides the relationship:
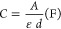
3From eq 3, it becomes immediately apparent that the ability of dielectric capacitor to charge and, therefore store energy, is ultimately associated with ε of the dielectric.

### 2.2 Key Parameters for Evaluating Energy Storage Properties

During the application of a *V*, the electrostatic energy stored, *W*, in the dielectric can be estimated by
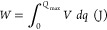
4where *Q*_max_ is the maximum charge achieved at the end of the charging cycle and d*q* is the incremental charge increase during the charging cycle. The volumetric energy density, *W*_st_ (i.e., the energy stored per volume unit, *A d*), is a common key performance indicator, expressed by
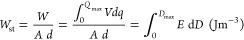
5where *E* is the electric field and *D*_max_ is the electric displacement in the material under the maximum applied field, *E*_max_. The electrical displacement (*D*) corresponds to the charge density (*Q*/*A*) on the metallic plates and is expressed by *D* = ε_0_*E + P* (Figure 2), where *P* is the polarization (surface charge density).

**Figure 3:**
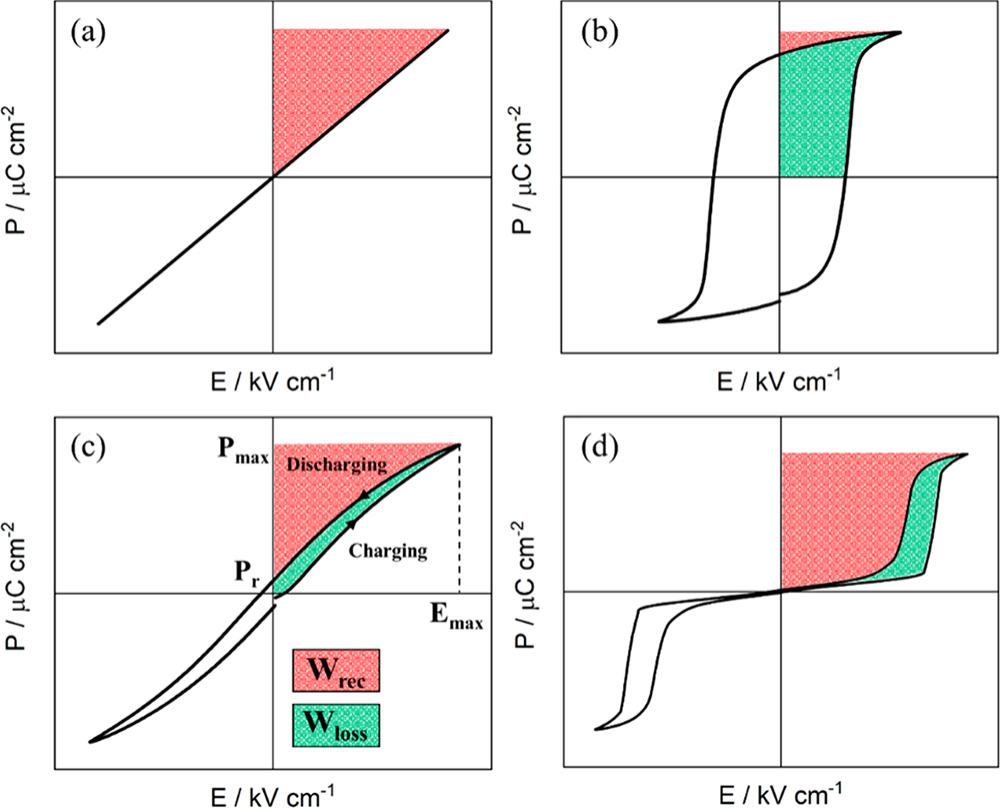
Four distinctive *P–E* hysteresis loops and their energy storage behavior: (a) linear, (b) FE, (c) relaxor-ferroelectric (with the schematic of energy storage calculation), and (d) antiferroelectric materials. *W_loss_ is loss energy density.

For high ε materials, *D* is approximately equal to *P*, and it follows that *D* = ε*E* = ε_0_*ε*_r_*E*, where *ε*_0_ is the permittivity of free space (= 8.854 × 10^–12^F m^–1^) and ε_r_ is the relative permittivity, which is the ε/*ε*_0_ ratio. This approximation allows stored energy density (*W*_st_) to be defined in terms of *P*, as follows
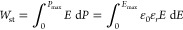
6where *P*_max_ is the maximum polarization reached at the *E*_max_. From a practical viewpoint, eq 6 is prevalent in the calculation of *W*_st_ because several experimental methods exist to determine *P* under an applied *E*. In 1961, Jaffe^28^ pointed out that the recoverable energy (*W*_rec_) corresponds to the area above the discharging curve, whose upper limit is given by the *P*_max_. Essentially, the mathematical integration of the area above a polarization-electric (*P–E*) loop provides an estimate of *W*_rec_, as schematically illustrated in Figure 3 for four distinct types of polarization response.

For linear dielectrics such as Al_2_O_3_, where *ε*_r_ is independent of the applied *E*. The calculation of *W*_rec_ from the *P–E* response illustrated in Figure. 3a, is given by
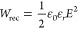
7which clearly shows that *W*_rec_ is dependent on *ε*_r_ and *E*. Parts b–d of Figure 3 show cases where polarization responses deviate from linearity, and consequently, the computation of *W*_rec_ needs to be carried out using eq 6. The response illustrated in Figure 3b is typical of a classical FE material, such as BT, where the hysteresis is linked to polarization switching of macroscopic FE domains, as explained in detail in the review by Damjanovic.^35^ Already in 1961, Jaffe^28^ stressed that in FEs, charging energy is mainly absorbed by domain switching and is retained as remanent polarization (*P*_r_). The typically high remanence of classical FEs can be effectively minimized via chemical doping, giving rise to the response shown in Figure 3c, which is characteristic of relaxor-ferroelectrics (RFEs), such as doped-BT and Pb(Mg_1/3_Nb_2/3_)O_3_.^36^

It is now generally accepted that relaxor behavior originates from the response of polar nanoregions (PNRs) to an alternating *E*. RFEs remain unsaturated at high applied *E*, and therefore, any increment of the *E* will have a contribution to energy storage. Remanence-free materials are therefore, preferable for achieving high *W*_rec_. Linear dielectric materials meet this requirement but due to their low *ε*_r_, energy storage is limited. Antiferroelectrics (AFEs) display low-remanence under low *E* but at large *E* the *P–E* loop opens due to the stabilization of an FE with respect to AFE phase and they display a saturated polarization, as illustrated in Figure 3d. In principle, therefore, as suggested by Jaffe,^28^ AFEs should afford advantages for high energy storage, providing that dielectric breakdown issues are eliminated (i.e., the BDS should be high enough to induce the AFE-FE phase transition).

From the above, it becomes evident that nonlinear dielectric materials such as FEs, RFEs, and AFEs exhibit energy dissipation (*W*_loss_); therefore, the *W*_rec_ is actually the most important parameter, as schematically illustrated in Figure 3c (red area). Hence, *W*_rec_ becomes
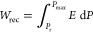
8Energy conversion efficiency of a capacitor can then be calculated as
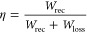
9where *W*_loss_ is the energy loss during discharging, which correlates to the area enclosed by the *P–E* loop (Figure 3c green area).

Electric-field induced polarization can be determined via the measurement of charge, current, and voltage responses, typically achieved using either the Sawyer–tower, the virtual ground, the shunt or the current step methods. Each presents advantages and disadvantages as listed in Table 2. For details of each method, the reader is referred to Prume and co-workers.^37^ Prume, Schmitz, and Tiedke proposed that overall the virtual ground method offers the highest precision for the measurement of FEs.

**Table 2:** Comparison of Different Hysteresis Measurement Methods for FEs^37^

method	measured quantity	reference component	integration necessary	bandwidth requirement	influence of parasitics
Sawyer–tower	charge Q	capacitor	no	moderate	high
virtual ground	current I	no	yes	high	low
shunt	current I	resistor	yes	high	high
current step	voltage V	no	no	moderate	moderate

### 2.3 Key Factors for Optimizing Energy Density

The microstructural features of electroceramics, such as density, grain size, secondary phases and core–shell structures, play an important role in energy storage properties. Simultaneously, the intrinsic electrical response, e.g., band gap, alongside the electrical microstructure, i.e., the distribution of conductive and resistive elements, are equally critical factors for the optimization of energy density. The following section reviews these factors, and gives examples of where and how they may be optimized.

#### 2.3.1 Intrinsic Band Gap

The band gap (*E*_g_) is the forbidden energy between the top of the valence band and bottom of the conduction band. *E*_g_ is commonly used to define insulator (*E*_g_ > 4.0 eV), semiconductor (0.0 eV < *E*_g_ < 4.0 eV), and metal (*E*_g_ = 0.0 eV). For semiconductor, the intrinsic BDS can be defined as
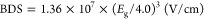
10where BDS is direct proportional to *E*_g_.^38^ Thus, semiconductors with wider *E*_g_ have higher intrinsic BDS. The electronic structure and band gaps of semiconductor can be studied theoretically using, e.g., linear discriminant analysis, or experimentally, e.g., absorbance spectroscopy and diffuse reflectance spectroscopy.^39^ A general rule of thumb is that the activation energy (*E*_a_) for conduction is approximately half *E*_*g*_. Both may be increased by doping or through the formation of solid solutions, often delivering higher BDS and *W*_rec_.^40,41^

**Figure 4:**
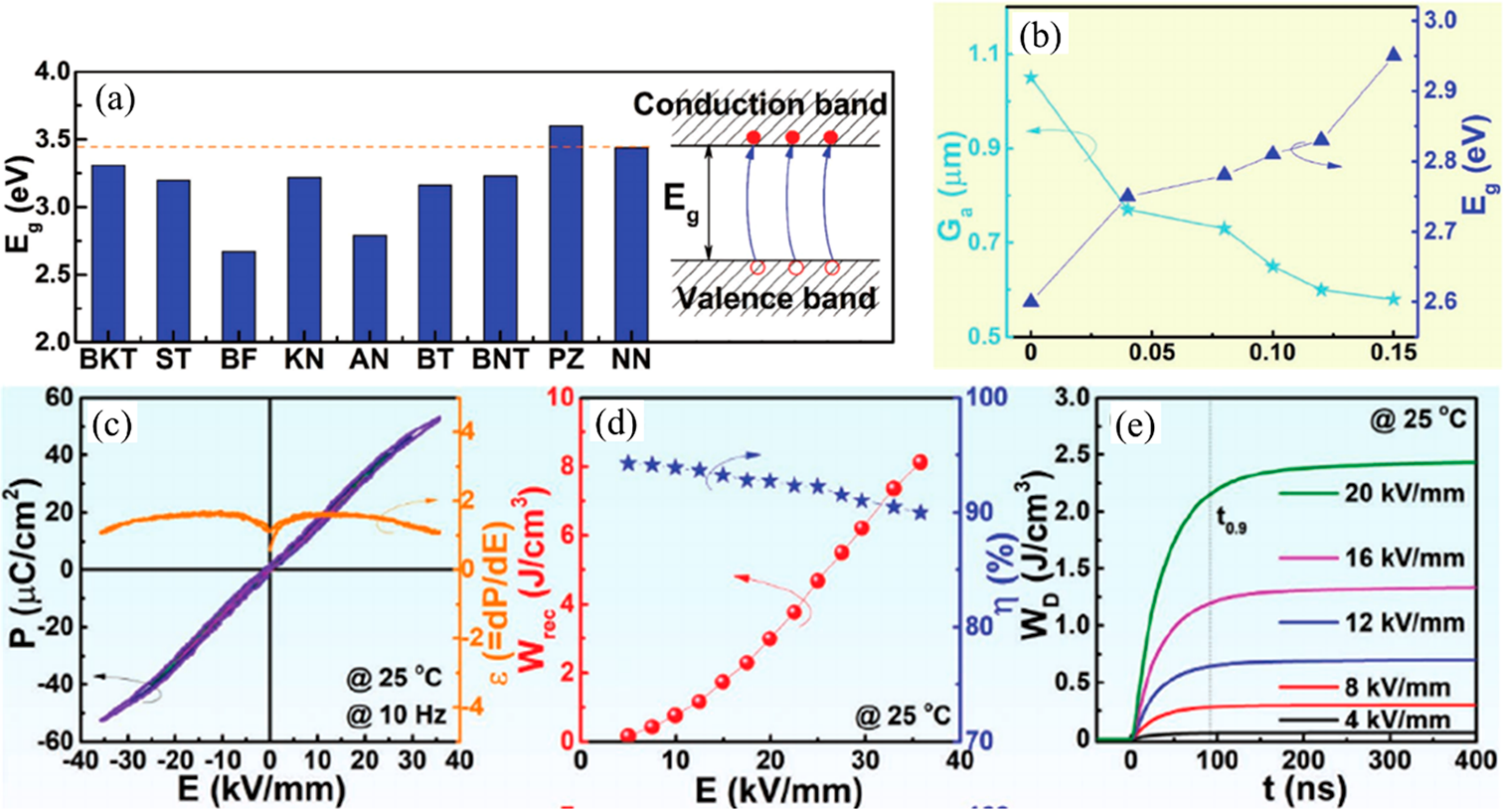
(a) Comparison of the *E*_g_ among dielectric perovskites and a schematic of electronic breakdown. (b) Variation of average grain size and *E*_g_ as a function of NN concentration. (c) *P–E* loops and d*P*/d*E* under different *E*, (d) *W*_rec_ and η values, and (e) pulsed overdamped discharging energy density (*W*_D_) of the BF–BT–0.10NN ceramic. (a) Reproduced with permission from ref (42). Copyright 2019 John Wiley and Sons. (b-e) Reproduced with permission from ref (43). 2020 John Wiley and Sons.

For example, the highest *E*_g_ ∼ 3.58 eV among all different kinds of lead-free electroceramics was found in NaNbO_3_ (NN), as shown in Figure 4a.^42^ Thus, NN was introduced into Na_0.5_Bi_0.5_TiO_3_ (NBT) and BiFeO_3_–BaTiO_3_ (BF–BT) to enhance *E*_g_. The *E*_g_ for BF–BT–*x*NN ceramics increased from 2.5 eV up to 2.95 eV for *x* ≤ 0.15, as shown in Figure 4b accompanied by significant enhanced *W*_rec_ ∼ 8.12 J cm^–3^ under electric field ∼400 kV cm^–1^, along with greater thermal stability (±10%, −50 to +250 °C) and ultrafast discharge rate (*t*_0.9_ < 100 ns), Figure 4.^43^

#### 2.3.2 Electrical Microstructure

The distribution of regions with different conductivity and *ε*_r_ are important aspects of the so-called “electrical microstructure” of electroceramics.^44^ In many instances, such as the core–shell microstructure or grain boundary response of BT based ceramics, the distributions are markedly heterogeneous and lead to localization of the electrical field strength in lower *ε*_r_ regions or pathways for breakdown through interconnected conducting regions. In 2019, electrical homogeneity was for the first-time proposed by Wang, Reaney and co-workers in the BF–BT system as a key factor to optimize BDS and as a consequence *W*_rec_. Electrical heterogeneity was effectively eliminated by alloying with a third end-member so that it became more difficult to form a conductive pathway at high field, resulting in higher BDS and *W*_rec_.^34^

A homogeneous electrical microstructure may be obtained in many different ways such as heat-treatment in the appropriate atmosphere (N_2_, Air, O_2_) provided the type and magnitude of electrical conductivity is affected by oxygen stoichiometry. Practically, however, in production, a suitable dopant strategy is utilized once the conduction type is known (*p* vs *n* type). For example, the conductivity of BF-ST-based compositions is suppressed by doping with 3 mol % Nb on the B-site to compensate for Bi volatilization and the formation of oxygen vacancies (*V*_O_^..^), through variation of the Fe valence (Fe^3+^ to Fe^4+^).^45^

For materials with more than one bulk-like region, e.g., phase mixtures, core–shell microstructures, or surface layers, alternating current (AC) impedance spectroscopy (IS) is able to show multiple responses and the resistance (*R*) and *C* can be extracted.^46−53^ Both the volume fraction and difference in magnitude of *R* and *C* for multiple electrical responses are equally important in influencing energy storage performance. Given the importance of the electrical microstructure, a brief outline of the role of IS is described and its advantages with respect to direct current methods are emphasized.

Direct current (DC) electrical measurements are the most commonly employed technique to evaluate the electrical characteristics of materials. However, they merely give the overall response instead of the properties of specific regions (e.g., grains and grain boundaries) unless microprobe techniques are employed.^54,55^ Such techniques are useful but the sample volume is small, which casts doubt on their ability to represent global behavior and they are difficult to implement experimentally.

An alternative and much more convenient technique is IS. In IS measurements, an AC signal with small voltage over a wide range of frequency, typically 10^–2^ to 10^7^ Hz, is applied on the sample.^44,56^ The small voltage prevents any permanent change to the sample as well as yielding a (near) linear relationship between input and output. The wide range of frequencies allows separation of the response of different electro-active regions according to their relaxation times. For energy storage capacitors, impedance is capable of: (i) establishing the contributions to the electrical microstructure (grains, grain boundaries, core–shell structure and electrode–sample interface) and determine their individual conductivity and ε_r_ which give an insight into the distribution of electrical components within the sample; (ii) verifying the origin of the dominant electrical behavior (i.e., grains, grain boundary or interfacial layer response);^57,58^ and (iii) determining the conduction mechanism and charge carrier type which helps further interpret the electrical response of the material.^47^

Impedance can be defined as a complex number which usually contains both resistive and reactive (capacitive and/or inductive) components:
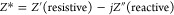
11Different electro-active regions of a material are characterized by a *R* and a *C*, usually in parallel. Then the electric relaxation time or time constant, τ, of each region can be expressed as its *R* and *C*
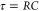
12at the frequency of maximum loss, *ω*_max_, it holds the relation:
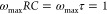
13Due to their different *R* and *C* values, electro-active regions can be separated in the frequency domain. Once the value of *R* and *C* are extracted, they can then be assigned to appropriate regions of the sample.

Normally the impedance measurement needs to be taken over a temperature and/or oxygen partial pressure (*p*O_2_) range to gain a better understanding of the conduction mechanism and the charge carrier. The associated activation energy, *E*_a_, can be estimated using Arrhenius equation
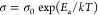
14where σ is the conductivity, *σ*_0_ is pre-exponential factor, *k* is the Boltzmann constant, and *T* is temperature. *E*_a_ may be related to predominant charge carrier and conduction mechanism. The type of charge carrier may also be determined to some extent by the *p*O_2_ dependence of conductivity, i.e., *p*-type: conductivity increases with increasing *p*O_2_; *n*-type: conductivity decreases with increasing *p*O_2_; ionic charge carrier: conductivity is independent with *p*O_2_.

#### 2.3.3 Density and Porosity

The density of the ceramic materials plays an essential role on electrical performance, especially BDS. Ceramics with higher density tend to support higher *E* closer to the intrinsic/theoretical BDS. In contrast, low density ceramics exhibit conductive pathway composed of pores/voids which result in short circuit under modest field strengths. The relationship between the voltage across the pore and the external *E* based on a “slab” model is shown below
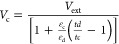
15where *V*_c_ and *V*_ext_ are the voltage applied cross the cavity pore and external applied voltage, *ε*_c_ and *ε*_d_ are the permittivity of the cavity and the dielectric, respectively,^59,60^ and *td* and *tc* are the thicknesses of the dielectric and cavity, respectively. Thus, the local *E* increases markedly for materials with larger pores and pore volumes, resulting in lower BDS.

**Figure 5:**
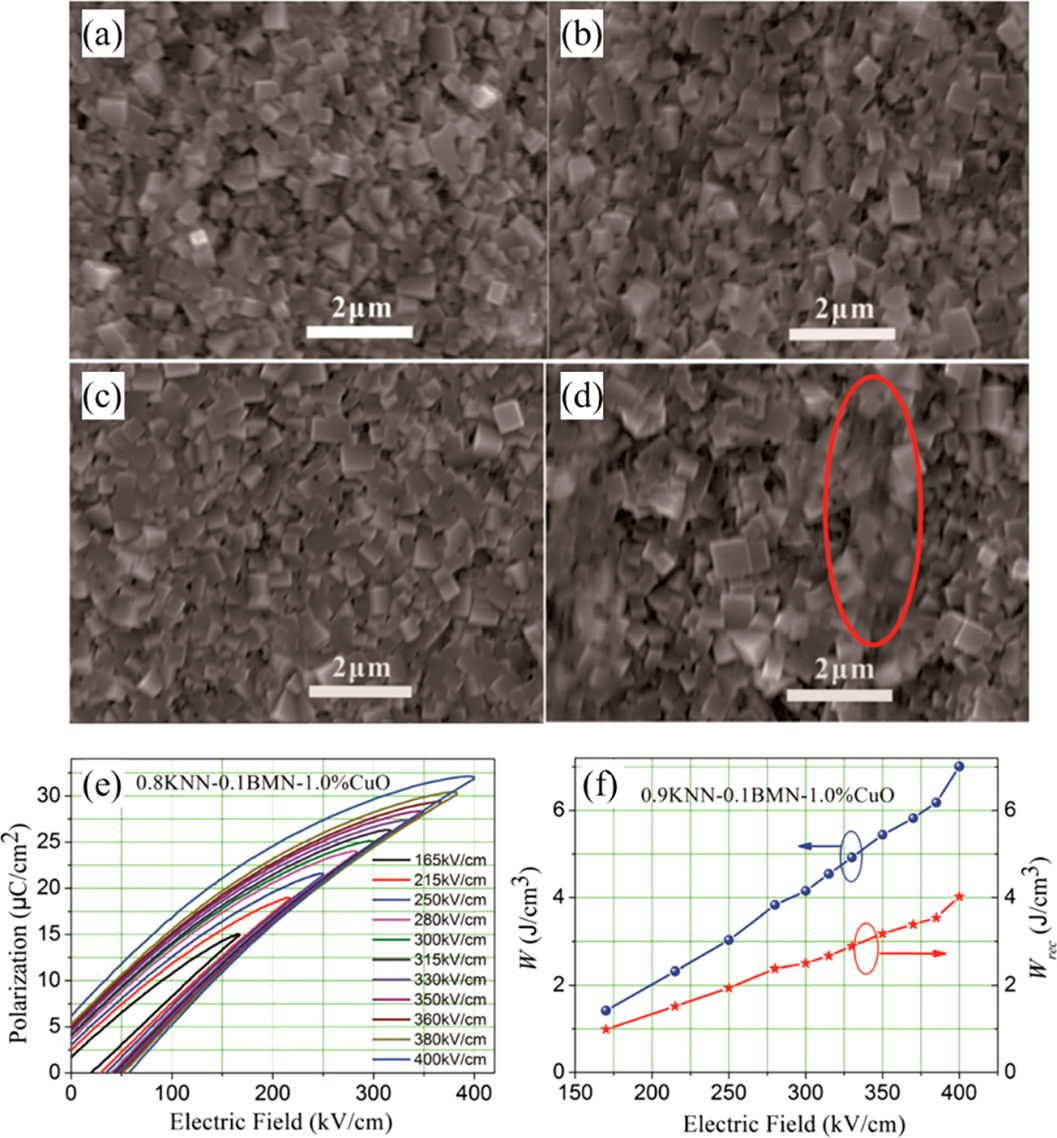
Scanning electron microscopy (SEM) images of the 0.9KNN-0.1BMN-*x* mol % CuO ceramics with (a) *x* = 0.25; (b) *x* = 0.5; (c) *x* = 1.0; (d) *x* = 1.5, as liquid phase is circled in red. (e) Unipolar *P–E* hysteresis loops and (f) Calculated *W* and *W*_rec_ under different *E* of 0.9KNN-0.1BMN-1 mol % CuO ceramics.^66^ Reproduced with permission from ref (66). Copyright 2017 John Wiley and Sons.

High density electroceramic materials are commonly obtained by optimization of the sintering conditions, including sintering temperature/time and heating/cooling rate. For ceramics that are difficult to densify using a conventional approach, sintering aids are often added.^61−64^ Higher density ceramics may be obtained by the addition of ZnO,^65^ CuO,^66^ and MgO,^62^ which enhances BDS and *W*_rec_. For K_0.5_Na_0.5_NbO_3_–Bi(Mg_2/3_Nb_1/3_)O_3_ (KNN–BMN), small amounts of CuO help densify ceramics through the formation of a transient liquid phase, as reported by Qu and co-workers (Figure 5).^64^ The sintering temperature was also reduced from 1150 to 930 °C, allowing compatibly with Cu or Ag/Pd internal electrode in MLs and giving rise to *W*_rec_ ∼ 4.02 J cm^–3^ at 400 kV cm^–1^ for 0.9KNN-0.1BMN with 1% mol CuO.^66^

Different sintering technologies, such as spark plasma sintering (SPS), two-step sintering,^67^ and the formation of coatings using chemical methods,^68−81^ have also been shown to improve density and give rise to higher BDS and *W*_rec_.

#### 2.3.4 Grain Size

**Figure 6:**
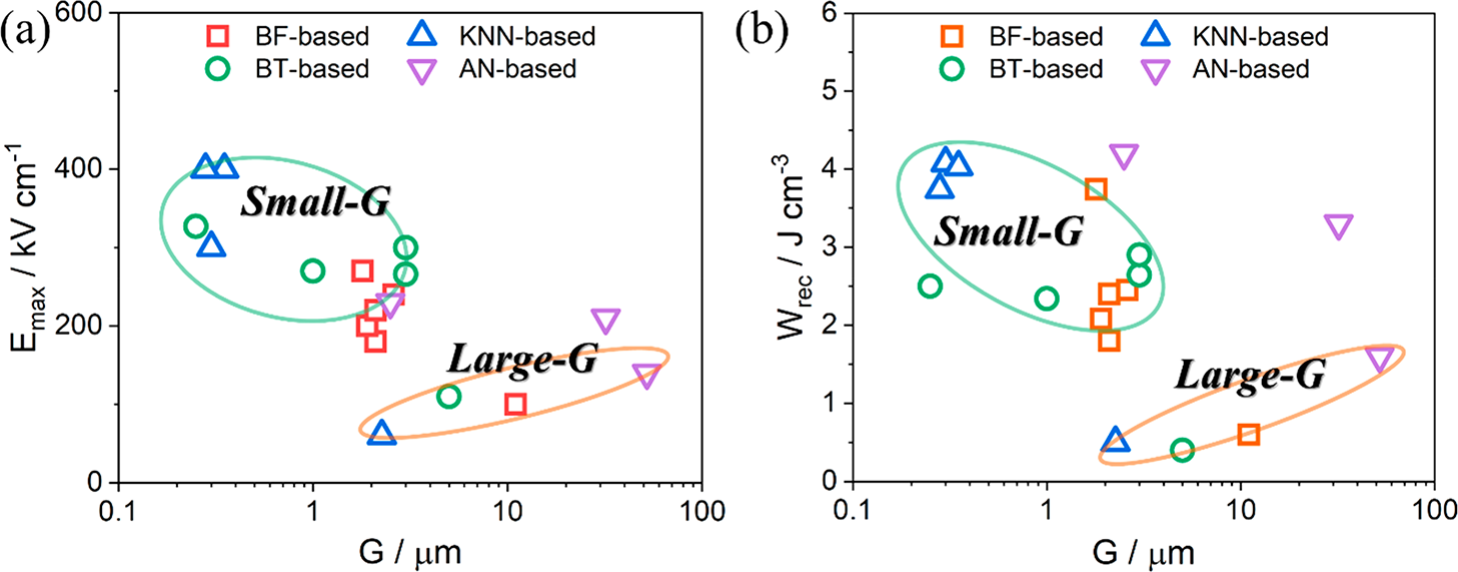
Relationship between energy storage properties of ceramics and *G*: (a) *G* vs *E*_max_ and (b) *G* vs *W*_rec_. *AN: AgNbO_3_.

The effect of grain size (*G*) on energy storage properties has been discussed for several electroceramics because of the relationship between BDS and *G*, expressed in eq 16
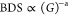
16where *a* is the exponent values being in the range of 0.2–0.4.^31,82−84^ Waser explained that leakage current in fine-*G* ceramics is lower than coarse-*G* ceramics due to the high grain boundary density which act as barriers for charge carriers.^85^ Thus, dielectric materials with high density and fine-*G* are required to optimize energy storage. *G* may be tailored by chemical doping and the formation of solid solution. It may also be modified by the application of an ultrathin coating on the primary particles prior to sintering via chemical coating methods, e.g., SiO_2_ on BT ceramics.^67,77,86−89^ The optimization on *E*_max_ and *W*_rec_ via grain size-engineering for several materials is illustrated in Figure 6.

**Figure 7:**
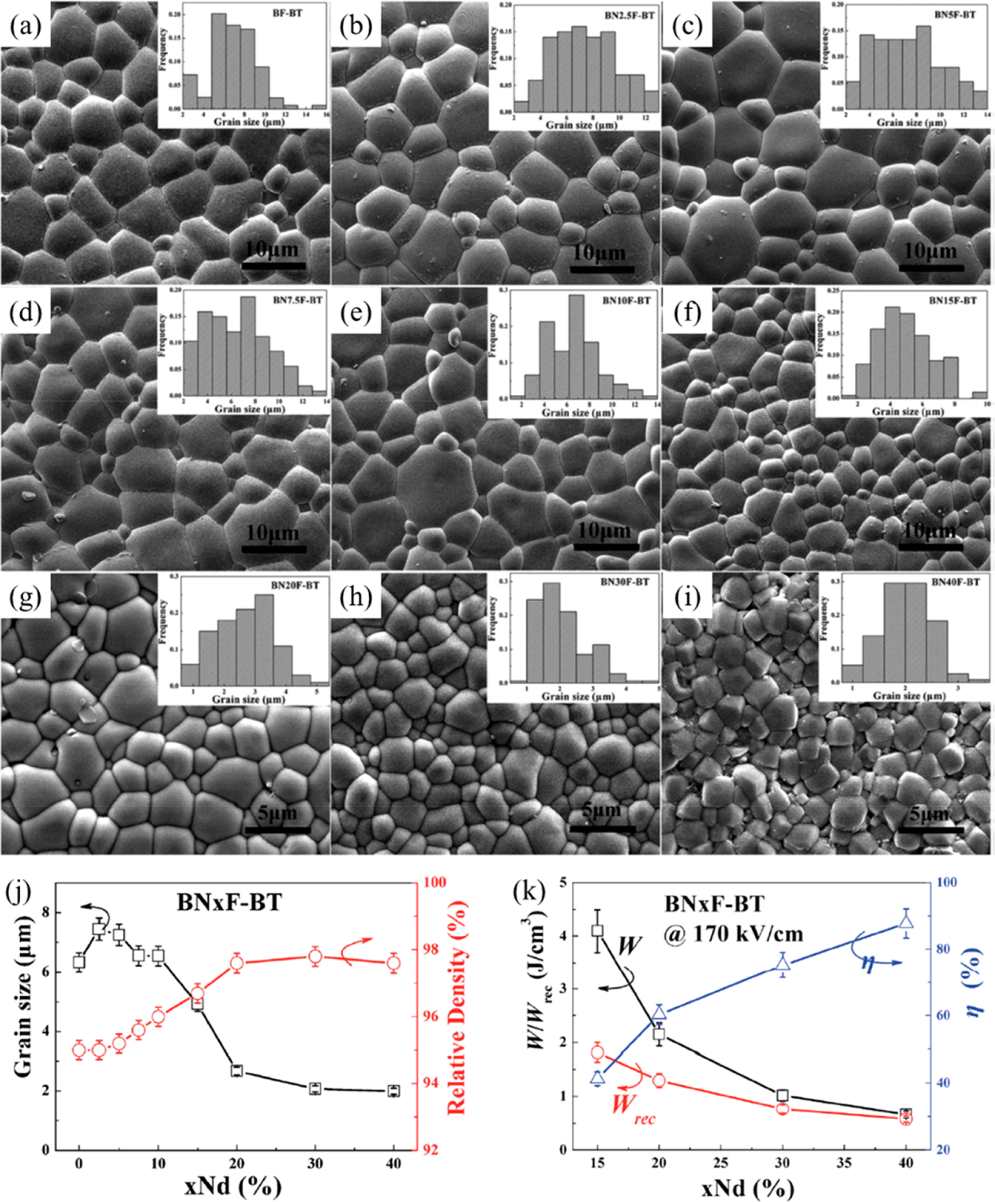
SEM images of *x* mol % Nd-doped BF–BT with different Nd concentrations: (a) BF–BT, (b) 2.5 mol % Nd–BF–BT, (c) 5 mol % Nd–BF–BT, (d) 7.5 mol % Nd–BF–BT, (e) 10 mol % Nd–BF–BT, (f) 15 mol % Nd–BF–BT, (g) 20 mol % Nd–BF–BT, (h) 30 mol % Nd–BF–BT, and (i) 40 mol % Nd–BF–BT; the *G* distributions of Nd-doped BF–BT are shown in the insets of the SEM images.^90^ (j) *G*, density and (k) energy storage performance at 170 kV cm^–1^, as a function of *x*(Nd) mol % in BF–BT ceramics. Reproduced with permission from ref (90). Copyright 2017 Royal Society of Chemistry.

For example, an average *G* ∼ 10 μm was reported for BF–BT ceramics, which was reduced to <2 μm after A-site Nd doping, as shown in Figure 7. Meanwhile, improved *W*_rec_ ∼ 1.8 J cm^–3^ and η ∼ 88% were obtained for 15 mol % Nd–BF–BT and 40 mol % Nd–BF–BT, respectively.^90^ Similar optimization behavior has also been found in KNN–BMN and KNN–ST ceramics, resulting in BDS ∼ 400 kV cm^–1^ and *W*_rec_ > 3.5 J cm^–3^.^91,92^

#### 2.3.5 Core–Shell Structure

Core–shell subgrain microstructures are observed in many lead-free ceramics, due to either kinetic limitations of the diffusion process (typical for BT based ceramics) or immiscibility on cooling from high temperature for perovskite end members with dissimilar ion size and bonding (BF based ceramics).^22,34,45^ The effect of core–shell microstructures on energy storage performance is still unclear. In BT-based ceramics, the cores are often more conducting than the doped shells and core to core conductive pathways lead to breakdown.^93−96^ For BF based ceramics, the defect chemistry of the cores and shells remains to be elucidated, but initial work suggests that further dopants are needed to create electrical homogeneity and thus eliminate the conducting pathways.^34,45^ The theoretical modeling has reported a positive influence of core–shell microstructure but none have been unambiguously validated experimentally.^97^

## 3 State-of-the-Art in Electroceramics for Energy Storage

### 3.1 Bulk Ceramics

#### 3.1.1 Lead-Based Ceramics

Lead-based ceramics are used commercially as energy storage materials for high-power pulsed capacitors due to their excellent *W*_rec_ and η.^98−101^ The energy storage properties of RFE and AFE lead-based ceramics are summarized in Table 3.

**Table 3:** Summary of Energy Storage Properties for Lead-Based Ceramics^a^

compositions	*E* (kV cm^–1^)	*ΔP* (μC cm^–2^)	*W*_rec_ (J cm^–3^)	η (%)	ref
(Pb_0.89_Ba_0.08_La_0.02_)(Zr_0.7_Sn_0.27_Ti_0.03_)O_3_	135	22.6	2.1	76.5	(150)
(Pb_1.067_La_0.02_)(Zr_0.95_Ti_0.05_)O_3_	90	39.5	2.12	92.98	(141)
0.90(Pb_0.97_La_0.02_)(Zr_0.65_Sn_0.30_Ti_0.05_)O_3_–0.10Bi(Zn_2/3_Nb_1/3_)O_3_	115	29	2.19	95.6	(162)
Pb_0.97_La_0.02_(Zr_0.58_Sn_0.35_Ti_0.07_)O_3_	118	29.0	2.35	86.1	(158)
Pb_0.91_La_0.02_Ba_0.06_(Zr_0.65_Sn_0.3_Ti_0.05_)O_3_	150	29.5	2.4	82	(159)
(Pb_0.93_Ba_0.04_La_0.02_)(Zr_0.65_Sn_0.3_Ti_0.05_)O_3_–0.005Mn_2_O_3_	308	31.5	2.64	73	(161)
Pb_0.97_La_0.02_(Zr_0.33_Sn_0.55_Ti_0.12_)O_3_@0.05SiO_2_	238	34.6	2.68	83.5	(87)
(Pb_0.87_Ba_0.1_La_0.02_)(Zr_0.65_Sn_0.3_Ti_0.05_)O_3_–0.75Y	130	46.5	2.75	71.5	(149)
(Pb_0.88_La_0.08_)(Zr_0.91_Ti_0.09_)O_3_	170	31.5	3.04	92	(112)
1.7 mol % Pr^3+^ doped 0.24Pb (In_1/2_Nb_1/2_)O_3_–0.42Pb(Mg_1/3_Nb_2/3_)O_3_-0.34PbTiO_3_	50	20	3.1	90	(109)
0.92Pb(Tm_0.5_Nb_0.5_)O_3_–0.08Pb(Mg_1/3_Nb_2/3_)O_3_	310	17.03	3.12		(136)
(Pb_0.87_Ba_0.1_La_0.02_)(Zr_0.68_Sn_0.24_Ti_0.08_)O_3_	180	58.2	3.2		(151)
Pb_0.97_La_0.02_(Zr_0.50_Sn_0.46_Ti_0.04_)O_3_	150	43	3.2	86.5	(124)
0.55(Pb_0.97_La_0.02_)(Zr_0.93_Sn_0.05_Ti_0.02_)O_3_–0.45(Pb_0.93_Ba_0.04_La_0.02_) (Zr_0.65_Sn_0.3_Ti_0.05_)O_3_	180	25	3.2	74.4	(160)
Pb_0.97_La_0.02_(Zr_0.56_Sn_0.35_Ti_0.09_)O_3_	175	39.4	3.3	80	(166)
(Pb_0.895_La_0.07_)(Zr_0.9_Ti_0.1_)O_3_	175	42.3	3.38	86.5	(115)
0.9PbHfO_3_–0.1Pb(Mg_0.5_W_0.5_)O_3_	155	43.5	3.7	72.5	(134)
Pb_0.94_La_0.04_(Lu_0.5_Nb_0.5_)O_3_	681		3.85		(137)
(Pb_0.955_La_0.03_)(Zr_0.50_Sn_0.42_Ti_0.08_)O_3_	180	41	3.99	79.2	(127)
Pb_0.97_La_0.02_(Zr_0.60_Sn_0.35_Ti_0.05_)O_3_	200	34.48	4.1		(121)
(Pb_0.97_La_0.02_)(Zr_0.5_Sn_0.44_Ti_0.06_)O_3_	250	29.3	4.2	82	(117)
(Pb_0.97_La_0.02_)(Zr_0.5_Sn_0.44_Ti_0.06_)O_3_	250	29.3	4.2	82	(118)
Pb_0.955_La_0.03_(Zr_0.5_Sn_0.43_Ti_0.07_)O_3_	200	36	4.2	78	(126)
(Pb_0.97_La_0.02_)(Zr_0.8_Sn_0.145_Ti_0.055_)O_3_	225	34	4.38	73	(124)
(Pb_0.858_Ba_0.1_La_0.02_Y_0.008_)(Zr_0.65_Sn_0.3_Ti_0.05_)O_3_- (Pb_0.97_La_0.02_)(Zr_0.9_Sn_0.05_Ti_0.05_)O_3_	200	46.8	4.65	60	(152)
La_0.02_Pb_0.97_[(Yb_0.5_Nb_0.5_)_0.92_Ti_0.08_]O_3_	240	34	5.18	65	(135)
(Pb_0.97_La_0.02_Zr_0.85_Sn_0.12_Ti_0.03_O_3_)−0.5 wt % Al_2_O_3_	315	35.5	5.3	88.3	(72)
(Pb_0.955_Sr_0.015_La_0.02_)(Zr_0.75_Sn_0.195_Ti_0.055_)O_3_	350	33.5	5.56	70	(156)
Pb_0.97_La_0.02_(Zr_0.5_Sn_0.45_Ti_0.05_)O_3_	400	36.2	5.6	63	(116)
(Pb_0.858_Ba_0.1_La_0.02_Y_0.008_)(Zr_0.65_Sn_0.3_Ti_0.05_)O_3_–(Pb_0.97_La_0.02_)(Zr_0.9_Sn_0.05_Ti_0.05_)O_3_	306	48.5	6.4	62.4	(173)
Pb[(Lu_0.5_Nb_0.5_)–(Mg_0.5_W_0.5_)]O_3_	340	46	6.4	71	(132)
Pb_0.91_La_0.06_(Zr_0.552_Sn_0.368_Ti_0.08_)O_3_@1 wt %PbO–B_2_O_3_–SiO_2_–Al_2_O_3_–ZnO–MnO_2_	380	43	7.4	91.6	(77)
PbHfO_3_	270	44.5	7.6	80.8	(133)
Pb_0.98_La_0.02_(Hf_0.45_Sn_0.55_)_0.995_O_3_	380	36	7.63	94	(138)
(Pb_0.91_La_0.06_)(Zr_0.96_Ti_0.04_)O_3_–1.0 mol % MnCO_3_	300	43.5	7.65	87	(145)
(Pb_0.98_La_0.02_)(Zr_0.55_Sn_0.45_)_0.995_O_3_	400	41.5	10.4	87	(131)
(Pb_0.94_La_0.02_Sr_0.04_)(Zr_0.9_Sn_0.1_)_0.995_O_3_	400	44	11.18	82.2	(130)

a *t* of the bulk ceramics is commonly >0.1 mm.

##### 3.1.1.1 Lead-Based Relaxor-Ferroelectrics

**Figure 8:**
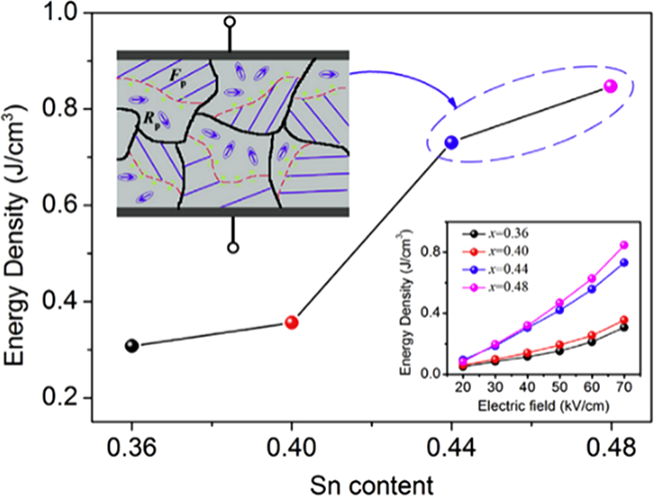
*W*_rec_ of 0.2PMN–0.8PS_*x*_T_1–*x*_ ceramics with different Sn (*x*) contents at 70 kV cm^–1^. The insets show the mechanism of enhanced energy storage due to coexistent-phase structure and the *W*_rec_ for PMN–PS_*x*_T_1–*x*_ ceramics under different electric fields. Reproduced with permission from ref (111). Copyright 2018 Elsevier.

Many lead-based RFEs, including Pb(Mg_1/3_Nb_2/3_)O_3_–PbTiO_3_ (PMN–PT), Pb(Zn_1/3_Nb_2/3_)O_3_–PbTiO_3_ (PZN–PT), and (Sr,Pb,Bi)TiO_3_ (SPBT)-based materials, have been reported as potential candidates for energy storage capacitors.^102−110^ Zhang and co-workers investigated the relaxation behavior and energy storage properties of (1–*x*)PMN–*x*PT ceramic, obtaining *W*_rec_ ∼ 0.47 J cm^–3^ at room temperature.^102^ Li and co-worker probed the effect of domain structure on *W*_rec_ and thermal stability of 0.2PMN–0.8Pb(Sn_*x*_Ti_1–*x*_)O_3_ (PMN–PS_*x*_T_1–*x*_) ceramics, as illustrated in Figure 8. 0.2PMN–0.8PST ceramics exhibited *W*_rec_ ∼ 0.85 J cm^–3^ with excellent thermal stability which was attributed to the coexistence of ferroelectric domains and PNRs.^111^

##### 3.1.1.2 Lead-Based Antiferroelectrics

PbZrO_3_ (PZ) is the first known AFE and exhibits a double *P–E* hysteresis loop below *T*_C_. However, the high critical switching field required for an AFE–FE phase transition at room temperature limits applications for energy storage. Chemical substitution to reduce switching field is an effective strategy to overcome the problem and three well-known PbZrO_3_ based compositions are reviewed: (i) (Pb,La)(Zr,Ti)O_3_ (PLZT);^112−115^ (ii) (Pb,La)(Zr,Sn,Ti)O_3_ (PLZST);^116−129^ and (iii) (Pb,La)(Zr,Sn)O_3_ (PLZS).^130,131^ Additionally, some new AFEs have also been identified based on PbHfO_3_, Pb(Lu_0.5_Nb_0.5_)O_3_, Pb(Yb_0.5_Nb_0.5_)O_3_, and Pb(Tm_0.5_Nb_0.5_)O_3_.^132−138^

##### 3.1.1.2.1 (Pb,La)(Zr,Ti)O_3_ (PLZT)

**Figure 9:**
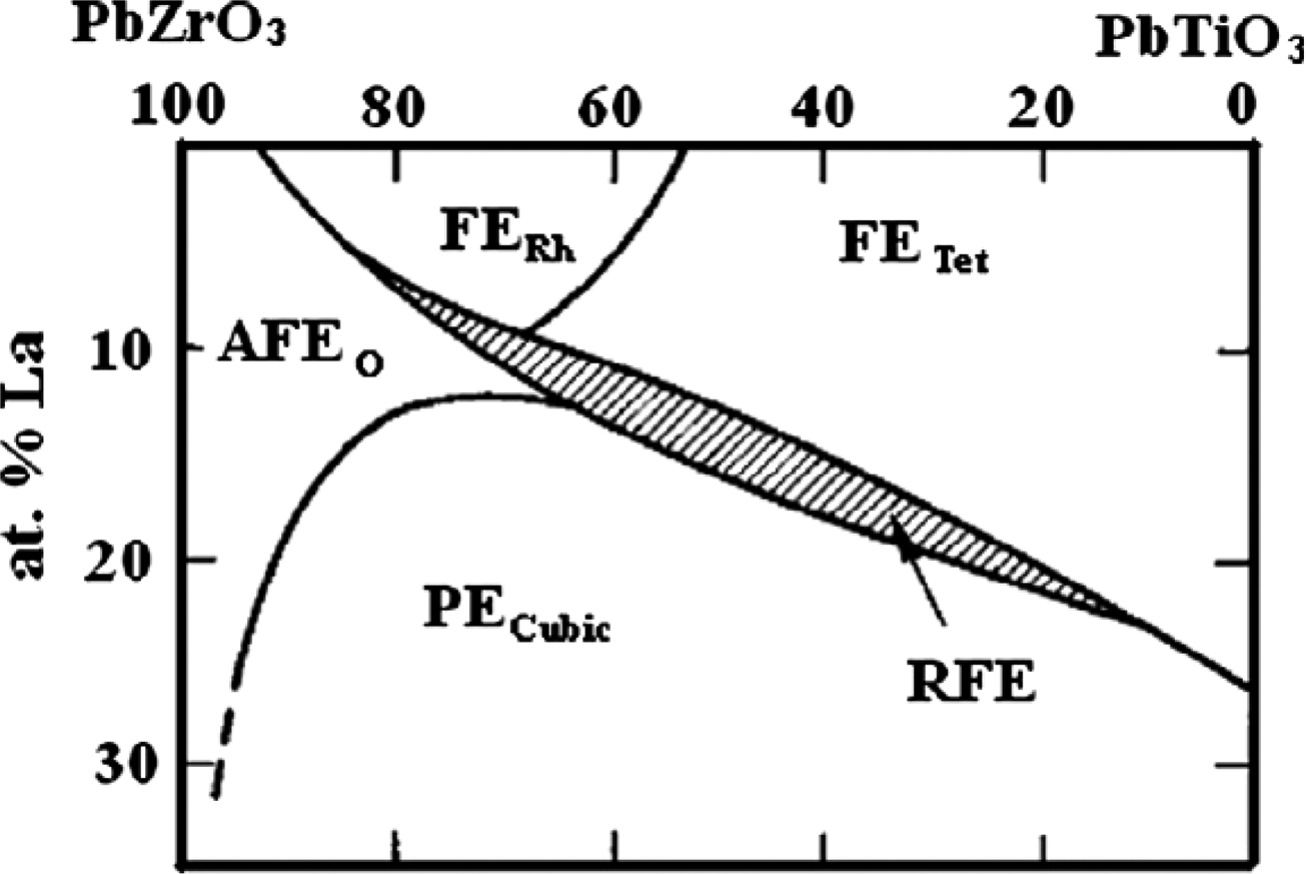
Phase diagram of PLZT at room temperature.^139^ Reproduced with permission from ref (139). Copyright 2014 Elsevier.

According to the phase diagram (Figure. 9), PLZT exists as homogeneous compositions over a wide range of mol % La in the PbZrO_3_–PbTiO_3_ solid solution.^139,140^

**Figure 10:**
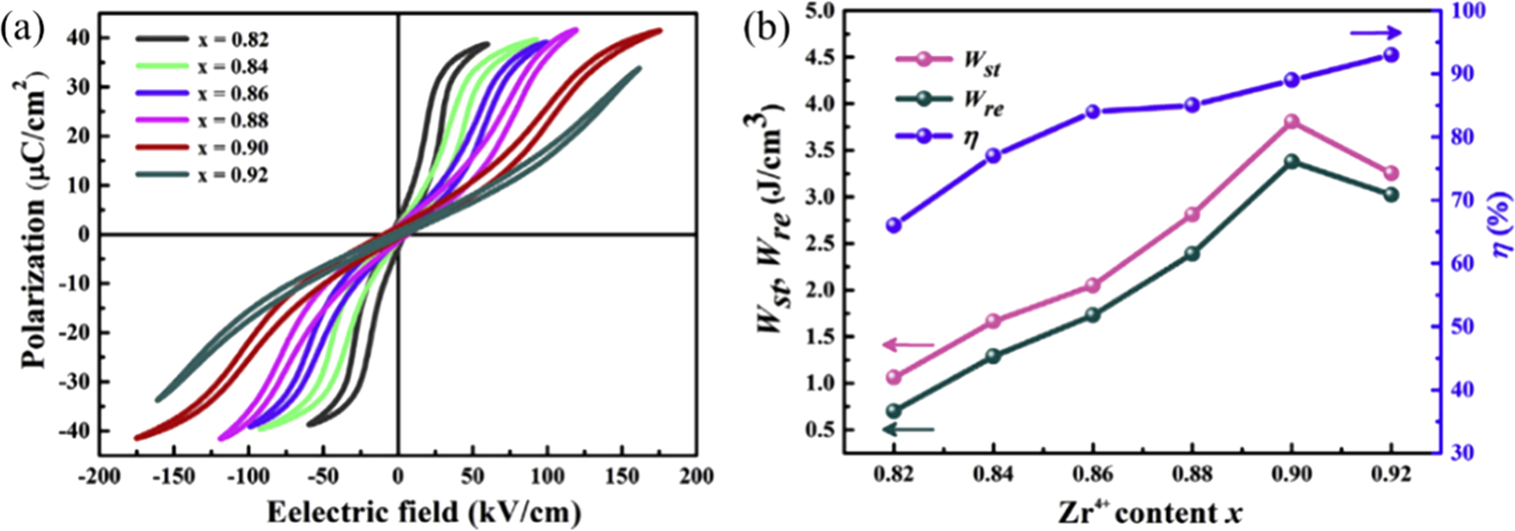
Effect of Zr/Ti ratio on *P*–*E* loops and energy storage properties of PLZT.^115^ Reproduced with permission from ref (115). Copyright 2019 Elsevier.

When Pb ions are replaced by ≤30 mol % La on the A-site in accordance with a lead vacancy (*V*_*pb*_^..^) ionic compensation model, an orthorhombic AFE phase similar to PbZrO_3_ occurs for Zr rich compositions. However, only PLZT with <10 mol % La is typically utilized for energy storage applications^141−147^ since higher concentrations have lower polarization and therefore lower *W*_rec_. Li and co-workers prepared (Pb_0.97_La_0.02_)(Zr_0.95_Ti_0.05_)O_3_ ceramics via a solid-state reaction route, yielding *W*_rec_ ∼ 0.83 J cm^–3^ and η ∼ 70% under an electric field of 55 kV cm^–1^.^114^ Jo and co-workers found that AFE and RFE behavior can both be obtained by substitution of La and excess PbO in PLZT, resulting in the enhancement of *W*_rec_ by promoting a slim and slanted hysteresis loop. Both high *W*_rec_ ∼ 3.04 J cm^–3^ and η ∼ 92% were obtained along with no performance degradation after 10^5^ cycles.^112^ Tuning the Zr/Ti ratio has also shown to be an effective way to improve *W*_rec_ of PLZT ceramics. Qiao and co-workers reported slimmer *P–E* loops giving a *W*_rec_ ∼ 3.38 J cm^–3^ in (Pb_0.895_La_0.07_)(Zr_*x*_Ti_1–*x*_)O_3_ ceramics by changing the Zr/Ti ratio (Figure 10), which was attributed to the reduction of tolerance factor and “enhancement of antiferroelectricity”.^115^ Mn doping is also suggested to improve *W*_rec_ of PLZT by “enhancing antiferroelectricity”.^143−145^

##### 3.1.1.2.2 (Pb,La)(Zr,Sn,Ti)O_3_ (PLZST)

**Figure 11:**
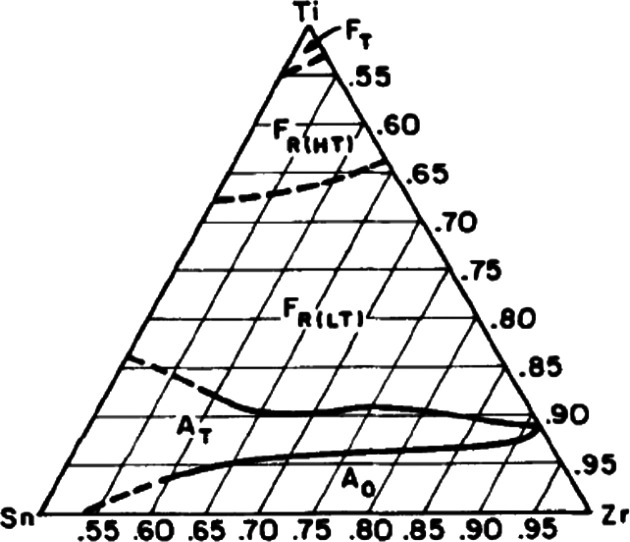
Phase diagram of (Pb_0.97_La_0.02_)(Zr,Sn,Ti)O_3_, where T, R, and O represent the tetragonal, rhombohedral, and orthorhombic structure, respectively, and HT and LT represent high and low temperature, respectively. Reproduced with permission from ref (167). Copyright 2005 John Wiley and Sons.

To further optimize the energy storage properties of PLZT ceramics, Sn may be substituted on the B-site of PLZT, which broadens the AFE compositional range,^72,77,87,116,122,148−166^ in accordance with phase diagram from 1989 (Figure 11).^167^ (Pb_0.97_La_0.02_)(Zr,Sn,Ti)O_3_ with 2 mol % La has been most commonly studied in which the Zr/Ti/Sn ratio is varied to give a complex phase diagram that contains FE tetragonal (F_T_), high-temperature nontilted FE rhombohedral (F_R(HT)_), low temperature FE rhombohedral (F_R(LT)_) AFE tetragonal (A_T_), and AFE orthorhombic (A_O_) phases.

**Figure 12:**
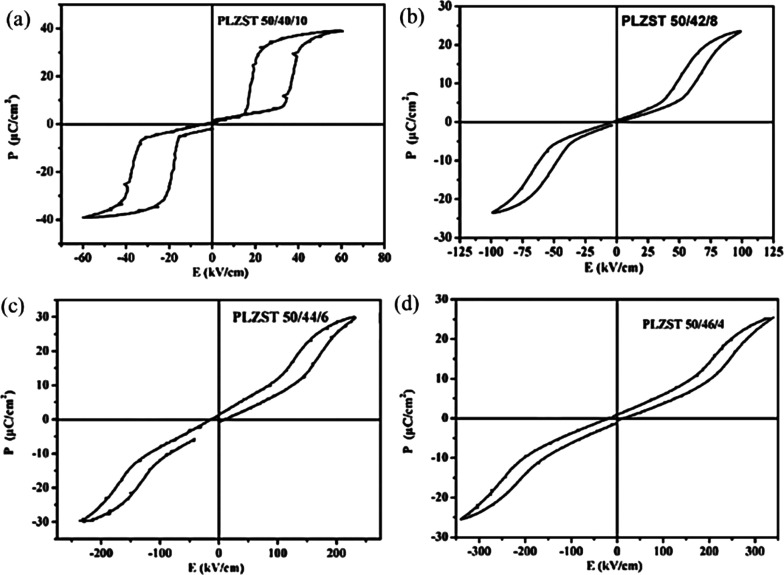
AFE-type *P–E* hysteresis loops of Pb_0.97_La_0.02_(Zr_0.5_Sn_0.5–*x*_Ti_*x*_)O_3_ with *x* = (a) 0.10, (b) 0.08, (c) 0.06, and (d) 0.04.^118^ Reproduced with permission from ref (118). Copyright 2016 Elsevier.

We note that the A_T_ phase in both PLZT and PLZST has been shown to be incommensurate by a number of researchers and might be better described as a A_O_ phase in which there is disorder of antipolar coupling.^168−170^ PLZST is AFE for concentrations with <15 mol % Ti. For compositions with A_O_ structure, the critical phase switching fields are above BDS but ceramics with the A_T_ phase undergo electric field-induced AFE-FE switching at room temperature, for which the switching field increases with increasing Sn concentration (Figure 12).^118^ Adjusting the Zr/Sn/Ti ratio leads to optimization of *W*_rec_ in PLZST ceramics^116,118,148,157^ with an increase in Sn concentration leading to a reduction in switching field (forward threshold electric field, *E*_F_, and backward threshold electric field, *E*_B_) and weakening ferroelectricity.^171^*W*_rec_ ∼ 5.6 J cm^–3^ and high thermal stability have been obtained in PLZST ceramics with a Zr/Sn/Ti ratio of 0.5:0.45:0.05^116^ while Wang and co-workers reported, in their study of the (Pb_0.97_La_0.02_)(Zr_0.5_Sn_0.5-*x*_Ti_*x*_)O_3_ solid solution, superior *W*_rec_ of 4.2 J cm^–3^ with η of 82% for (Pb_0.97_La_0.02_)(Zr_0.5_Sn_0.44_Ti_0.06_)O_3_ ceramics with good temperature stability.^118^ Recently, a ferrielectric (FIE) configuration was reported in PLZST which consists of ferroelectric ordering segments with either magnitude or angular modulation of dipoles.^172^ The net polarization of field-induced FE order can be tailored by adjusting the Sn/Ti ratio.

The performance of PLZST ceramics is also influenced by the Pb/La ratio.^117,122,126^ The AFE phase becomes more stable with a commensurate increase in the AFE–FE switching field as La concentration increases. The energy storage properties of (Pb_1–1.5*x*_La_*x*_)(Zr_0.5_Sn_0.43_Ti_0.07_)O_3_ ceramics were optimized (*W*_rec_ of 4.2 J cm^–1^) by Dan and co-workers for compositions with *x* = 0.03 due to a large switching electric field and high BDS.^3,126^ Furthermore, doping with Ba and Sr (A-site) improves fatigue behavior and temperature stability, suppresses the stress sensitivity, and enhances energy storage.^148,150−153,155,161,173^

##### 3.1.1.2.3 (Pb,La)(Zr,Sn)O_3_ (PLZS)

**Figure 13:**
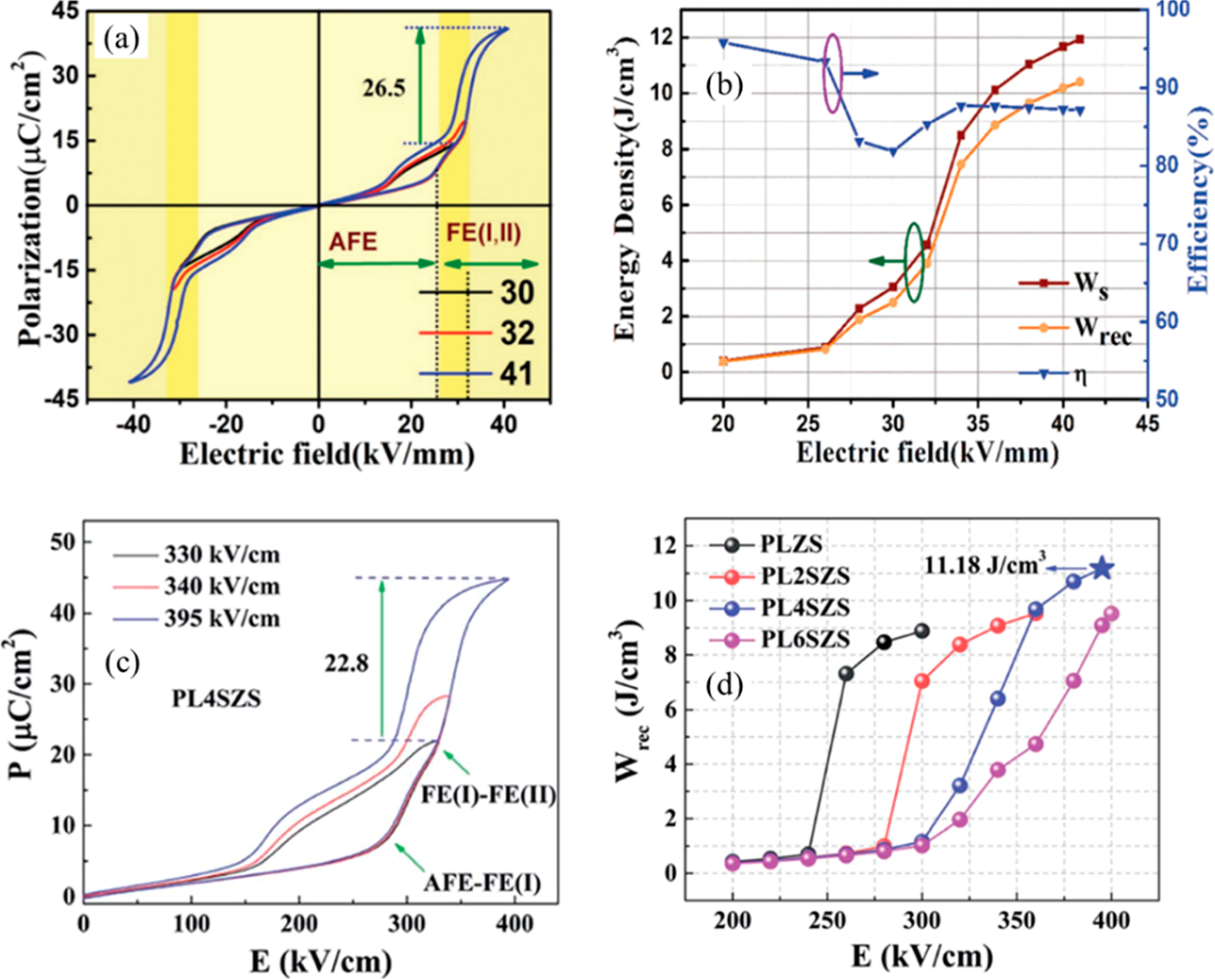
(a) Bipolar *P–E* hysteresis loops and (b) energy storage properties of (Pb_0.98_La_0.02_)(Zr_0.55_Sn_0.45_)_0.995_O_3_ ceramics under different applied fields.^131^ (c) Unipolar *P–E* hysteresis loops of the (Pb_0.94_La_0.02_Sr_0.04_)(Zr_0.9_Sn_0.1_)_0.995_O_3_ ceramic under different applied fields. (d) *W*_rec_ of (Pb_0.98-*x*_La_0.02_Sr_*x*_)(Zr_0.9_Sn_0.1_)_0.995_O_3_ with Sr concentration (*x* = 0–0.06) as a function of the *E*.^130^ (a, b) Reproduced with permission from ref (131). Copyright 2019 John Wiley and Sons; (c, d) Reproduced with permission from ref (130). Copyright 2019 Royal Society of Chemistry.

Wang and co-workers reported a unique *E*-induced multiphase transition in PLZS for which a conventional AFE–FE phase transition at low *E*, followed by a second FE-FE phase transition at a higher *E*, leads to an increase in polarization.^131^*W*_rec_ of 10.4 J cm^–3^ and η of 87% were achieved at 400 kV cm^–1^ for (Pb_0.98_La_0.02_)(Zr_0.55_Sn_0.45_)_0.995_O_3_ ceramics, along with superior discharge current density of 1640 A cm^–2^ and ultrafast discharge speed (75 ns discharge period) (Figure 13a,b).^131^ Subsequently, a record-high *W*_rec_∼ 11.2 J cm^–3^ and a high η ∼ 82% were realized in (Pb_0.98–*x*_La_0.02_Sr_*x*_)(Zr_0.9_Sn_0.1_)_0.995_O_3_ ceramics, as illustrated in (Figure 13c,d). The substitution of Pb by Sr gave rise to an increase in BDS and AFE/FE switching fields, leading to further enhancement of energy storage performance.^130^

#### 3.1.2 Lead-Free Ceramics

In the last decades, extensive research has focused on lead-free electroceramics due to concerns over the toxicity of lead/lead oxide-based compounds.^174−176^ As a result, there has been a steady but continuous improvement in their energy storage performance,^31,103,177−180^ with a view to replacing existing lead-based materials. Several lead-free ceramic systems are considered as potential candidates to replace PLZT for energy storage applications, including those based on BT, ST, KNN, BF, NBT, AgNbO_3_ (AN), and NN.

##### 3.1.2.1 BaTiO_3_-Based Ceramics

BT-based dielectric ceramics have been studied for decades and dominate the commercial market of ceramic capacitors.^81,181^ Several studies have reported improvements in energy storage performance of BT-based ceramics through (i) substituting oxides to improve BDS, such as Al_2_O_3_, La_2_O_3_, MgO, SiO_2_;^79,80,182−184^ (ii) employing different sintering techniques, such as SPS, citrate precursor, and cold sintering (CS) to increase ceramic density or control grain growth;^68,76,185^ (iii) adding sintering aids such as ZnNb_2_O_6_ and NiNb_2_O_6_ to increase density;^186,187^ (iv) introducing further end-members in the solid solution, Bi(Mg_1/2_Ti_1/2_)O_3_,^188,189^ BiYbO_3_,^190^ BMN,^191−193^ NBT–Na_0.73_Bi_0.09_NbO_3_,^194^ Nd(Zn_1/2_Ti_1/2_)O_3_,^195^ Bi(Zn_2/3_Nb_1/3_)O_3_,^196^ Bi(Li_1/2_Nb_1/2_)O_3_,^197,198^ Bi(Zn_1/2_Zr_1/2_)O_3_,^199^ Bi(Zn_1/2_Ti_1/2_)O_3_,^200^ YNbO_4_,^201^ Bi_0.9_Na_0.1_In_0.8_Zr_0.2_O_3_,^202^ Bi(Li_1/2_Ta_1/2_)O_3_,^203^ Bi(M*g*_1/2_Zr_1/2_)O_3_,^204^ Bi(Zn_1/2_Sn_1/2_)O_3_,^205^ K_0.5_Bi_0.5_TiO_3_–KNbO_3_,^206^ and K_0.5_Bi_0.5_TiO_3_–NN^207^ to promote RFE behavior. The energy storage properties of BT-based materials are summarized in Table 4.

**Table 4:** Energy Storage Properties of BT-Based Materials^a^

compd	*E* (kV cm^–1^)	*ΔP* (μC cm^–2^)	*W*_rec_ (J cm^–3^)	η (%)	ref
0.9BT–0.1BMN	143.5	∼16	1.13	95	(192)
Ba_0.85_Ca_0.15_Zr_0.1_Ti_0.9_O_3_	200	∼15	1.153		(182)
0.9BT–0.1(Bi_0.9_Na_0.1_)(In_0.8_Zr_0.2_)O_3_	180	∼20	1.33	88	(202)
0.88(Ba_0.8_Sr_0.2_)TiO_3_–0.12 Bi(Zn_2/3_Nb_1/3_)O_3_	225	17	1.62	99.8	(210)
0.9BT–0.1BMN–0.3 wt % MnCO_3_	205	16.28	1.7	88.5	(191)
0.92(0.65BT–0.35NBT)–0.08(Na_0.73_Bi_0.09_NbO_3_)	172	∼25	1.7	82	(194)
0.92(0.92BT–0.08K_0.5_Bi_0.5_TiO_3_)–0.08NN	220	∼23	1.96	67.4	(207)
0.88BT–0.12Bi(Li_0.5_Nb_0.5_)O_3_	270	∼19	2.03	88	(197)
0.9BT–0.1Bi(Li_0.5_Ta_0.5_)O_3_	280	∼11.9	2.2	88	(203)
0.85BT–0.15Bi(Zn_0.5_Sn_0.5_)O_3_	280	∼23	2.41	91.6	(205)
0.9BT–0.1Bi(Zn_0.5_Zr_0.5_)O_3_	264	∼25	2.46		(199)
0.85BT–0.15Bi(Mg_0.5_Zr_0.5_)O_3_	280	∼23	2.9	86.8	(204)
0.9Ba_0.65_Sr_0.35_TiO_3_–0.1BMN	400	23	3.34	85.71	(211)
0.65(Ba_0.98_Li_0.04_)Ti_0.98_O_3_–0.35(Sr_0.7_Bi_0.2_)TiO_3_	410	35	3.54	77	(212)
0.6BT–0.4Bi(Mg_0.5_Ti_0.5_)O_3_	340	∼40	4.49	93	(208)
BT–0.06Bi_2/3_(Mg_1/3_Nb_2/3_)O_3_	520	25	4.55	91	(209)

a *t* of the bulk ceramics is commonly >0.1 mm.

The most effective approach, however, is the introduction of a Bi-based perovskite end member in which the B-site contains multiple cations. Doping with a Bi ion that has a lone pair electronic 6s^2^ configuration into the Ba-site increases *P*_max_. *P*_r_ is minimized by forming a so-called “weakly coupled relaxor” state in which long-range polar coupling is disrupted by the multiple B-site ions which have different valence and ionic radius to Ti. Using this principle, Hu and co-workers^208^ reported high *W*_rec_ of 4.49 J cm^–3^ with a η of 93% for 0.6BT–0.4 Bi(Mg_0.5_Ti_0.5_)O_3_ (BT–BMT) ceramics that were temperature stable to 170 °C (variation *W*_rec_ < 5% and η < 4%).

**Figure 14:**
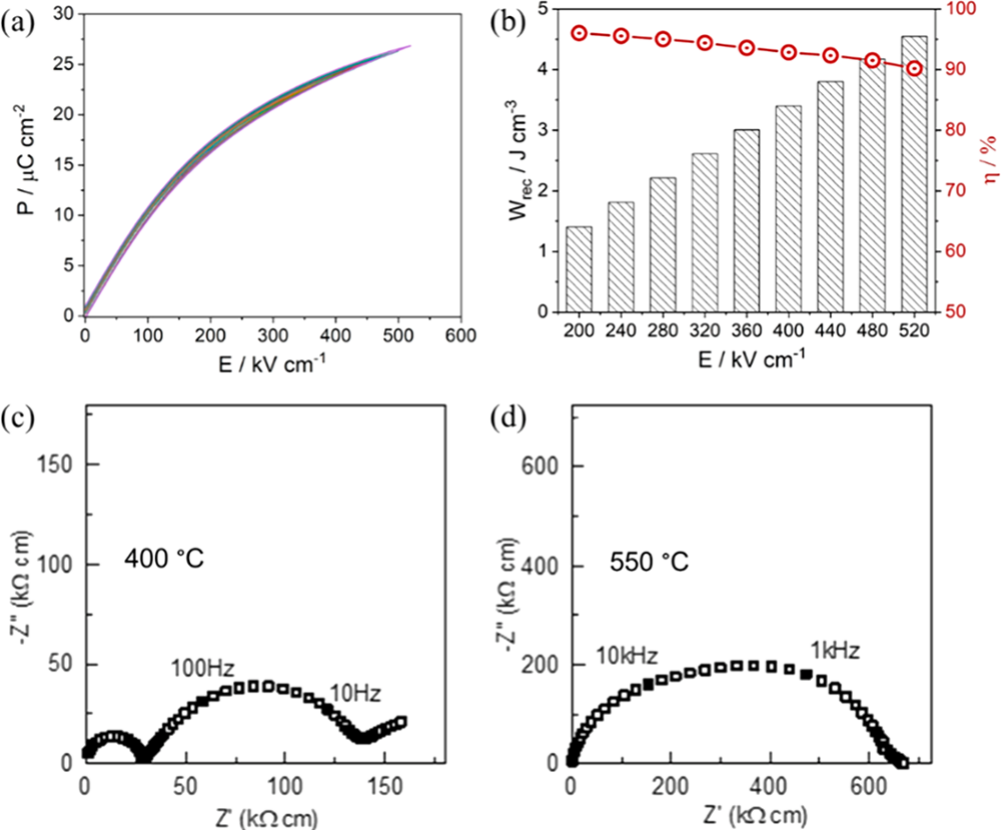
(a) Unipolar *P–E* loops under *E*_max_ and (b) calculated *W*_rec_ and η at different electric field for BT–0.06B_2/3_MN ceramics. (c) Z* plots of (c) BT at 400 °C and (d) BT–0.06B_2/3_MN at 550 °C.^209^ Reproduced from ref (209). Copyright 2020 American Chemical Society.

Of greater commercial potential, Yang and co-workers reported similar properties with BT–0.06 Bi_2/3_(Mg_1/3_Nb_2/3_)O_3_ (BT–0.06B_2/3_MN) but in compositions compatible with Ag/Pd electrodes due to the presence of only 4 mol % Bi on the A-site.^209^ Similar energy storage properties, *W*_rec_ ∼ 4.6 J cm^–3^ and η ∼ 92% (Figures 14a,b) to BT–BMT were obtained for BT–0.06B_2/3_MN ceramics which also benefited from the highest BDS, ∼520 kV cm^–1^, among all reported BT-based compositions.^209^ BT–0.06B_2/3_MN is not electrically homogeneous but BDS and *W*_rec_ were still optimized by reducing, though not completely eliminating, the difference between the bulk and grain boundary responses with respect to undoped BT, Figures 14c,d.^209^

##### 3.1.2.2 SrTiO_3_-Based Ceramics

ST, which is an incipient ferroelectric, is considered as a promising candidate for energy storage applications due to its relatively high permittivity (*ε*_r_ ∼ 300) and low dielectric loss (<1%) at room temperature. Extensive efforts have been made to improve the energy storage performance of ST-based ceramics, including (i) doping with Ba,^213−216^ Dy,^217^ Mg,^218^ Ce,^64^ and Bi^219−222^ on the A-site or Mn^223^ and Sn^224,225^ on the B-site; (ii) using sintering aids, such as ZnO,^65^ MgO,^226−228^ SiO_2_,^63,229^ SrO–B_2_O_3_–SiO_2_,^230^ ZnO–Li_2_O,^231^ Bi_2_O_3_–B_2_O_3_–SiO_2_,^232^ B_2_O_3_–SiO_2_–Bi_2_O_3_–CaO–BaO,^233^ Al_2_O_3_,^234^ BaO–TiO_2_–SiO_2_,^75^ BaCu(B_2_O_5_),^235^ and NiNb_2_O_6_;^236^ (iii) employing different sintering techniques such as microwave sintering^73,75^ and SPS;^70^ and (iv) introducing complex end-members, such as NBT,^237,238^ NBT–Ba(Al_0.5_Nb_0.5_)O_3_,^239^ NBT–BT,^118,240,241^ and Bi(Mg_0.5_Hf_0.5_)O_3_.^242^ The energy storage properties of ST-based materials are summarized in Table 5.

**Table 5:** Energy Storage Properties of ST-Based Materials^a^

compounds	*E* (kV cm^–1^)	*ΔP* (μC cm^–2^)	*W*_rec_(J cm^–3^)	η (%)	ref
95 wt % Ba_0.4_Sr_0.6_TiO_3_–5 wt %MgO	300	11.88	1.5	88.5	(227)
Ba_0.4_Sr_0.6_TiO_3_–8 mol % SiO_2_	400	9.11	1.6	90.9	(229)
0.6ST–0.4NBT	210	∼20	1.7	69.93	(237)
0.45ST–0.2NBT–0.35BT	170	∼25	1.78	77.06	(243)
(Sr_0.99_Mg_0.01_)TiO_3_	362	∼12	1.86	89.3	(218)
0.5ST–0.5(0.94 Bi_0.54_Na_0.46_TiO_3_–0.06BT)	180	∼30	1.88	79	(239)
0.5ST–0.5(0.95NBT–0.05BaAl_0.5_Nb_0.5_O_3_)	190	∼28	1.89	77	(240)
Ba_0.4_Sr_0.6_TiO_3_–9 wt %(Bi_2_O_3_–B_2_O_3_–SiO_2_)	279	∼17	1.98	90.57	(232)
0.95(Sr_0.5_Na_0.25_Bi_0.25_TiO_3_)–5 wt %MgO–0.05KNbO_3_	178.5	∼27	2	76.34	(244)
Ba_0.4_Sr_0.6_(Ti_0.996_Mn_0.004_)O_3_–2 wt % MgO	300	∼16	2.014	88.6	(228)
Ba_0.3_Sr_0.6_Ca_0.1_TiO_3_–2 wt %MgO	194.33	∼23	2.223		(245)
Sr_0.985_Ce_0.01_TiO_3_–3 wt % SiO_2_	290	∼10	2.23		(64)
SrTi_0.985_(Zn_1/3_Nb_2/3_)_0.015_O_3_–4.5 wt %ZnNb_2_O_6_	422	9.34	2.35	77	(246)
0.9ST–0.1(Bi_0.48_La_0.02_Na_0.48_Li_0.02_Ti_0.98_Zr_0.02_O_3_)	323	∼20	2.59	85	(247)
0.8ST–0.2(NBT– Ba_0.94_La_0.04_Zr_0.02_Ti_0.98_O_3_)	320	∼22	2.83	85	(248)
0.995(0.6ST−0.4NBT)−0.005ZrO_2_	285	∼25	2.84	71.54	(74)
0.9(Sr_0.7_Bi_0.2_)TiO_3_–0.1 Bi(Mg_0.5_Hf_0.5_)O_3_	360	∼22	3.1	93	(242)
Ba_0.3_Sr_0.7_TiO_3_–1.6 wt % ZnO	400		3.9		(65)
98.5 wt %Ba_0.4_Sr_0.6_TiO_3_–1.254 wt %Al_2_O_3_–0.246 wt %SiO_2_	493		5.09		(234)

a The *t* of the bulk ceramics is commonly >0.1 mm.

**Figure 15:**
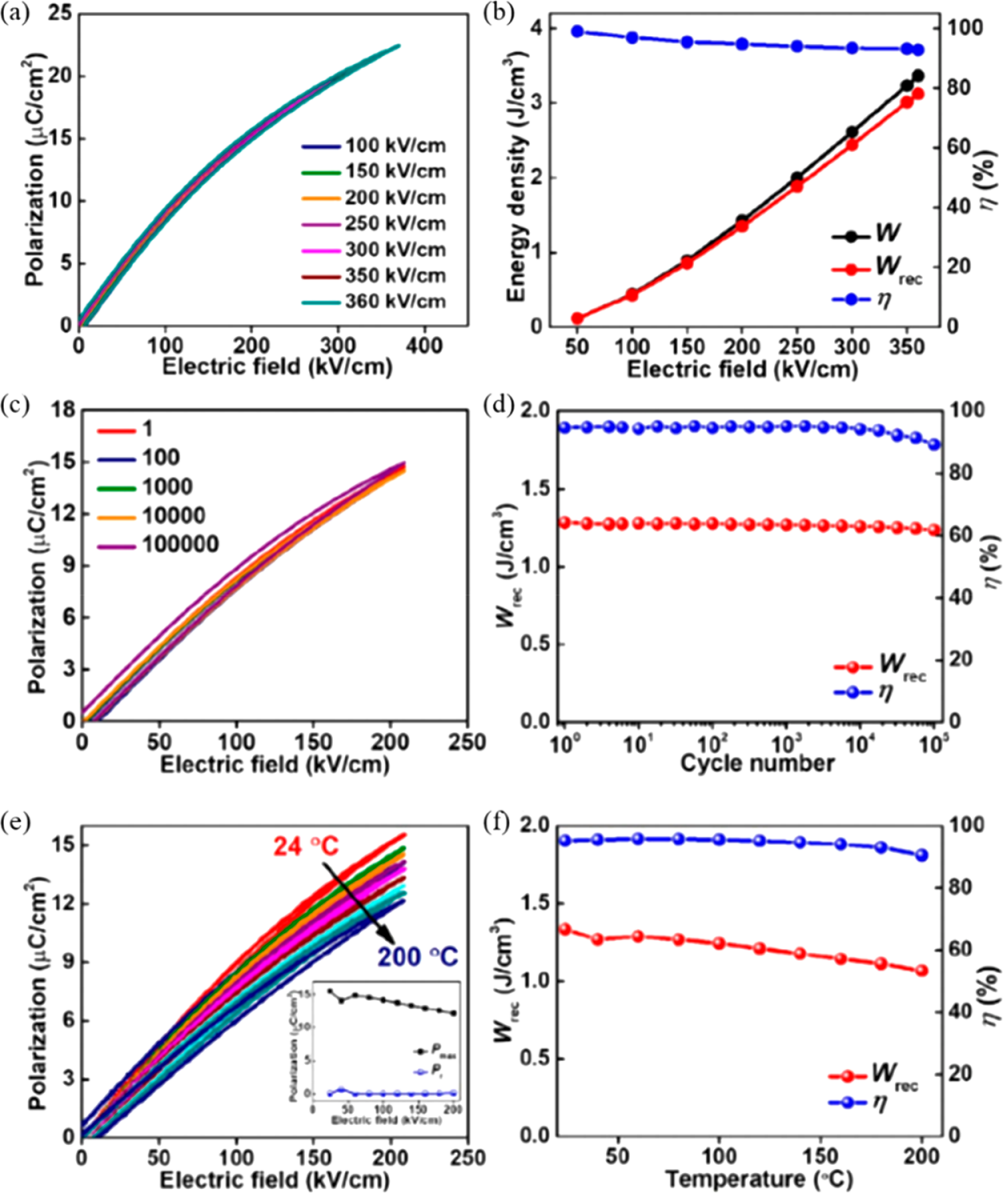
(a) Unipolar *P–E* loops and (b) *W*, *W*_rec_, and η of 0.9(Sr_0.7_Bi_0.2_)TiO_3_–0.1Bi(Mg_0.5_Hf_0.5_)O_3_ ceramic as functions of *E*. (c) Unipolar *P–E* loops and (d) *W*_rec_ and η of 0.9(Sr_0.7_Bi_0.2_)TiO_3_–0.1Bi(Mg_0.5_Hf_0.5_)O_3_ ceramic as functions of cycle numbers up to 10^5^. (e) Unipolar *P–E* loops, with the inset shows the *P*_max_ and *P*_r_ as functions of temperature, and (f) *W*_rec_ and η of 0.9(Sr_0.7_Bi_0.2_)TiO_3_–0.1Bi(Mg_0.5_Hf_0.5_)O_3_ ceramics as a function of temperature. Reproduced with permission from ref (242). Copyright 2019 John Wiley and Sons.

From a review of the literature, doping commonly increases both *ε*_r_ and *P*_max_ but decreases the BDS, sintering aids increase BDS but lower the *P*_max_. The highest energy densities have therefore been achieved through either dopants and/or alloying with “relaxor end-members” which also act as sintering aids. Adopting these protocols, a *W*_rec_ of 3.1 J cm^–3^ with η ∼ 93% was obtained for 0.9(Sr_0.7_Bi_0.2_)TiO_3_–0.1 Bi(Mg_0.5_Hf_0.5_)O_3_ ceramic at 360 kV cm^–1^, Figures 15a,b,^242^ which was also fatigue-resistant up to 10^5^ cycles and temperature stable from 25 to 200 °C, Figures 15c–f.^242^

##### 3.1.2.3 K_0.5_Na_0.5_NbO_3_-Based Ceramics

**Figure 16:**
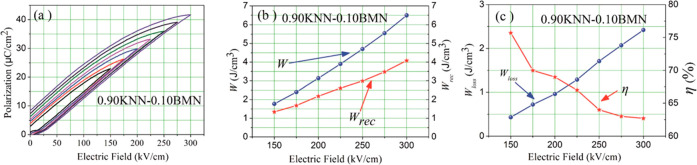
(a) Unipolar *P–E* hysteresis loops and (b) calculated *W* and *W*_rec_ and (c) *W*_loss_ and η for 0.90KNN–0.10BMN ceramics under different *E*. Reproduced with permission from ref (91). Copyright 2017 Royal Society of Chemistry.

In 2016, the energy storage properties of KNN–(Bi,Na)HfO_3_ solid solutions were first investigated, and *W*_rec_ ∼ 0.54 J cm^–3^ was achieved at 129 kV cm^–1^.^249^ ZnO and CuO were introduced as sintering aids improved *W*_rec_ in KNN-based ceramics^66,250^ by decreasing porosity and restricting grain growth. BDS of 400 kV cm^–1^ was obtained in 0.8KNN-0.2Sr(Sc_0.5_Nb_0.5_)O_3_ ceramic with 0.5 mol % ZnO^250^ and CuO reduced the sintering temperature and enhanced the density of 0.9KNN–0.1BMN ceramics.^66^ A third perovskite end-member, e.g., ST, BF, and BMN, has also been shown to optimize energy storage properties.^91,92,251^*W*_rec_ of 4.03 J cm^–3^ at 400 kV cm^–1^ was obtained in 0.85KNN–0.15ST bulk ceramics^92^ with similar energy storage performance realized in 0.90KNN–0.10BMN ceramic with a large *P*_max_ (41 μC cm^–2^) obtained at 300 kV cm^–1^, Figure 16.^91^ The energy storage properties of KNN-based materials are summarized in Table 6.

**Table 6:** Energy Storage Properties of KNN-Based Materials^a^

compounds	*E* (kV cm^–1^)	*ΔP* (μC cm^–2^)	*W*_rec_ (J cm^–3^)	η (%)	ref
(K_0.48_Na_0.52_)_0.88_Bi_0.04_NbO_3_	189	∼12.7	1.04		(252)
0.93KNN–0.07BMN	150	∼22.5	1.3	58.8	(253)
0.9KNN–0.1BF	206	23.7	2	61	(251)
0.8KNN–0.2Sr(Sc_0.5_Nb_0.5_)O_3_–0.5%ZnO	400	∼11.6	2.6	73.2	(250)
K_0.14_Bi_0.12_Na_0.5_NbO_3_–1 mol % CuO	300	29	2.89	80	(254)
0.85KNN-0.15 Bi(Zn_0.5_Zr_0.5_)O_3_	325	30	3.5	86.8	(255)
0.9KNN–0.1BMN–1.0 mol % CuO	400	∼21	4.02	57.3	(66)
0.85KNN–0.15ST	400	∼26	4.03	∼52	(92)
0.9KNN–0.1BMN	300	∼34	4.08	∼62.7	(91)

a *t* of the bulk ceramics is commonly >0.1 mm.

##### 3.1.2.4 BiFeO_3_-Based Ceramics

BF-based ceramics are best known as multiferroics but have also been explored for high-temperature ferroelectric and piezoelectric applications due to their high *T*_C_ and large spontaneous polarization.^256−259^ Compared with other lead-free ceramics, BF-based were not initially considered as good candidates for energy storage applications due to their high leakage current.^260^*p*-type electrical conductivity due to the volatilization of Bi and the variation of Fe valence states has been reported frequently in BF system, which limits the BDS and restricts energy density.^261−263^ However, the introduction of dopants and alloying with end-members suppresses the leakage current, reduces the electrical conductivity, increases intrinsic *E*_g_ and induces transitions from a FE to RFE state. The energy storage properties of BF-based materials are summarized in Table 7.

**Table 7:** Energy Storage Properties of BF-Based Materials^a^

compounds	*E* (kV cm^–1^)	*ΔP* (μC cm^–2^)	*W*_rec_ (J cm^–3^)	η (%)	ref
3 mol % Nb_2_O_5_–0.65BF–0.35BT	90	19	0.71		(265)
0.61BF–0.33BT–0.06Ba(Mg_2/3_Nb_1/3_)O_3_	125	32.3	1.56	75	(269)
0.61BF–0.33BT–0.06La(Mg_0.5_Ti_0.5_)O_3_	130	33.3	1.66	82	(267)
0.6BF–0.34BT–0.06Sr(Al_0.5_Nb_0.5_)O_3_	150	35	1.75	81	(270)
15%Nd–0.70BF–0.30BT	170	30.7	1.82	41.3	(90)
0.65BF-0.30BT–0.05Ba(Zr_0.5_Zn_0.5_)O_3_	190	32	2.04	54	(34)
0.65BF–0.30BT–0.05Bi(Zn_2/3_Nb_1/3_)O_3_	180	32.8	2.06	53	(266)
0.56BF–0.30BT–0.14AN	195	26	2.11	84	(268)
0.60BF–0.30BT–0.10Nd(Mg_2/3_Nb_1/3_)O_3_	220	24	2.4	77	(34)
0.7(0.67BF–0.34BT)–0.3(Sr_0.7_Bi_0.2_)TiO_3_	180	37	2.4	90.4	(271)
0.62BF–0.3BT–0.08Nd(Zr_0.5_Zn_0.5_)O_3_	240	26	2.45	72	(34)
0.6BF–0.34BT–0.06Ba(Zn_0.5_Ta_0.5_)O_3_	160	41	2.56	71	(272)
0.67Bi_0.9_Sm_0.1_FeO_3_–0.33BT	200	48	2.8	55.8	(273)
0.25Bi_0.83_Sm_0.17_Fe_0.95_Sc_0.05_O_3_−0.75[0.85BT-0.15Bi(Mg_0.5_Zr_0.5_)O_3_]	206	36	3.2	92	(274)
0.61BF–0.33BST–0.06La(Mg_2/3_Nb_1/3_)O_3_	230	36.5	3.38	59	(275)
0.57BF–0.30BT–0.13 Bi(Li_0.5_Nb_0.5_)O_3_	280	30	3.64	74	(276)
0.57BF–0.33BT–0.10NN	360	51	8.12	90	(42)
0.5BF–0.4ST–0.1BMN-0.03Nb	460	45	8.2	74.1	(45)

a The *t* of the bulk ceramics is commonly >0.1 mm.

BF–BT-based materials are purported as potential energy storage compositions due to their excellent BDS and high *P*_max_.^34,45,90^ Undoped BF–*x*BT ceramics exhibit optimized ferroelectric and piezoelectric properties in a mixed-phase region of 0.25≤ *x* ≤ 0.35.^264^ The majority of studies have focused on this region and modified compositions either by (i) A and/or B-site chemical doping, including Nd, Nb, Zn_0.5_Zr_0.5_, Zn_2/3_Nb_1/3_, and Li_0.5_Nb_0.5_ or (ii) alloying with a third end-member, such as Nd(Zn_0.5_Zr_0.5_)O_3_, Nd(Mg_2/3_Nb_1/3_)O_3_, La(Mg_0.5_Ti_0.5_)O_3_, Ba(Mg_1/3_Nb_2/3_)O_3_, AN, and NN.^34,43,90,265−269^

**Figure 17:**
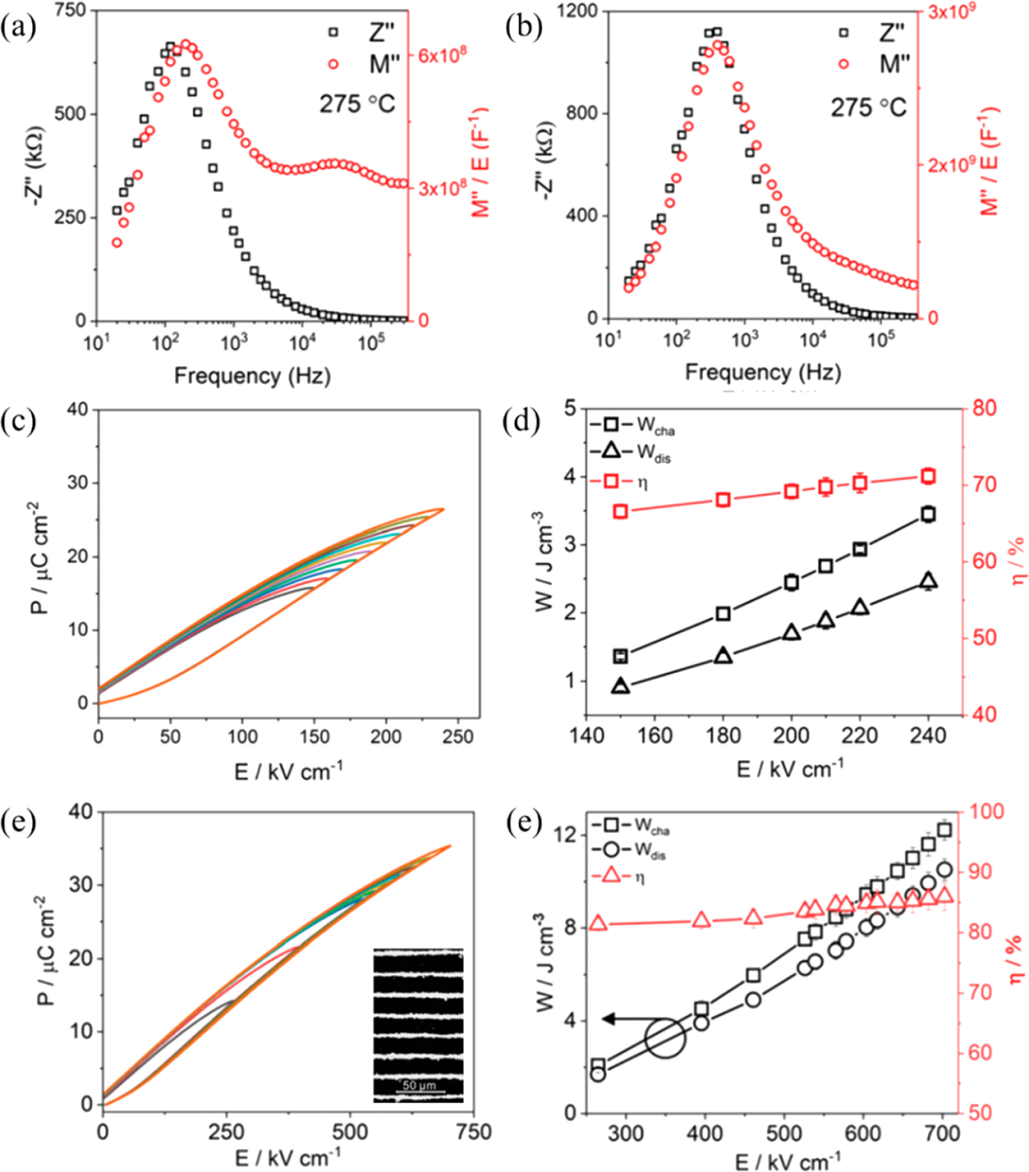
Combined *Z′′* and *M′′* spectroscopic plots at 275 °C for (a) BF–BT and (b) BF–BT–0.08NZZ. Unipolar *P–E* loops of BF–BT–0.08NZZ (c) bulk ceramics and (e) ceramic MLs, with cross-section SEM image as shown in inset figure. Calculated energy storage properties of BF–BT–0.08NZZ (d) bulk ceramic and (e) ceramic MLs. Reproduced from ref (34). Copyright 2019 Royal Society of Chemistry.

The importance of electrical homogeneity was first postulated in 2019 by Wang, Reaney, and co-workers as a key factor to optimize BDS as well as *W*_rec_.^34^ Two electrical components with similar capacitance value, *C*∼ 8 × 10^–10^ F cm^–1^ and 1.4 × 10^–9^ F cm^–1^, were found in 0.7BF–0.3BT ceramics, as illustrated Figure 17a,b. However, the associated resistances, *R*_1_ ∼ 3.8 kΩ cm and *R*_2_ ∼ 1.3 MΩ cm, were vastly different. The presence of a large volume fraction of conductive cores (*R*_1_) easily led to electrical breakdown at lower electrical field. In contrast, only one single electrical component with *C* ∼ 1.87 × 10^–9^ F cm^–1^ and resistivity of ∼2.3 MΩ cm was observed for 8% Nd(Zr_0.5_Zn_0.5_)O_3_ (NZZ)-doped BF–BT ceramics (Figures 17c,d). The conductive electrical component was effectively eliminated by forming a solid solution with NZZ, inhibiting the formation of conductive pathways at higher electric field (>500 kV cm^–1^). As a result, optimized *W*_rec_ ∼ 2.45 and 10.5 J cm^–3^ were realized at *E*_max_ ∼ 240 and 700 kV cm^–1^ for 0.08NZZ-BF–BT bulk ceramics and MLs, respectively.

**Figure 18:**
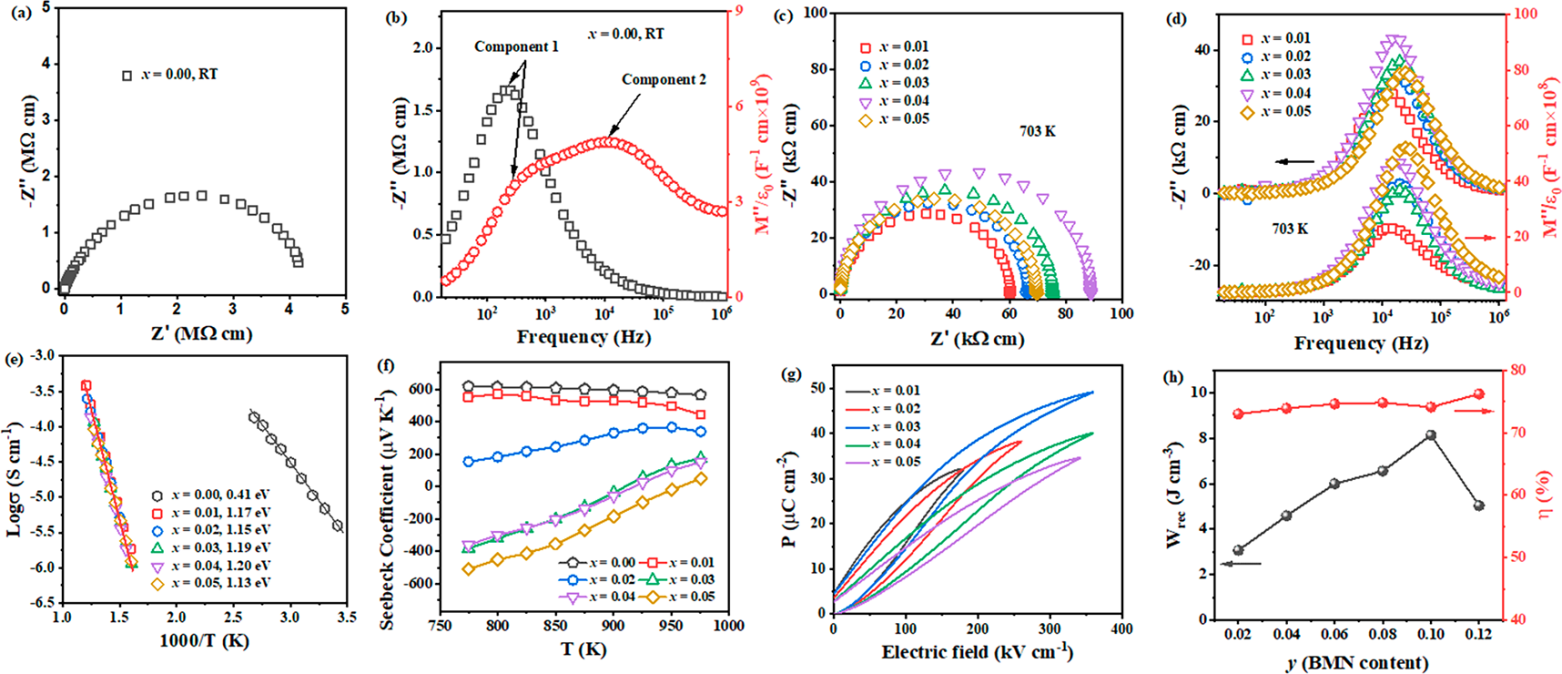
(a) Z* plots and (b) Combined *Z′′* and *M′′* spectroscopic plots of 0.54BF–0.4ST–0.06BMN–*x*Nb (*x* = 0); (c) Z* plots and (d) Combined *Z′′* and *M′′* spectroscopic plots of *x* = 0.01–0.05; (e) Arrhenius plots, (f) Seebeck coefficients, and (g) unipolar *P–E* loops under *E*_max_ of *x* = 0.01–0.05. (h) *W*_rec_ and η of (0.6–*y*)BF–0.4ST–0.03Nb–*y*BMN. Reproduced from ref (45). Copyright 2020 Royal Society of Chemistry.

Recently, superior energy density through tailored dopant strategies was achieved in BF–ST–*x*Nb–*y*BMN ceramics, by promoting electrical homogeneity, enhancing *E*_a_ and suppressing the *p*-type conduction, all of which resulted in significantly enhanced BDS. The ceramic without Nb (BF–ST–0.06BMN, *x* = 0) exhibits a broadened arc in Z* at room temperature (Figure 18a) with at least two electrical components observed in combined *Z′′* and *M*′′** spectroscopic plots (Figure 18b). By donor doping Nb on the B-site, only one ideal semicircle in the Z* plots with a single Debye peak at the same frequency in both *Z′′* and *M*′′** spectroscopic plots was observed for all doped samples (Figures 18c,d), suggesting electrical homogeneity. Nb doping suppresses the formation of Fe^4+^ associated with the loss of Bi_2_O_3_ or/and *V*_*O*_^..^ during ceramic processing, thus reducing electrical conductivity with respect to *x* = 0 by several orders of magnitude, coupled with enhanced *E*_a_ (Figure 18e). A reduction in Seebeck coefficient from ∼ 600 μV K^–1^ to zero indicates a commensurate decrease in charge carrier concentration as Nb concentration increases. At *x* = 0.03, the BDS increases to 360 kV cm^–1^ (Figures 18g) which is insufficient on its own to optimize *W*_rec_ but when combined with an increase in BMN (*y*) concentration to reduce polar coupling, results in *W*_rec_ ∼ 8.2 J cm^–3^ at *E*_max_ ∼ 460 kV cm^–1^ for BF–ST–0.03Nb–0.1BMN ceramics, Figure 18h.^45^

##### 3.1.2.5 Na_0.5_Bi_0.5_TiO_3_–Based Ceramics

NBT-based ceramics are promising candidates of lead-free dielectrics due to their high *P*_max_ and *T*_c_. However, their large hysteresis and low BDS are not ideal for high energy density capacitor applications.^277−284^ Attempts to improve their properties generally fall into the following approaches: (i) doping on the A-site (Ba, Sr, K, Li, La, Dy, Nd)^222,239,243,285−296^ and B-site (commonly Nb)^297^ and codoping (K,Sr/Nb; K,La/Zr; Li,K,Sr/Ta,Nb; K,Mg/Nb; Ba/Nb; Ba/Sn; Ba/Sn,Zr; Ba/Ta; Ba/Zr; Ba,Ca/Zr; Ba,K,Ca/Nb,Zr; Ba/Mg,Nb; Ba/Mg; Ba,La/Al,Nb; Ba,Sr/Yb,Nb; Ba/Hf; La/Al; La,Ba/Nb; Sr/Sn; Sr/Zr; Sr/Mg; Sr/Mg,Nb);^194,298−321^ (ii) forming solid solution with other end-members, such as AN, NN, and SBT;^297,322−325^ (iii) using additives such as MnO, Fe_2_O_3_, MgO, SnO_2_, ZnO, CaO, and ZrO_2_^62,74,238,326−334^ and (iv) employing different processing methods such as hot-pressing^59^ and synthesis using sol–gel derived powders.^69,71,74,335,336^ The energy storage properties of NBT-based materials are summarized in Table 8.

**Table 8:** Energy Storage Properties of NBT-Based Materials^a^

compounds	*E* (kV cm^–1^)	*ΔP* (μC cm^–2^)	*W*_rec_ (J cm^–3^)	η (%)	ref
0.95(0.76NBT–0.24ST)-0.05AN	120	43.5	2.03	61.8	(324)
Bi_0.41_Na_0.35_Sr_0.21_TiO_3_	135	∼35.8	2.04	82.4	(290)
0.75Na_0.25_Sr_0.5_Bi_0.25_TiO_3_–0.25MgO	200	∼35	2.06	84	(317)
0.8(0.775NBT–0.225BaSnO_3_)–0.2BaZrO_3_	245	20	2.08	88.8	(315)
0.85NBT–0.15BaHfO_3_	175	29	2.1	66.1	(310)
0.775NBT–0.225BaSnO_3_–5 wt %MgO	215	∼28	2.13	67.8	(326)
Sn–0.45ST–0.2NBT–0.35BT	240	∼21	2.25	79.51	(328)
0.9NBT–0.1Li_2_TiO_3_	200	∼25	2.3	74.2	(291)
(0.5NBT–0.5ST) −1%SnO_2_	180	37.37	2.35	∼80	(238)
(Na_0.5_Bi_0.5_)_0.8_Ba_0.2_Ti_0.8_Sn_0.8_O_3_	195	∼34	2.35	71.04	(338)
{Bi_0.5_[(Na_0.8_K_0.2_)_0.9_Li_0.1_]_0.5_}_0.96_Sr_0.04_(Ti_0.975_Ta_0.025_)O_3_	143	∼43	2.42	∼64	(339)
0.55NBT–0.45(Bi_0.2_Sr_0.7_TiO_3_)	200	25	2.5	95	(222)
0.94(BNT–Bi_0.2_Sr_0.7_TiO_3_)–0.06KNN	180	37	2.65	84.6	(298)
0.95(0.6ST–0.4NBT)–0.05Zr	285	∼25	2.84	71.54	(74)
0.85(0.95NBT–0.05SrZrO_3_)–0.15NN	210	∼30	2.93	72	(325)
0.96(0.65NBT–0.35Sr_0.85_Bi_0.1_TiO_3_)–0.04NN	220	50.46	3.08	81.4	(323)
0.6(Bi_0.51_Na_0.47_)TiO_3_–0.4Ba(Zr_0.3_Ti_0.7_)O_3_	280	∼25	3.1	91	(299)
0.9(0.76NBT−0.24NN)−0.1SBT	200	43	3.12	75	(340)
0.93NBT–0.07LaAlO_3_	210	43	3.18	60	(320)
(Na_0.25_Bi_0.25_Sr_0.5_)(Ti_0.8_Sn_0.2_)O_3_	310	26.5	3.4	90	(321)
0.85(Na_0.5_Bi_0.5_)_0.7_Sr_0.3_TiO_3_−0.15BMN	250	38	3.45	88.01	(316)
0.95(0.6 Bi_0.5_Na_0.5_TiO_3_–0.4Sr_0.7_Bi_0.2_TiO_3_)–0.05AN	246	41	3.62	89	(322)
0.65(0.84NBT–0.16K_0.5_Bi_0.5_TiO_3_)–0.35(Bi_0.2_Sr_0.7_TiO_3_)	350	∼33.99	4.06	87.3	(288)
0.55NBT–0.45SBT	315	19.1	4.14	92.2	(295)
0.90(Na_0.5_Bi_0.5_)_0.7_Sr_0.3_TiO_3_–0.10 Bi(Ni_0.5_Sn_0.5_)O_3_	270	47	4.18	83.64	(341)
0.75Bi_0.58_Na_0.42_TiO_3_–0.25ST	535	41	5.63	94	(342)
0.78NBT–0.22NN	390	45	7.02	85	(297)
0.62NBT–0.30SBT–0.08BMN	470	48	7.5	92	(337)

a *t* of the bulk ceramics is commonly >0.1 mm.

**Figure 19:**
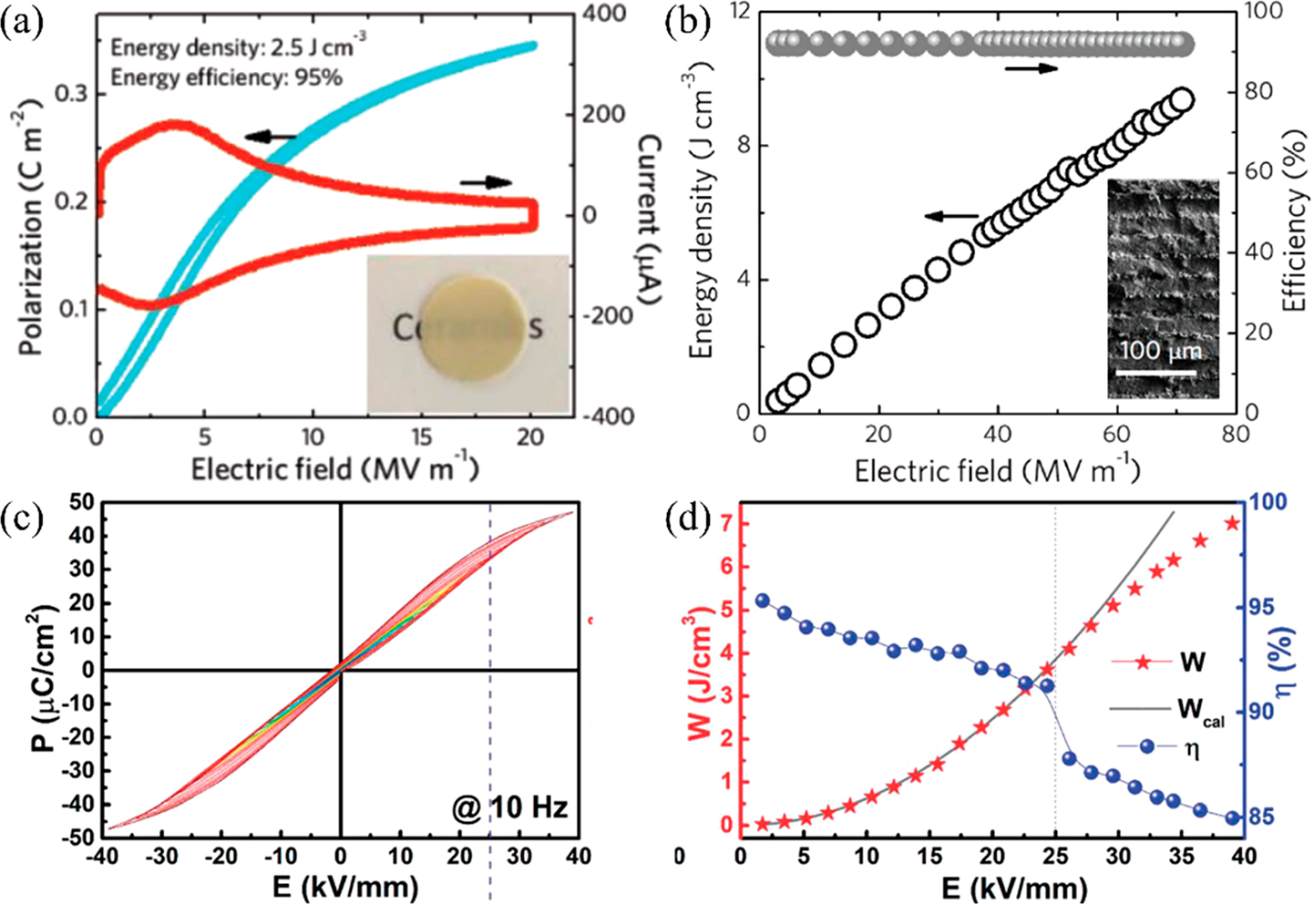
(a) Unipolar *P–E* loop and the current–*E* curve for NBT–0.45SBT bulk ceramics. (b) *W*_rec_ and η for NBT–0.45SBT ceramic MLs; inset SEM image of the ceramic MLs (c) Bipolar *P–E* loops and (c) calculated *W*_rec_ and η of 0.78NBT–0.22NN ceramic.^222,297^ (a, b) Reproduced with permission from ref (222). Copyright 2018 John Wiley and Sons; (c, d) Reproduced with permission from ref (297). Copyright 2019 Royal Society of Chemistry.

Notably, Li and co-workers reported that 0.55NBT–0.45(Sr_0.7_Bi_0.2_)TiO_3_(SBT) achieved *W*_rec_ of 2.5 and 9.5 J cm^–3^ with η > 90% for bulk ceramic and MLs at 200 and 720 kV cm^–1^ (Figure 19a,b), respectively.^222^ Superior *W*_rec_ ∼ 7.02 J cm^–3^ and η ∼ 85%, were also reported for 0.78NBT–0.22NN ceramics at *E*_max_ ∼ 360 kV cm^–1^, Figures 19c,d, with <10% variation from 25–250 °C and from 0.1 to 100 Hz.^297^

**Figure 20:**
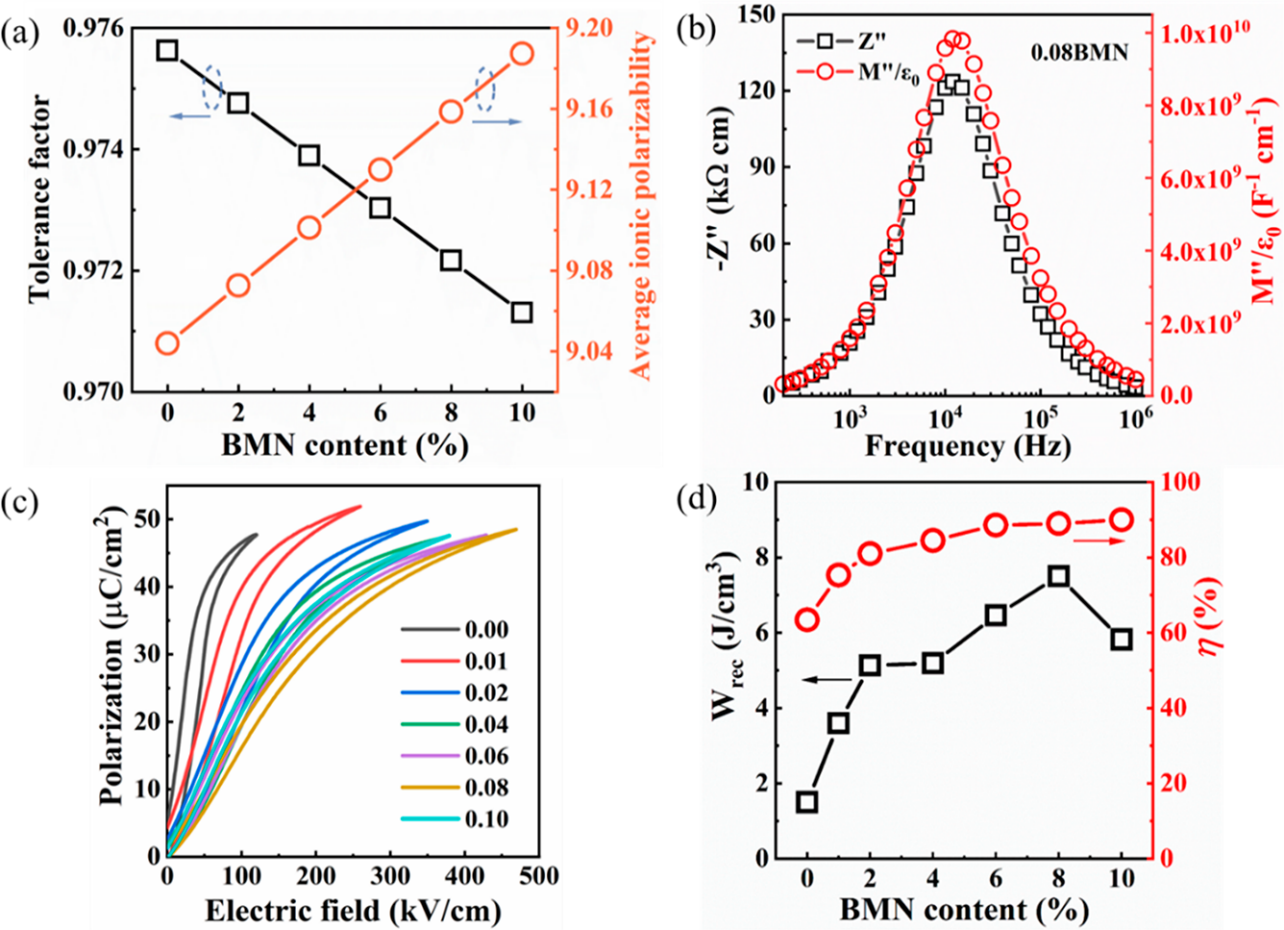
(a) Tolerance factor and average ionic polarizability per unit cell of NBT–SBT–*x*BMN as a function of BMN concentration. (b) Combined *Z*′′ and *M*′′ spectroscopic plots for NBT–SBT–0.08BMN ceramics at 660 °C. (c) *P–E* loops at the *E*_max_, and (d) *W*_rec_ and η for NBT–SBT–*x*BMN ceramics. Reproduced with permission from ref (337). Copyright 2021 Elsevier.

Recently, Ji and co-workers^337^ proposed that the key factors for designing an ideal RFE with high energy density were as follows: (i) utilization of a highly polar base system (e.g., NBT); (ii) disruption of long-range polar coupling through forming solid solutions with, e.g., SBT and BMN without sacrificing average ionic polarizability, Figure 20a, and (iii) simultaneously inducing or retaining electrical homogeneity with a highly resistive single component in IS (∼250 kΩ cm at 660 °C), Figure 20b. These factors combined to give *E*_max_ ∼ 470 kV cm^–1^, *W*_rec_ ∼ 7.5 J cm^–3^, and η ∼ 92% for 0.62NBT–0.30SBT–0.08BMN, Figure 20c,d.

##### 3.1.2.6 AgNbO_3_–Based Ceramics

AFEs have long been considered as the prime candidate for energy storage capacitors due to their large *P*_max_ and small *P*_*r*_. There are only a handful of lead-free AFE systems, with AN showing particular promise because it possesses a large saturation polarization of 52 μC cm^–2^ under an *E*_max_ ∼ 220 kV cm^–1^.^343^ Recent research on AN ceramics has focused on stabilizing the AFE phase so that switching field is moved to higher fields while simultaneously optimizing *P*_max_.^344,345^

**Figure 21:**
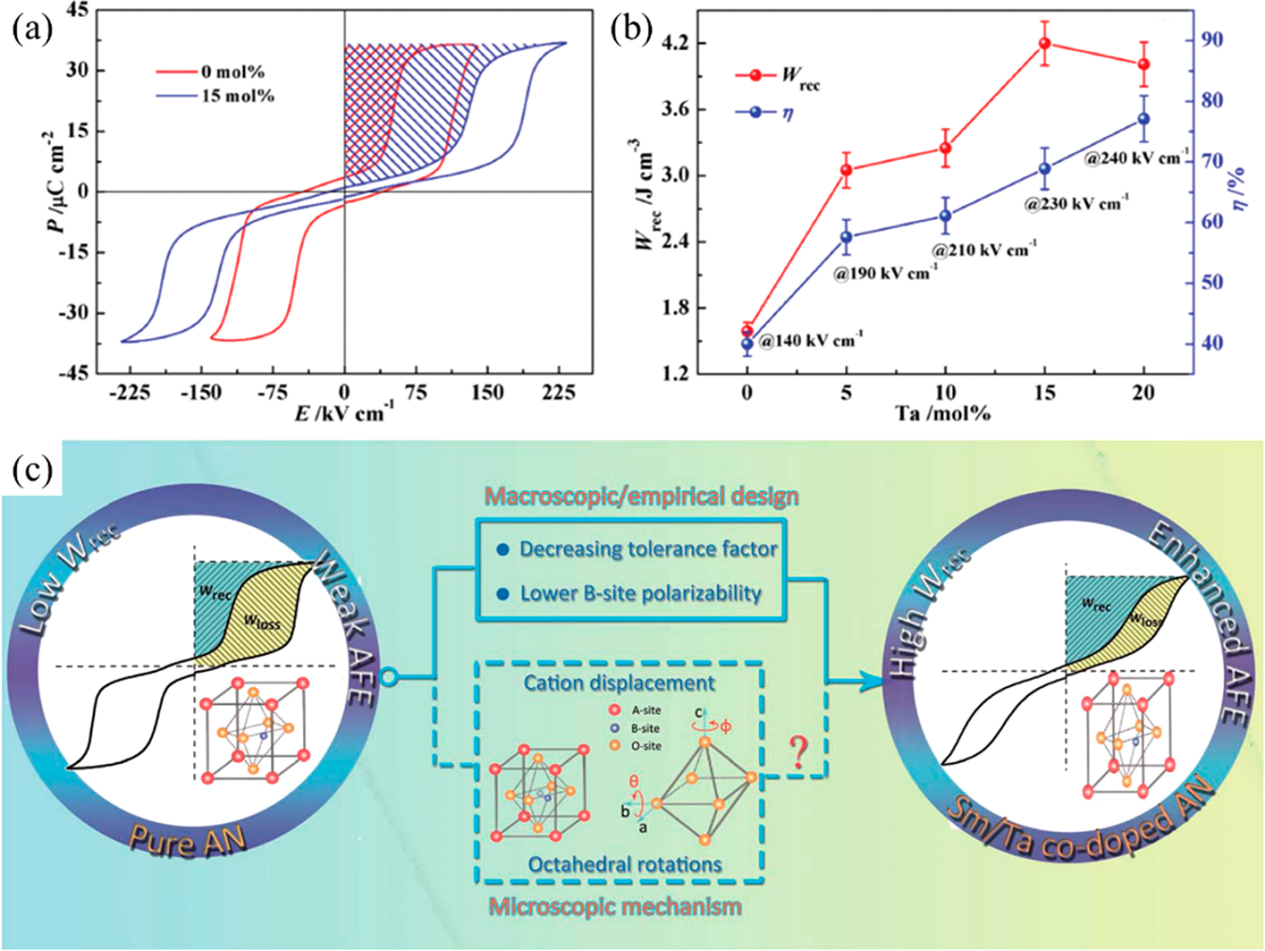
(a) Bipolar *P–E* loops of AN and Ag(Nb_0.85_Ta_0.15_)O_3_ ceramics. (b) Energy storage performance of Ag(Nb_1–*x*_Ta_*x*_)O_3_ ceramics prior to their breakdown. (c) Schematic of the underlying principles for enhancing energy storage property in AN-based materials. (a, b) Reproduced with permission from ref (357). Copyright 2017 John Wiley and Sons; (c) Reproduced with permission from ref (359). Copyright 2019 Royal Society of Chemistry.

There have been a number of recent studies on AN focusing on (i) substitution of aliovalent B-site oxides such as MnO_2_ and WO_3_;^346,347^ (ii) doping Ba, Sr, Ca, Bi, La, Sm, and Gd on the A-site^348−356^ often with isovalent Ta doping on the B-site;^357−359^ and (iii) forming solid solutions with end-members, such as BiMnO_3_ and Bi(Zn_2/3_Nb_1/3_)O_3_.^360,361^ Most dopants reduce the *G* of AN which maximizes BDS but delay the onset of the AFE-FE transition to higher field while simultaneously narrowing hysteresis in the induced FE phase. *W*_rec_ of 4.4, 4.5, and 5.2 J cm^–3^ with η of 70, 63, and 69.2% has been obtained for La, Gd, and Sm A-site doped AN ceramics,^348,351,355,359,362^ respectively, and B-site Ta-doped AN was reported to exhibit *W*_rec_ of 4.2 J cm^–3^ with η of 69% (Figure 21a,b).^357^ A-site doping with ions smaller in radius than Ag is suggested to decrease tolerance factor and enhance AFE stability, while donor doping is compensated by A-site vacancies which reduce antipolar and polar coupling of the AFE and field induced FE phases, respectively. Some authors postulate that substituting B-site ions with a lower polarizability than Nb also stabilizes the AFE phase and moves the switching field higher.^348,350,359,363^ The underlying principles are schematically represented in Figure 21c. The energy storage properties of AN-based materials are summarized in Table 9.

**Table 9:** Energy Storage Properties of AN-Based Materials^a^

compounds	*E* (kV cm^–1^)	*ΔP* (μC cm^–2^)	*W*_rec_ (J cm^–3^)	η (%)	ref
AN	150	∼34	2.0	46	(345)
AN	175	∼33	2.1	40–50	(343)
Ag_0.96_Ba_0.02_NbO_3_	∼180	∼34	2.3	46	(356)
0.6 mol % BiMnO_3_–AN	175	∼36	2.4	54	(360)
0.1 wt % Mn–AN	150	∼37.2	2.5	57	(346)
Ag_0.91_Bi_0.03_NbO_3_	200	∼30	2.6	86	(353)
Ag_0.90_Sr_0.05_NbO_3_	190	∼38	2.9	56	(354)
Ag_0.94_La_0.02_NbO_3_	230	∼28	3.12	63	(352)
0.3 wt % Mn-doped Ag_0.97_La_0.01_NbO_3_	142	37.8	3.2	62	(351)
0.1 wt % W-AN	200	∼42.5	3.3	50	(347)
AN–0.03NBT	220	33	3.4	62	(364)
Ag_0.90_Ca_0.05_Nb_0.95_Ta_0.05_O_3_	210	37	3.36	58	(363)
Ag_0.92_Ca_0.04_NbO_3_	220	∼37.6	3.55	63	(349)
Ag(Nb_0.8_Ta_0.2_)O_3_	270	∼30	3.7	∼65	(358)
AgNb_0.85_Ta_0.15_O_3_	233	∼35.1	4.2	69	(357)
2 mol % La-doped AN	273	∼30	4.4	70	(350)
Ag_0.88_Gd_0.04_NbO_3_	290	∼32.5	4.5	∼63	(355)
Ag_0.94_Sm_0.02_NbO_3_	310	31	4.5	63	(362)
0.99AN–0.01 Bi(Zn_2/3_Nb_1/3_)O_3_	220	46.8	4.6	57.5	(115)
(Sm_0.02_Ag_0.94_)(Nb_0.9_Ta_0.1_)O_3_	280	∼36	4.87	63.5	(359)
Sm_0.03_Ag_0.91_NbO_3_	290	∼36	5.2	69.2	(348)
AgNb_0.45_Ta_0.55_O_3_	460	∼29	6.3	90	(365)
Ag_0.97_Nd_0.01_Nb_0.80_Ta_0.20_O_3_	370	38	6.5	71	(366)
Ag_0.76_La_0.08_NbO_3_	476	33	7.01	77	(367)

a *t* of the bulk ceramics is typically >0.1 mm.

##### 3.1.2.7 NaNbO_3_-Based Ceramics

Recently, AFE NN has received attention as a potential candidate for energy storage applications. An AFE double hysteresis loop is difficult to observe in NN because (i) the energy difference between the AFE phase and field-induced FE phase is very small and (ii) *E*-induced FE phase is metastable. Thus, AFE behavior in NN based materials is commonly stabilized by chemical substitution with end members such as BS and CaHfO_3_.^344,368−370^

**Figure 22:**
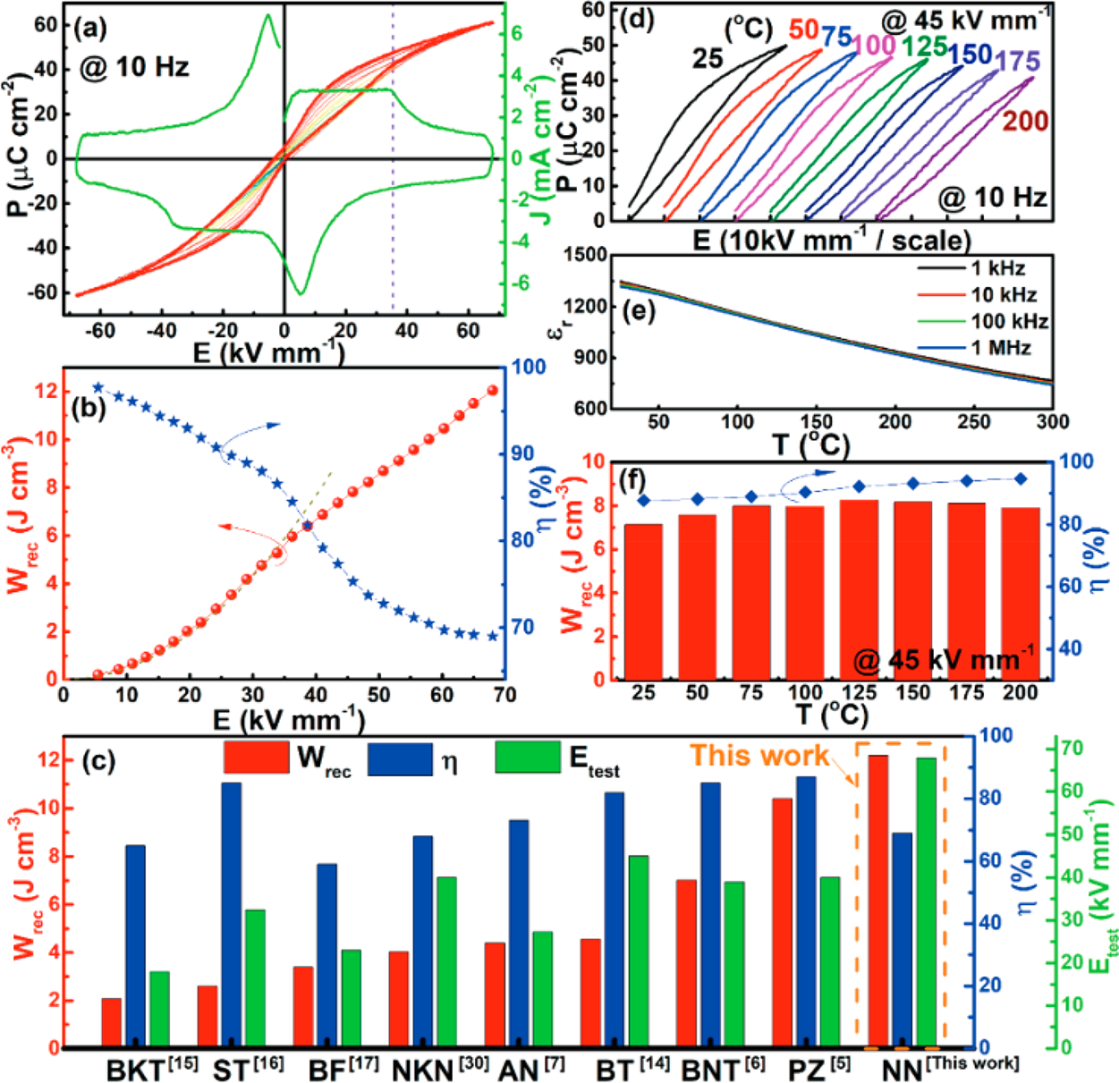
(a) Bipolar *P–E* loops with corresponding current density-field (*J–E*) curves and (b) *W*_rec_ and η values of under different *E* for the 0.76NN–0.24BNT ceramic at 10 Hz (c) a comparison of *W*_rec_, η, and *E*_max_ among the recently reported bulk ceramics; (d) temperature-dependent *P–E* hysteresis, (e) temperature- and frequency-dependent *ε*_r_ and (f) *W*_rec_ and η as a function of temperature for the 0.76NN-0.24NBT ceramic at 450 kV cm^–1^. Reproduced with permission from ref (42). Copyright 2019 John Wiley and Sons.

*E*_max_ > 250 kV cm^–1^ and *W*_rec_ > 2.5 J cm^–3^ have been reported for NN in solid solution with BMN, ST, Bi(Mg_2/3_Ta_1/3_)O_3_, and Bi(Mg_0.5_Ti_0.5_)O_3_ by stabilizing the AFE phase or inducing relaxor behavior,^371−374^ as summarized in Table 10. Zuo and co-workers^42^ have also proposed the concept of an “AFE relaxor” to explain the energy storage properties of 0.78NN–0.22NBT ceramics. They argue that the local AFE phase transforms reversibly into an FE monoclinic phase at∼ 400 kV cm^–1^, giving a large *ΔP* (*P*_max_ > 50 μC cm^–2^ and *P*_rem_ < 5 μC cm^–2^). *W*_rec_ of ∼12.2 J cm^–3^ was reported with η ∼ 69%, at 680 kV cm^–1^, Figure 22.^42^ However, the term “AFE relaxor” has little physical significance since an antipolar phase cannot form short-range polar features characteristic of a relaxor. 0.78NN–0.22NBT may, therefore, be better described as either a relaxor or a short-range AFE phase that undergoes a field induced transition. This intriguing behavior is interesting, but it is the large *E*_max_ (680 kVcm^–1^) that is most likely responsible for the exceptional *W*_rec_ rather than the intrinsic crystal chemistry. The underpinning reasons for the large *E*_max_ most likely relate to the defect chemistry, band gap and electrical homogeneity, consistent with the key factors proposed by Ji and co-workers.^337^

**Table 10:** Energy Storage Properties of NN-Based Materials^a^

compounds	*E* (kV cm^–1^)	*ΔP* (μC cm^–2^)	*W*_rec_ (J cm^–3^)	η (%)	ref
NN–0.09Bi(Zn_0.5_Ti_0.5_)O_3_	200	29	2.1	76	(375)
0.9NN–0.10BMN	300	∼23	2.8	82	(371)
0.8NN–0.2ST	323	34.5	3.02	80.7	(372)
0.78NN–0.22Ba(Mg_2/3_Nb_1/3_)O_3_	540	18.7	3.51	87	(376)
Na_0.7_Bi_0.1_NbO_3_	250	∼30	4.03	85.4	(377)
NN–MnO_2_	360	33	4.3	90	(378)
Na_0.84_Bi_0.08_Nb_0.92_Zr_0.08_O_3_	430	30	4.9	88	(67)
0.9NN–0.1 Bi(Ni_0.5_Sn_0.5_)O_3_	550	25	5	68	(379)
0.78NN–0.22 Bi(Mg_2/3_Ta_1/3_)O_3_	620	17	5.01	86.8	(373)
0.92NN–0.08 Bi(Mg_0.5_Ti_0.5_)O_3_	480	38	5.57	71	(374)
(Na_0.91_La_0.09_)(Nb_0.82_Ti_0.18_)O_3_	550	42	6.5	65.9	(380)
0.75[0.9NN–0.1 Bi(Mg_0.5_Ta_0.5_)O_3_]–0.25(Bi_0.5_Na_0.5_)_0.7_Sr_0.3_TiO_3_	800	22	8	90.4	(381)
0.76NN–0.24NBT	680	∼55	12.2	69	(42)

a *t* of the bulk ceramics is commonly >0.1 mm.

#### 3.1.3 Glass Ceramics

**Figure 23:**
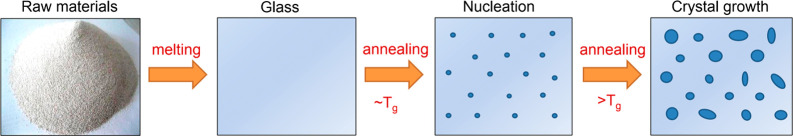
Schematic of the processing step of glass-ceramics.

Glass-ceramics are composed of one or more crystallized phases (ceramics) dispersed uniformly in amorphous phase (glass). They often exhibit the combined properties of ceramics and glass depending on the induced crystalline phases and their microstructures. Glass-ceramics are prepared by melting the requisite raw materials, cooling to room temperature to form a glass, followed by two step annealing to induce crystal nucleation (approximately at the glass transition temperature, *T*_g_) and growth > *T*_g_Figure 23.^179,382^ The microstructure of a glass ceramic is typically dominated by a largely 2D and 3D defect-free (e.g., no grain boundaries) glass phase and a uniformly distributed (provided the system undergoes homogeneous rather than heterogeneous nucleation) ceramic phase. *W*_rec_ and η are both large due to the high BDS associated with the absence of 2D and 3D defects accompanied by a near zero value of *P*_*r*_. The energy storage properties of glass-based glass ceramics are summarized in Table 11.

**Table 11:** Energy Storage Properties of Glass Ceramics^a^

compounds	*ε*_r_ (1 kHz, 300 K)	*E* (kV cm^–1^)	*W*_rec_ (J cm^–3^)	ref
14.4SrO–17.6BaO–32Nb_2_O_5_–36B_2_O_3_	117	1050	5.71	(386)
25.6BaO–6.4Na_2_O–32Nb_2_O_5_–36SiO_2_	∼90	1248	∼6.2	(409)
14.3SrO–17.5BaO–31.9Nb_2_O_5_–35.8B_2_O_3_–0.5ZnO + 0.5La_2_O_3_	131	1127	7.1	(400)
20BaO–20SrO–20Nb_2_O_5_-5Al_2_O_3_–1.5B_2_O_3_–33.5SiO_2_ + 0.2La_2_O_3_	92.4	1326	7.2	(406)
20SrO–20BaO–10Nb_2_O_5_–10TiO_2_–32SiO_2_–8Al_2_O_3_	52.9	1817	7.73	(410)
14.3SrO–17.5BaO– 31.9%Nb_2_O_5_–35.8%B_2_O_3_–0.5ZnO + 0.5Sm_2_O_3_	143.8	1132	8.15	(402)
14.4SrO–17.6BaO–32Nb_2_O_5_–36B_2_O_3_ + 1%Yb_2_O_3_	98.3	1398	8.5	(403)
15K_2_CO3–15SrCO3–30Nb_2_O_5_–32SiO_2_–4Al_2_O_3_–4B_2_O_3_	102 (10 kHz)	1411	8.99	(407)
20BaO–20SrO–20Nb_2_O_5_–5Al_2_O_3_–1.5B_2_O_3_–33.5SiO_2_ + 0.05MnO_2_	95.8	1471	9.2	(411)
42[0.2Na_2_O–0.8SrO]–28Nb_2_O_5_–30SiO_2_	53 (100 kHz)	2074	10.09	(412)
9.6K_2_O–22.4BaO–32Nb_2_O_5_–36SiO_2_	75	1937	12.06	(393)
25.6BaO–6.4K_2_O–32Nb_2_O_5_–36SiO_2_ + 1Gd_2_O_3_	83	1818	12.14	(405)
20BaO–12K_2_O–32Nb_2_O_5_–36SiO_2_	83	1859	12.7	(413)
15.16SrO–6.736BaO–10.104K_2_O–32Nb_2_O_5_–28B_2_O_3_–8P_2_O_5_	85.2	1844	12.83	(395)
6.4K_2_O–25.6SrO–32Nb_2_O_5_–36SiO_2_ + 3CaF_2_	114	1623	13.5	(414)
31.2SrO–7.8Na_2_O–26Nb_2_O_5_–35SiO_2_	91	1941	15.2	(390)
15 Bi_2_O_3_–15Nb_2_O_5_–40SiO_2_–30Al_2_O_3_	100	1861	15.3	(391)
65(48SrO–12Na_2_O–40Nb_2_O_5_)–35SiO_2_	124	1669	15.3	(392)
24BaO–6Na_2_O–30Nb_2_O_5_–10Al_2_O_3_–30SiO_2_	∼70	2322	16.6	(388)
15.4Na_2_O–15.4PbO–23.1Nb_2_O_5_–46.2SiO_2_	175	1486	17	(385)
25.6(0.4SrO–0.6BaO)–6.4K_2_O–32Nb_2_O_5_–36SiO_2_	118	1828	17.45	(394)
21.25BaO–1PbO–12.75Na_2_O–34Nb_2_O_5_–32SiO_2_	154	1638	18.29	(389)
25.6BaO–3.2Na_2_O–3.2K_2_O–32Nb_2_O_5_–36SiO_2_	22	4433	19	(396)
21.6BaO–2.4PbO–6Na_2_O–30Nb_2_O_5_–10Al_2_O_3_–30SiO_2_	137	1848	20.7	(384)
63SiO_2_–12BaO–16B_2_O_3_–9Al_2_O_3_	6	12,000	38.5	(397)

a *t* of the bulk ceramics is commonly >0.1 mm.

**Figure 24:**
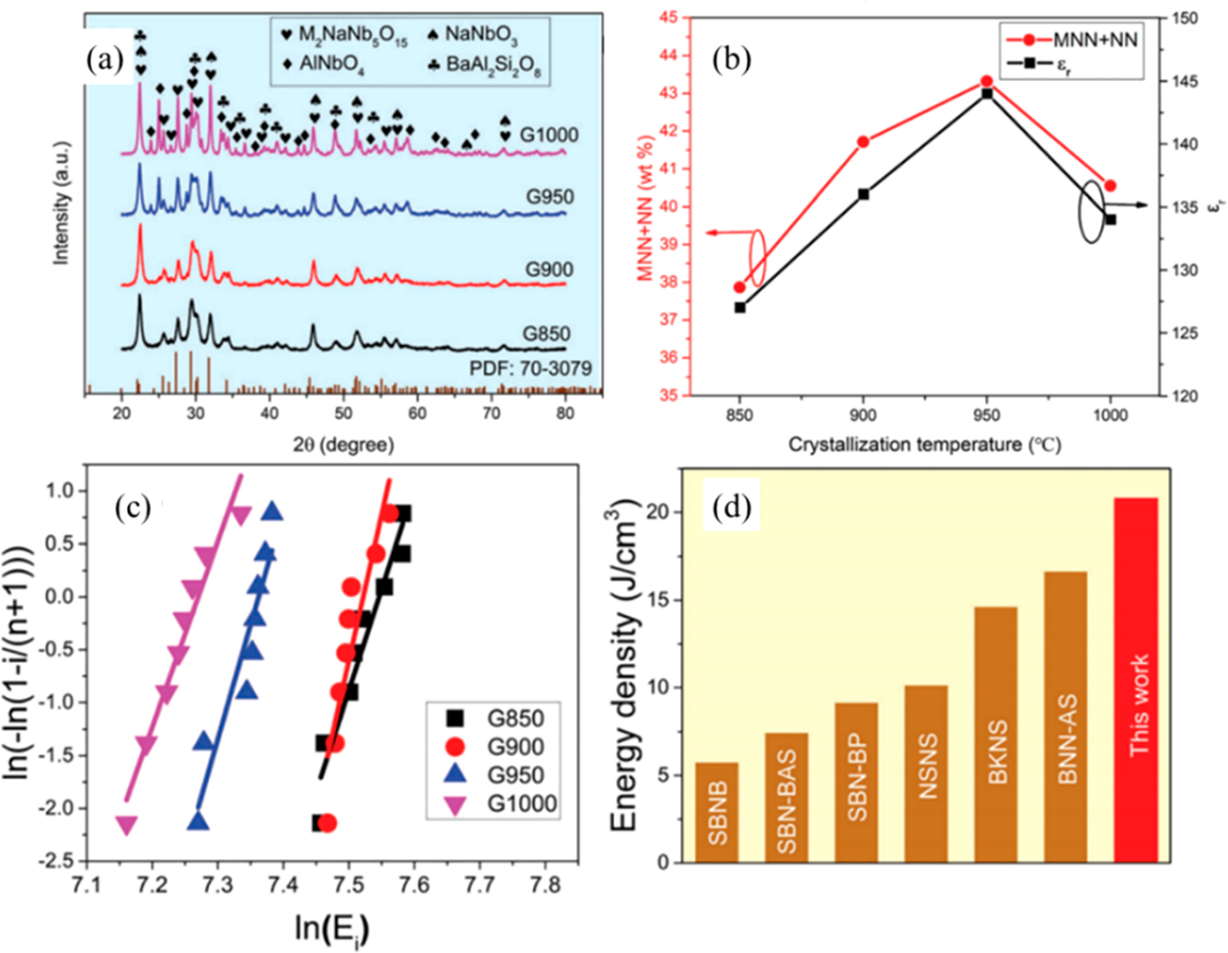
(a) X-ray diffraction (XRD) patterns of BPNN-AS glass ceramics annealed from 850 to 1000 °C; (b) *ε*_r_ and M_2_NaNb_5_O_15_ + NN phase proportion with increasing annealing temperature; (c) BDS Weibull distribution plots; (d) *W*_rec_ of 900 °C annealed BPNN-AS glass ceramics, compared with other kinds of ferroelectric glass ceramics. Reproduced with permission from ref (384). Copyright 2018 Royal Society of Chemistry.

As discussed above, the crystallization of glass ceramics is controlled by the annealing procedure, where the annealing temperature and time are critical for nucleation and growth of the ceramic phase, the microstructure and the properties. Generally, the volume fraction of crystalline phase increases with increasing annealing temperature and time, accompanied by an increase of *ε*_*r*_ and decrease of BDS. The optimized *W*_rec_ is a balance between *ε*_r_ and BDS. Chen and co-workers^383^ reported that tungsten bronze structured, Ba_0.27_Sr_0.75_Nb_2_O_5.78_ phase formed from the Na_2_O–BaO–SrO–Nb_2_O_5_–SiO_2_–B_2_O_3_ glass matrix at 800 °C and a secondary phase NaSr_1.2_Ba_0.8_Nb_5_O_15_ emerged when crystallization temperature exceeded 850 °C. Remarkably high BDS ∼ 1400 kV cm^–1^ with *ε*_r_ of ∼50 were obtained, leading to a *W*_rec_ = 4 J cm^–3^. Besides, Wang and co-workers reported ultrahigh *W*_rec_ of 20.7 J cm^–3^ in BaO–PbO–Na_2_O–Nb_2_O_5_–SiO_2_–Al_2_O_3_ (BPNN-AS) glass ceramics at the optimized crystallization temperature of 900 °C, as shown in Figure 24.^384^ With increasing crystallization temperature from 850 to 1000 °C, the BDS decreased from 1890 to 1440 kV cm^–1^ and the crystallinity increased from 64.5 to 97.3% (Figure 24b). Similar results were also reported in other glass ceramic systems, including Na_2_O–PbO–Nb_2_O_5_–SiO_2_,^385^ SrO–BaO–Nb_2_O_5_–B_2_O_3_,^386^ K_2_O–SrO–Nb_2_O_5_–SiO_2_–Al_2_O_3_–B_2_O_3_,^387^ BaO–Na_2_O–Nb_2_O_5_–SiO_2_–Al_2_O_3_,^388^ BaO–PbO–Na_2_O–Nb_2_O_5_–SiO_2_,^389^ SrO–Na_2_O–Nb_2_O_5_–SiO_2_,^390^ and Bi_2_O_3_–Nb_2_O_5_–SiO_2_–Al_2_O_3_.^391^

**Figure 25:**
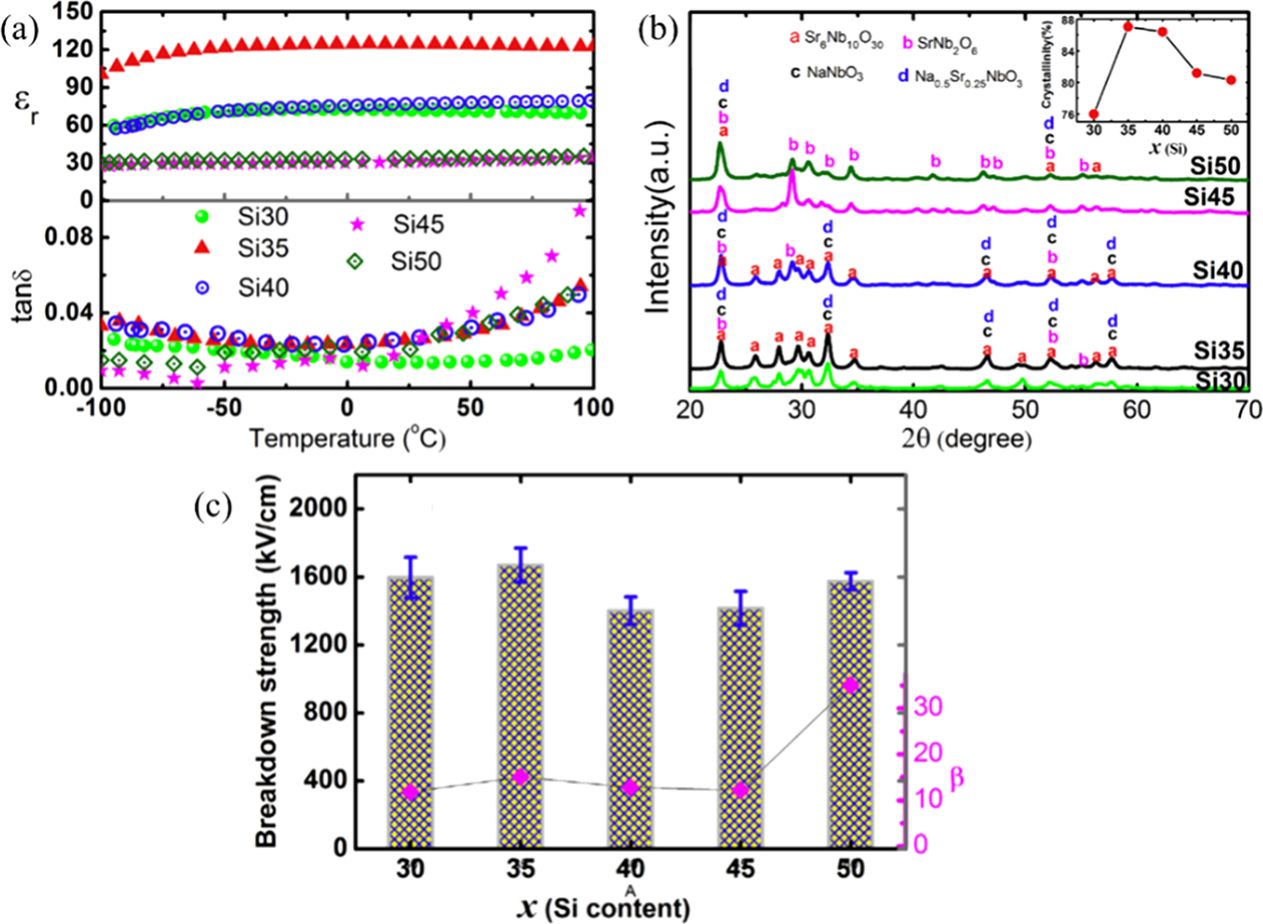
(a) Dielectric properties, (b) XRD patterns, and (c) BDS of SNN-Si glass ceramics as a function of SiO_2_ concentration (β). Reproduced with permission from ref (392). Copyright 2017 Elsevier.

Each constituent oxide in the glass matrix has an important effect on the crystal phase, microstructure, BDS, and energy storage properties. For example, SiO_2_ is an important and active studied constituent oxide in glass matrix. With increasing SiO_2_ content, *ε*_r_ of SrO–Na_2_O–Nb_2_O_5_–SiO_2_ (SNN-Si) glass ceramics first increased and then decreased as shown in Figure 25a, which was attributed to the change of volume fraction Sr_6_Nb_10_O_30_ (Figure 25b). The optimal *ε*_r_ of 120 and BDS ∼ 1700 kV cm^–1^ were obtained with 35 mol % SiO_2_ (Figures 25a,c), resulting in the highest theoretical *W*_rec_ of 15.2 J cm^–3^.^392^ Wang and co-workers reported that, as K_2_O concentration increased in K_2_O–BaO–Nb_2_O_5_–SiO_2_ glass ceramics, grain boundary *R* and activation energy decreased, indicating the decrease of interfacial polarization, leading to the enhancement of BDS to ∼1900 kV cm^–1^ and *W*_rec_ ∼ 12 J cm^–3^.^393^ They also reported that substitution of Sr for Ba in SrO–BaO–K_2_O–Nb_2_O_5_–SiO_2_ led to the formation of solid phase Sr_0.5_Ba_0.5_Nb_2_O_6_ and improvement of dielectric properties.^394^ The highest BDS of ∼1800 kV cm^–1^ and *W*_rec_ of 17.5 J cm^–3^ were achieved with Sr = 0.4 due to a uniform and dense microstructure and lower interfacial polarization. Li and co-workers reported substitution of K with Ba in SrO–BaO–K_2_O–Nb_2_O_5_–B_2_O_3_–P_2_O_5_ glass ceramics transformed Ba_0.5_Sr_0.5_Nb_2_O_6_ to a solid solution of K_2*xy*_Ba_(1-*x*)*y*_Sr_5–*y*_Nb_10_O_30_ and then KSr_2_Nb_5_O_15_ phase, leading to a decrease in *ε*_r_.^395^ A maximum theoretical *W*_rec_ of 12.8 J cm^–3^ was obtained under BDS ∼ 1800 kV cm^–1^, along with dielectric loss <0.3%. Liu and co-workers studied the effect of R_2_O (R = Li, Na, K) on the phase structure, dielectric properties and BDS in BaO–R_2_O–Nb_2_O_5_–SiO_2_ glass ceramics, where the highest *W*_rec_ ∼ 19 J cm^–3^ was achieved with *ε*_r_ ∼ 22 and superior BDS of ∼4400 kV cm^–1^ in composition of BaO–Na_2_O–K_2_O–Nb_2_O_5_–SiO_2_.^396^

Compared with alkali based compositions, alkali-free glass compositions were found to deliver lower dielectric loss and fewer defect microstructure. For example, Smith and co-workers reported both ultrahigh *E*_max_ ∼ 12000 kV cm^–1^ and *W*_rec_ ∼ 35 J cm^–3^ in BaO–B_2_O_3_–Al_2_O_3_–SiO_2_ glass.^397^ The effect of Al/Si ratio on the modification of the microstructure and properties of SrO–BaO–Nb_2_O_5_–SiO_2_–Al_2_O_3_ glass ceramics was also studied by Xiu and co-workers.^398^

Additionally, rare-earth oxides, such as La_2_O_3_,^399−401^ Sm_2_O_3_,^402^ Yb_2_O_3_,^403,404^ and Gd_2_O_3_,^405^ are also commonly substituted into glass formulations for energy storage applications. Rare-earth oxides are mainly reported to act as nucleating agents^399,402^ or crystal growth inhibitors.^400,406^ Zhang and co-workers revealed that La_2_O_3_ leads to a homogeneous microstructure in the BaO–SrO–TiO_2_–Al_2_O_3_–SiO_2_ glass-ceramics which improved BDS ∼ 1600 kV cm^–1^ and W_rec_ ∼ 3.2 J cm^–3^ (2.5 times of the glass-ceramics without La_2_O_3_).^399^ Zheng and co-workers reported that 0.5 mol % La_2_O_3_ in SrO–BaO–Nb_2_O_5_–B_2_O_3_–ZnO glass-ceramics also optimized *ε*_r_ (∼130) and *W*_rec_ ∼ 7.1 J cm^–3^ through achieving a BDS ∼ 1100 kV cm^–1^ due to a reduction in crystallite size and precipitation of high *ε*_r_ phase, Sr_0.5_Ba_0.5_Nb_2_O_6_.^400^ A similar effect was reported for Sm_2_O_3_ by Chen and co-workers in the SrO–BaO–Nb_2_O_5_–B_2_O_3_–ZnO glass ceramics with *W*_rec_ of 8.2 J cm^–3^ at 1100 kV cm^–1^.^402^ Moreover, Yb_2_O_3_ is reported to eliminate the impurity phases and form a uniform microstructure in BaO–SrO–TiO_2_–Al_2_O_3_–B_2_O_3_–SiO_2_ glass-ceramics, leading to *W*_rec_ of 3.5 J cm^–3^, ∼ 1.8 times higher than undoped compositions.^404^

**Figure 26:**
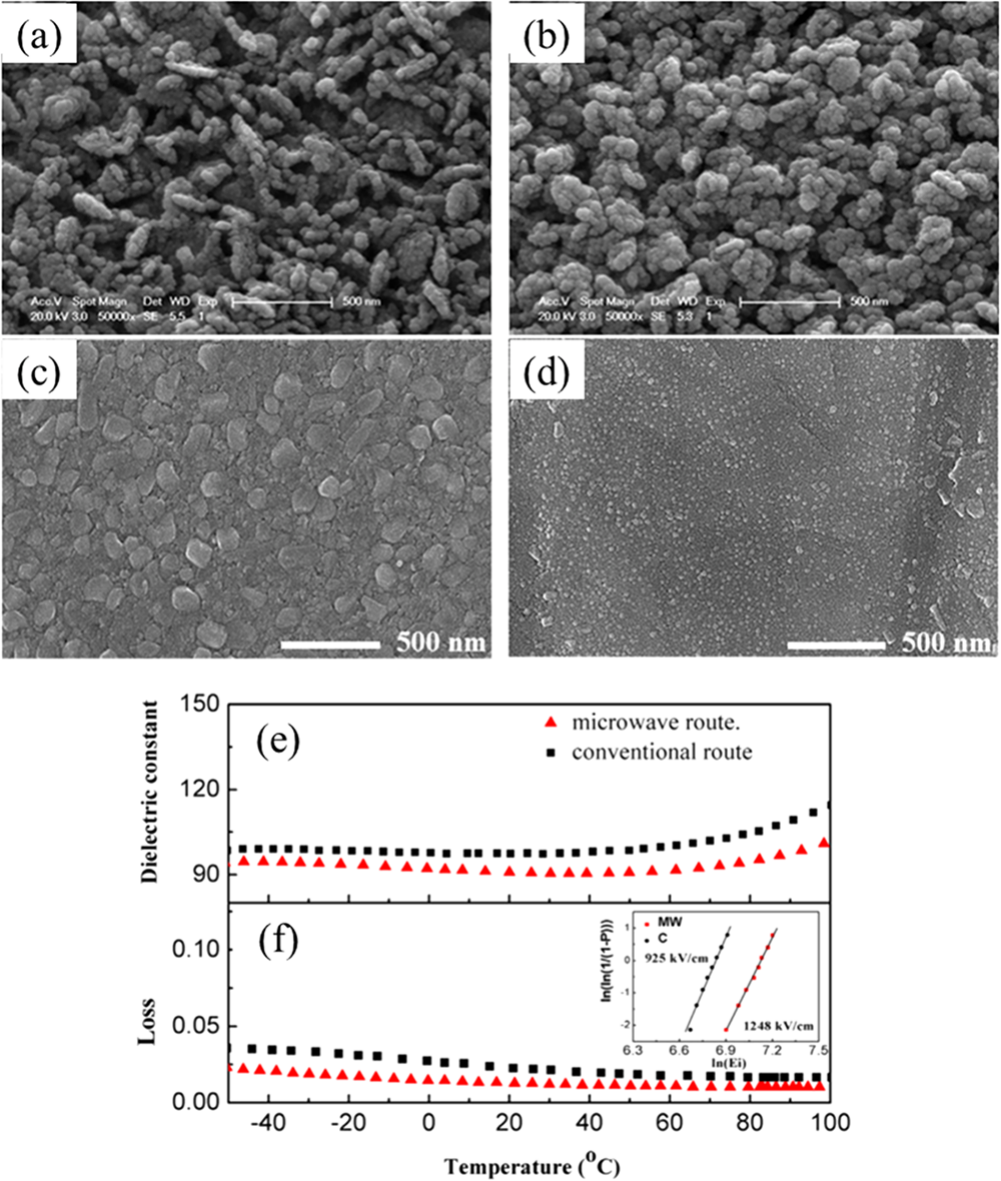
SEM images of BST-BBAS samples annealed at 950 °C by (a) conventional method and (b) microwave treatment.^408^ SEM images of BNNS samples annealed at 1000 °C by (c) conventional method and (d) microwave treatment. (e, f) Temperature dependence of dielectric properties of BNNS samples. The BDS plot is inset in (f).^409^ (a, b) Reproduced with permission from ref (408). Copyright 2014 Elsevier; (c−f) Reproduced with permission from ref (409). Copyright 2017 Elsevier.

Apart from the conventional annealing, novel methods such as microwave treatment have been reported to improve the energy storage properties of glass ceramics. Zhang and co-workers found that microwave treatment restrained the formation of the dendritic microstructure in Ba_*x*_Sr_1-*x*_TiO_3_−(Ba−B−Al−Si−O) (BST−BBAS) glass-ceramics (Figure 26), leading to the improvement of BDS from 1200 kV cm^–1^ to 1500 kV cm^–1^, corresponding to *W*_rec_ of 2.8 J cm^–3^ (950 °C anneal).^408^ Xiao and co-workers further reported that the precipitation of impurity phases in the K_2_O–SrO–Nb_2_O_5_–SiO_2_–Al_2_O_3_–B_2_O_3_ glass-ceramics was limited by controlling the crystallization time using microwave sintering, with optimum *ε*_r_, BDS of 1400 kV cm^–1^ and maximum theoretical *W*_rec_ (∼9 J cm^–3^) obtained after 10 min.^407^

#### 3.1.4 Summary of State-of-the-Art in Ceramics

The debate over whether lead-free electroceramics can replace their lead-based counterparts has been ongoing for over two decades. Lead based compositions generally outperform their lead-free counterparts on most metrics. Moreover, lead-free compositions are disparate with a large number of different formulations potentially required to cover the properties achieved with essentially doped PZT. Provided, however, that the performance, reliability and cost of lead-free are competitive with PZT, it is highly likely that lead-free electroceramics will begin to replace their lead-based equivalents and attain large scale production in the coming years as a consequence of environmental legislation.^31,103,415^

Of all the applications, lead-free high energy density capacitors are the most likely to see large-scale production since (i) the performance of lead-free compositions is approaching that of lead-based; (ii) reduction in intrinsic electrical properties may be compensated by increasing the BDS often through decreasing layer thickness (see section 3.1.2); and (iii) the capacitor industry is dominated by lead-free BT-based MLCCs, and thus, there is an expectation that the related products will not contain lead.^34,45,90,209,276,416^ This latter statement does not hold for piezoelectric ceramics market which is dominated by PZT and its derivatives.^174,417−419^

**Figure 27:**
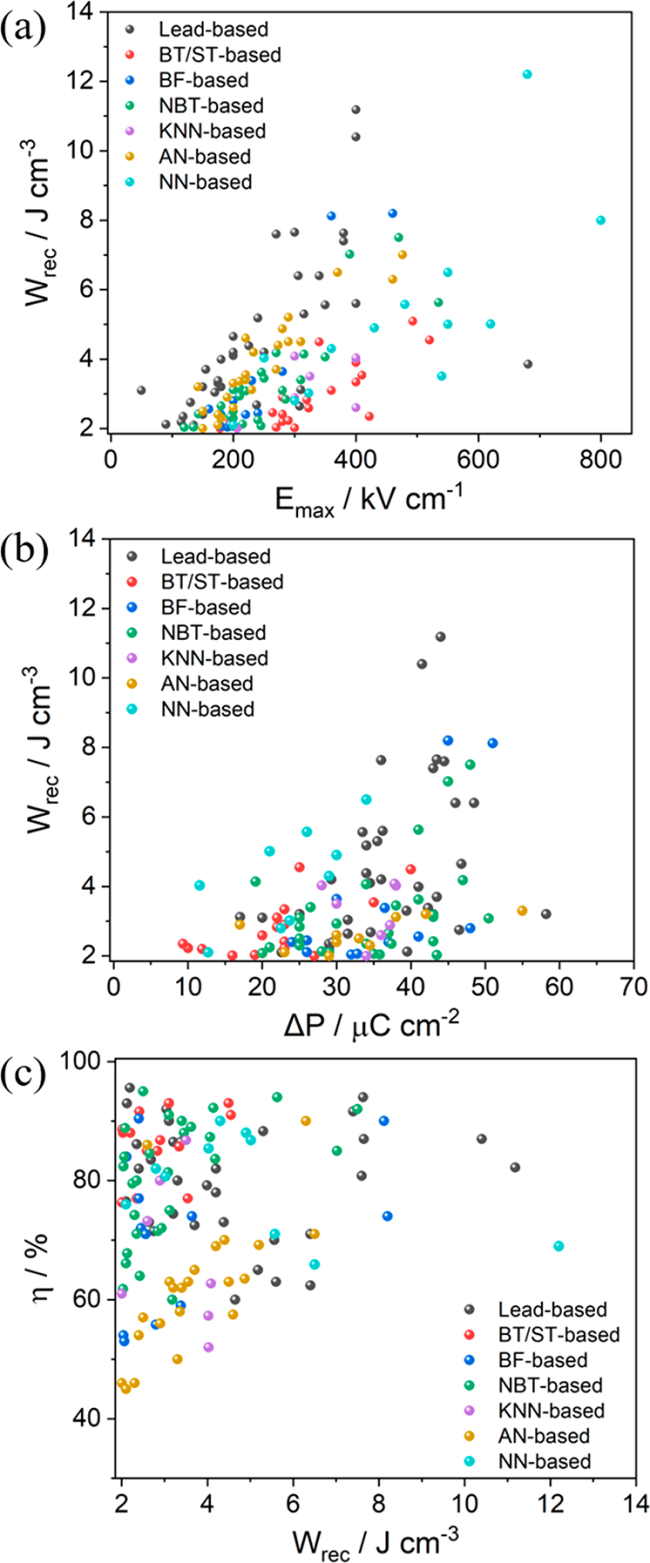
Comparison of (a) *E*_max_ vs *W*_rec_; (b) *ΔP* vs *W*_rec_; and (c) *W*_rec_ vs η for lead-based/lead-free bulk ceramics. *t of the bulk ceramics is commonly >0.1 mm.

The energy storage performances, *E*_max_, *ΔP*, *W*_rec_, and η, for lead-based and lead-free ceramics are summarized and plotted in Figure 27 (note: glass ceramics are not included). A comparison of *W*_rec_ vs *E*_max_ for different lead-based/lead-free bulk ceramics is displayed in Figure 27a. Lead-based bulk ceramics have the advantage of both high *E*_max_ (up to ∼400 kV cm^–1^) and *W*_rec_ (up to ∼12 J cm^–3^) with respect to lead-free candidates. NN-based ceramics currently offer the highest *W*_rec_ under high *E* (>350 kV cm^–1^) for lead-free compositions, followed by AN-, NBT-, BF-, and KNN-based materials. BT and ST-based ceramics display the lowest *W*_rec_ in spite of their high *E*_max_ of ∼450 kV cm^–1^, but they are perhaps the most appealing dielectrics commercially since they are the current basis of MLCC production.

*ΔP* vs *W*_rec_ is compared in Figure 27b. Lead-, NBT-, and BF-based materials exhibit extraordinarily high *ΔP* (up to ∼60 μC cm^–2^), followed by AFE NN- and AN-based (up to ∼40 μC cm^–2^) and KNN-based materials (up to ∼35 μC cm^–2^) with the lowest (up to ∼25 μC cm^–2^) for BT- and ST-based materials. Bicontaining electroceramics such as NBT and BF, have been heavily studied recently as potential lead-free electroceramic materials due to their large polarization.^260,266,281,282,284,420−423^ A high *ΔP* (60 μC cm^–1^) is obtained in NBT and BF based by reducing the *P*_*r*_ through chemical substitution with other perovskite end members to form a relaxor with an ultraslim *P–E* loop. To some extent therefore, the advantage of a high intrinsic polarization end member such as BF is weakened. Intermediate *ΔP* ∼ 40 μC cm^–2^ values are observed for AFEs such as AN- and NN-based materials but *P*_max_ is often limited as compositions exhibit polarization saturation as a function of applied field.

Figure 27c compares *W*_rec_ vs η for a wide range of compositions. ST-based materials display the best η (∼90%) due to their linear-like dielectric behavior. For BT-, NBT-, and lead-based materials, η varies with the material composition since it is a function of many factors. Dielectric loss associated with defects such as *V*_*O*_^..^ play a role but primarily at high field and high frequency, energy is dissipated during the transition to a field induced long-range ordered state which is manifested by the opening of the *P–E* loop. If the transition is smeared over for the operational range through alloying or doping to create a so-called “weakly coupled relaxor state”, η > 90% can be achieved.^43,209,337^

High leakage current and electrical conductivity are considered as major challenges in BF and KNN-based materials but are addressed by appropriate doping, e.g. donor doping to mitigate *p*-type conductivity in BF-based ceramics.^45^ In addition, AFE based materials generally suffer from opening of the polarization loop above switching field to form a field induced FE phase which is detrimental to η. Compositional modifications to AN and NN ceramics aim not only to push the AFE-FE transition to higher field and stabilize the AFE phase but also to disrupt the long-range ordering in the field induced FE phase, thereby creating a slimmer portion of the *P–E* loop (higher η) than that being observed in unmodified materials.^366^

Apart from the parameters discussed above (*E*_max_, *ΔP*, *W*_rec_, and η), temperature and frequency stability are also important for practical applications. In the future, high energy density ceramic capacitors will be placed closer to the core engine electronics to optimize the equivalent circuit resistance. Therefore, the temperature requirement for energy storage ceramics is anticipated to increase. According to the white paper “Multilayer Ceramic Capacitors for Electric Vehicles” published by Knowles capacitors in 2017,^424^ the explosive development of EVs has prompted the appearance of new 200 °C-stable C0G type I dielectric ceramic capacitor on the market. However, these materials still do not fulfill the required high power/voltage, energy density, and temperature requirements (∼250 °C) to facilitate use near-engine. Better frequency stability from 100 Hz to 100 kHz is required to reduce power fluctuations when capacitors are used for DC/DC conversion for battery charging and DC/AC conversion for propulsion.^425−429^ Enhanced frequency stability also enables the capacitor to be compatible with diodes and thyristors for power switching and control.^430^

**Figure 28:**
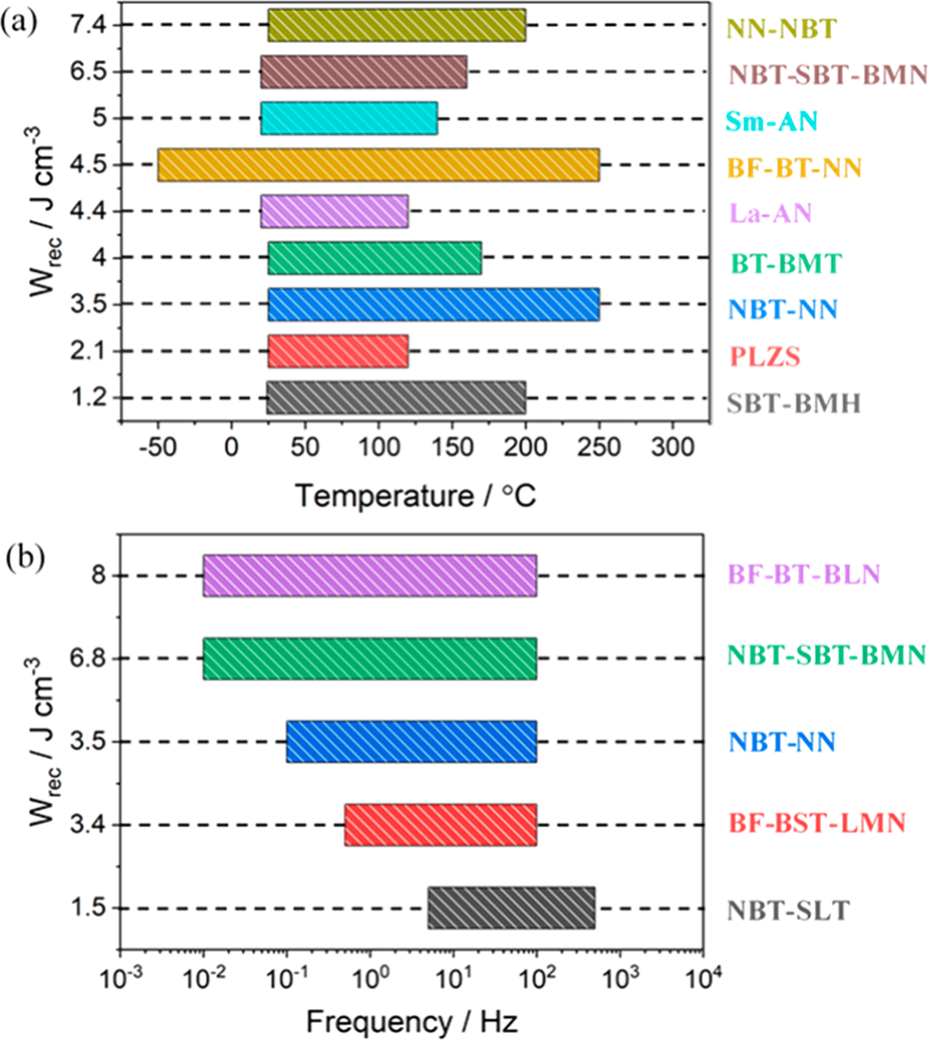
(a) Temperature-^42,43,130,131,208,242,297,337,348,352,357,359^ and (b) frequency-dependent *W*_rec_ for some reported electroceramic materials for high energy density capacitors.^276,295,297,337,416^

The temperature and frequency stabilities of many high energy density ceramics are evaluated, as shown in Figure 28. Only two compositions to date deliver *W*_rec_ > 3.5 J cm^–3^ up to 250 °C, 0.57BF–0.33BT–0.1NN^43^ and 0.78NBT–0.22NN.^297^ Most other compositions either do not sustain or do not have properties reported >200 °C. Typical issues associated with operating at higher temperature include, widening of the P–E loop or early breakdown due to high leakage current and electric field and/or temperature-induced phase transitions. High leakage currents above 200 °C typically arise from oxygen vacancy diffusion.^45^

Most compositions have been shown to deliver *W*_rec_ at a few hundred Hz but higher frequencies (>kHz) are rarely reported. Wang and co-workers, for example, discussed frequency stability from 10^–2^ to 10^2^ Hz for 0.57BF-0.3BT–0.13BLN with *W*_rec_ ∼ 8 J cm^–3^ and η ∼ 81% at 400 kV cm^–1^. All electroceramics for capacitors appear to deliver a charging–discharging speed at or faster than 1 μs. Short times of τ_0.9_ ∼ 0.15 μs (90% of energy discharge in 0.15 μs) were reported by Li and co-workers in BT based ceramics at 150 kV cm^–1^^197^ while Qi et al. described even faster charging–discharging speeds (τ_0.9_ ∼ 97 ns) in BF-based ceramics at 200 kV cm^–1^.^42^ Increasing attention has also been focused on fatigue-resistant behavior as a performance metric in practical applications with 10^4^ cycles (*W*_rec_ variation <10%) reported in BF-based ceramic multilayers from 20 to 100 °C, coupled with a low value of electrostrain (<0.03%).^276^

### 3.2 Ceramic Multilayers and Films

#### 3.2.1 Ceramic Multilayers

**Figure 29:**
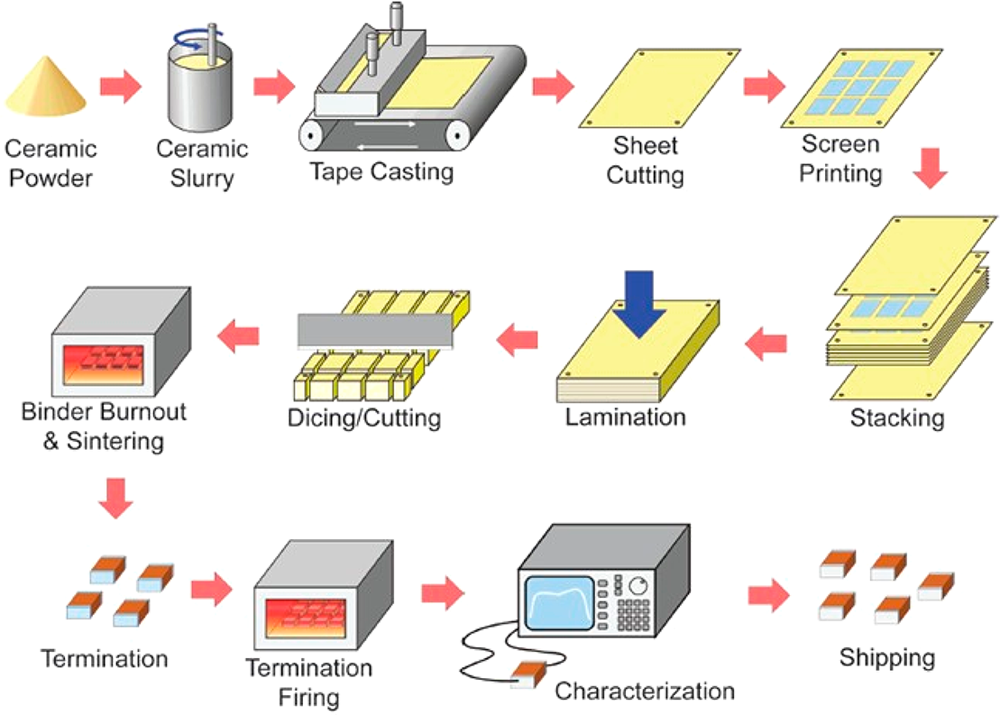
Ceramic MLs fabrication process (MLs cofire technology). Reproduced with permission from ref (18). Copyright 2010 IEEE.

Ceramic MLs are fabricated by a series of processing steps which include slurry preparation, tape-casting, screen printing, lamination, cosintering, and termination, as shown in Figure 29.^18,176,422,431,432^ This fabrication technology is a powder-based approach that accommodates scale-up from laboratory research to commercial manufacturing. The market of ceramic MLs ∼ $5.3 billion in 2017 but will reach ∼ $7.8 billion by 2024 for electronic applications, including but not limited to mobile phones, laptops and motor vehicles.^433^ Advanced high energy density ceramic MLs, based on AFEs and RFEs materials, are being developed to facilitate power electronics within hybrid electric vehicles which require higher *W*_rec_ and operating temperature. Simultaneously, research into low cost internal electrodes is required so that the highest performant ceramics can be developed. The energy storage properties for different ceramic MLs are summarized in Table 12.

**Table 12:** Summary of the Energy Storage Performance of Ceramic MLs

materials	*t* (μm)	electrode	*E* (kV cm^–1^)	*ΔP* (μC cm^–2^)	*W*_rec_ J cm^–3^	η (%)	ref
[0.94(0.75NBT–0.25NN)–0.06BT]–0.1CaZrO_3_	30	70Ag/30Pd	120		0.35	77	(440)
BT + BT@SiO_2_ *layer structure	10/20		301.4	18	1.8	71.5	(441)
ST + Li_2_CO_3_)/(0.94NBT–0.06BT) *layer structure	50/12		237	30	2.41	68	(442)
(Pb_0.88_Ba_0.05_La_0.02_Dy_0.04_) (Zr_0.68_Sn_0.27_Ti_0.05_)O_3_	11	95Pd/5Ag	300	28	2.7	67.4	(435)
0.6NBT–0.4ST	27	75Ag/25Pd	270		2.83	85	(443)
Pb(Zr_0.95_Ti_0.05_)_0.98_Nb_0.02_O_3_	32				3		(434)
Nb and Mn co-doped 0.9BT–0.1NBT	30	60Ag/40Pd	480	18	3.33	80	(444)
Ca(Zr_0.80_Ti_0.20_)O_3_	10	Pt	1500	6	4		(445)
BT–0.12 Bi(Li_0.5_Ta_0.5_)O_3_	30	Pt	466	25	4.05	95.5	(203)
BT–0.12 Bi(Li_0.5_Nb_0.5_)O_3_	29	Pt	450	25	4.5	91.5	(198)
0.7BT–0.3BiScO_3_	25	Pt	730	24	6.1		(33)
15% Nd doped BF–BT	33	Pt	540	39	6.74	77	(90)
BT−0.13Bi[Zn_2/3_(Nb_0.85_Ta_0.15_)_1/3_]O_3_	11	60Ag/40Pd	790	27	7.8	88	(446)
BT−0.13Bi[Zn_2/3_(Nb_0.85_Ta_0.15_)_1/3_]O_3_	11	60Ag/40Pd	750	27	8.13	95	(437)
NBT–0.45(Sr_0.7_Bi_0.2_)TiO_3_	30	Pt	720	30	9.5	92	(222)
BF–0.3Ba_0.8_Sr_0.2_TiO_3_–0.06La(Mg_2/3_Nb_1/3_)O_3_	8	Pt	720	43	10	77	(416)
BT−0.13Bi[Zn_2/3_(Nb_0.85_Ta_0.15_)_1/3_]O_3_	5	60Ag/40Pd	1047	30	10.12	89.4	(438)
BT−0.13Bi[Zn_2/3_(Nb_0.85_Ta_0.15_)_1/3_]O_3_	9	60Ag/40Pd	1000	30	10.5	93.7	(447)
BF–0.3BT–0.08Nd(Zr_0.5_Zn_0.5_)O_3_	17	Pt	700	34	10.5	87	(34)
Pb_0.98_La_0.02_(Zr_0.7_Sn_0.3_)_0.995_O_3_	20	Pt	560	50	12.6	80	(436)
BF–0.3BT–0.13 Bi(Li_0.5_Nb_0.5_)O_3_	8	Pt	953	45	13.8	81	(276)
BT−0.13Bi[Zn_2/3_(Nb_0.85_Ta_0.15_)_1/3_]O_3_	4.8	Pt	1500	36	14.1	69.7	(448)
0.50BF–0.40ST–0.10BMN	8	Pt	1000	50	15.8	80	(45)
NBT–0.30(Sr_0.7_Bi_0.2_)TiO_3_–0.08BMN	8	Pt	1000	50	18	93	(337)
BT−0.13Bi[Zn_2/3_(Nb_0.85_Ta_0.15_)_1/3_]O_3_@SiO_2_	4.7	60Ag/40Pd	1755	35	18.24	94.5	(89)
⟨111⟩-textured NBT–0.30SBT	20	Pt	1030	>65	21.5	80	(439)

**Figure 30:**
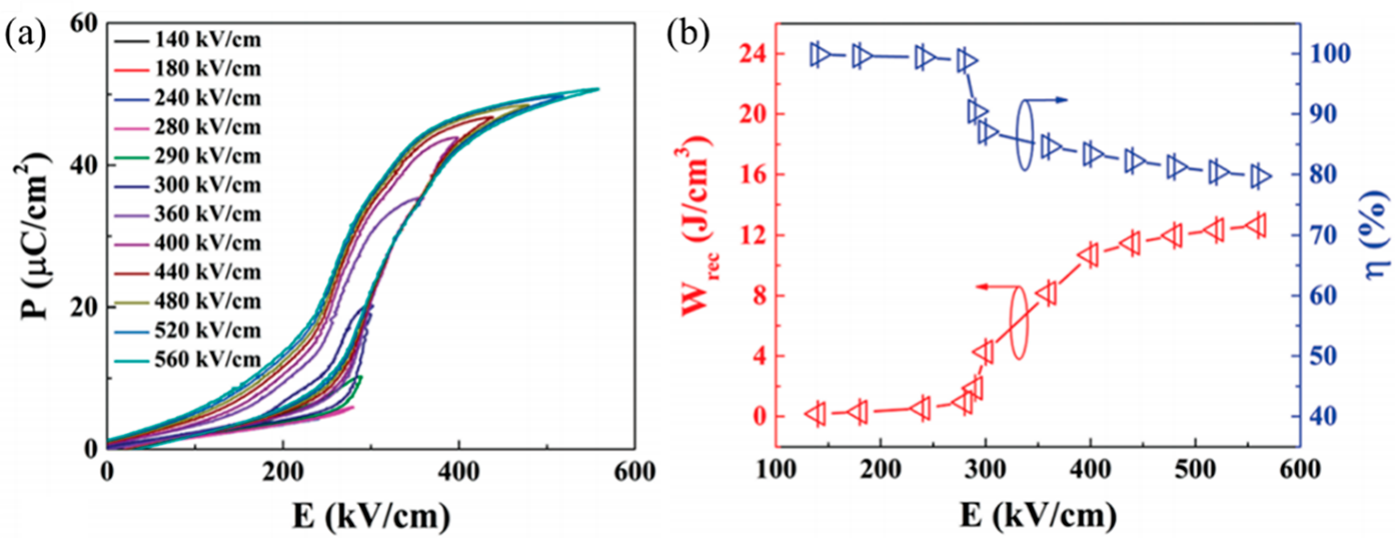
(a) *P–E* loops under electric field up to *E*_max_ (b) calculated *W*_rec_ and η values for Pb_0.98_La_0.02_(Zr_0.7_Sn_0.3_)_0.995_O_3_ ceramic ML. Reproduced with permission from ref (436). Copyright 2020 Royal Society of Chemistry.

Several lead-based AFE ceramic MLs have been reported using different internal electrodes. A giant power density ∼2000 kW cm^–3^ and *W*_rec_∼ 3 J cm^–3^ was obtained in Pb(Zr_0.95_Ti_0.05_)_0.98_Nb_0.02_O_3_ MLs using Pt as internal conductive electrode.^434^ Optimized performance of *W*_rec_ ∼ 3.8 J cm^–3^ was also reported by Hao and co-workers for (Pb_0.88_Ba_0.05_La_0.02_Dy_0.04)_(Zr_0.68_Sn_0.27_Ti_0.05_)O_3_ MLs using 5%Ag/95%Pd internal electrode. However, this was accompanied by a large electric field-induced strain ∼0.71% at 300 kV cm^–1^,^435^ a major drawback for practical applications due to inferior mechanical stability in operation. To date, Hao and co-workers have reported the record-high *W*_rec_ ∼ 12.6 J cm^–3^ and η ∼ 80% under *E*_max_ ∼ 560 kV cm^–1^ for Pb_0.98_La_0.02_(Zr_0.7_Sn_0.3_)_0.995_O_3_ ceramic ML, as shown in Figure 30.^436^

**Figure 31:**
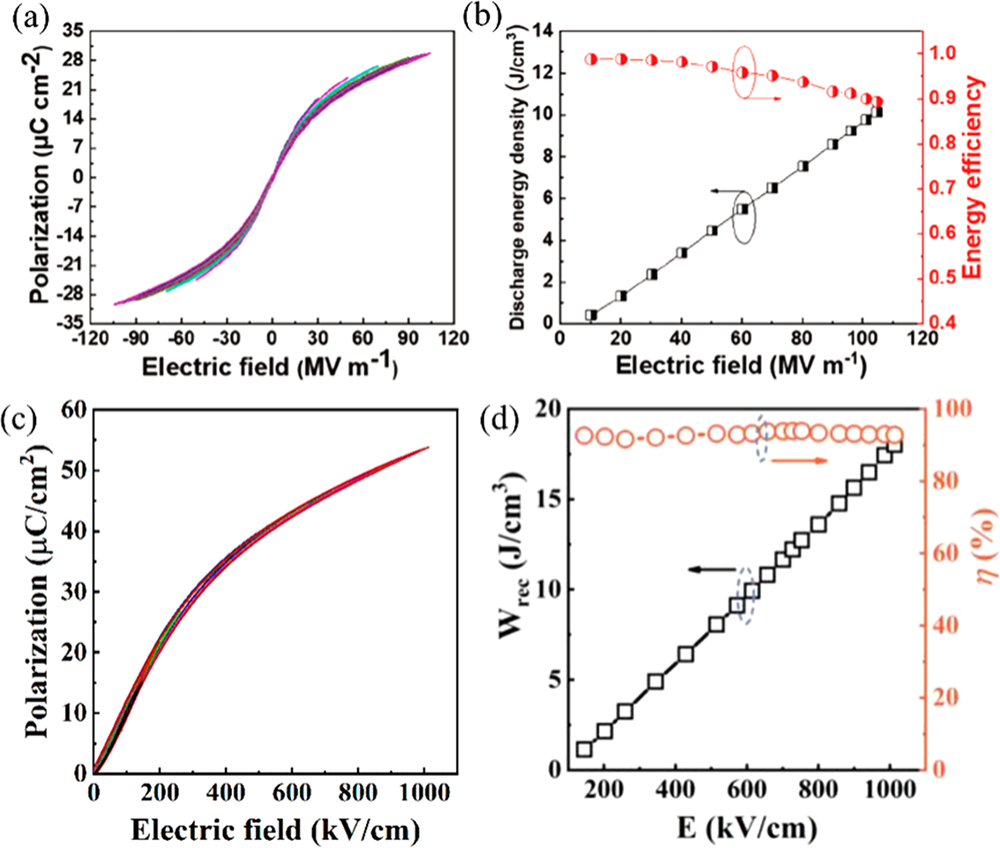
(a) Bipolar *P–E* loops and (b) calculated *W*_rec_ and η of 0.87BT−0.13Bi[Zn_2/3_(Nb_0.85_Ta_0.15_)_1/3_]O_3_ MLs under different electric fields.^438^ (c) Unipolar *P–E* loop, with inset SEM micrograph of ceramic MLs, and (d) calculated *W*_rec_ and η of NBT–0.45SBT–0.08BMN ceramic MLs under different *E*. (a, b) Reproduced with permission from ref (438). Copyright 2019 John Wiley and Sons; (c, d) Reproduced with permission from ref (337). Copyright 2021 Elsevier.

Although lead-free AFE ceramics show great promise, there are no reports of ceramic MLs in the literature. Instead, lead-free RFEs have dominated recent ceramic ML research due to their excellent *W*_rec_ and fatigue-resistant behavior accompanied by negligible electric field induced strain. 0.87BT–0.13 Bi(Zn_2/3_(Nb_0.85_Ta_0.15_)_1/3_)O_3_ ceramic MLs have been fabricated with a dielectric layer *t* of 11 μm using 60Ag/40Pd internal electrodes that exhibit excellent *W*_rec_ ∼ 8.13 J cm^–3^ and η ∼ 95% at 750 kV cm^–1^.^437^ The energy storage properties of 0.87BT–0.13 Bi(Zn_2/3_(Nb_0.85_Ta_0.15_)_1/3_)O_3_ were further enhanced by Zhao and co-authors by decreasing the dielectric layer to ∼5 μm, achieving *W*_rec_ ∼ 10.12 J cm^–3^ at 1012 kV cm^–1^, Figure 31a,b, as well as demonstrating good temperature stability from 75 to 175 °C.^438^ NBT–0.45SBT ceramic MLs have also been reported to exhibit *W*_rec_ ∼ 9.5 J cm^–3^ with η ∼ 95% at 720 kV cm^–1^,^222^ which were further improved by forming a solid solution with a third perovskite end-member, 10%BMN to give *W*_rec_ ∼ 18 J cm^–3^ with η > 90% at 1000 kV cm^–1^, by Ji and co-workers, Figures 31c,d.^337^ The highest among all lead/lead-free ceramic MLs, *W*_rec_ ∼ 21.5 J cm^–3^ at ∼1030 kV cm^–1^, however, was achieved for textured NBT–0.3SBT ceramic MLs.^439^ Texturing was achieved through the use of <111>-oriented ST platelets which reduced field-induced strain at high field thus enhancing the BDS greatly. Combination of texturing with alloying with a third end member (BMN) in NBT–SBT may well represent an exciting path to achieve yet higher energy densities.

**Figure 32:**
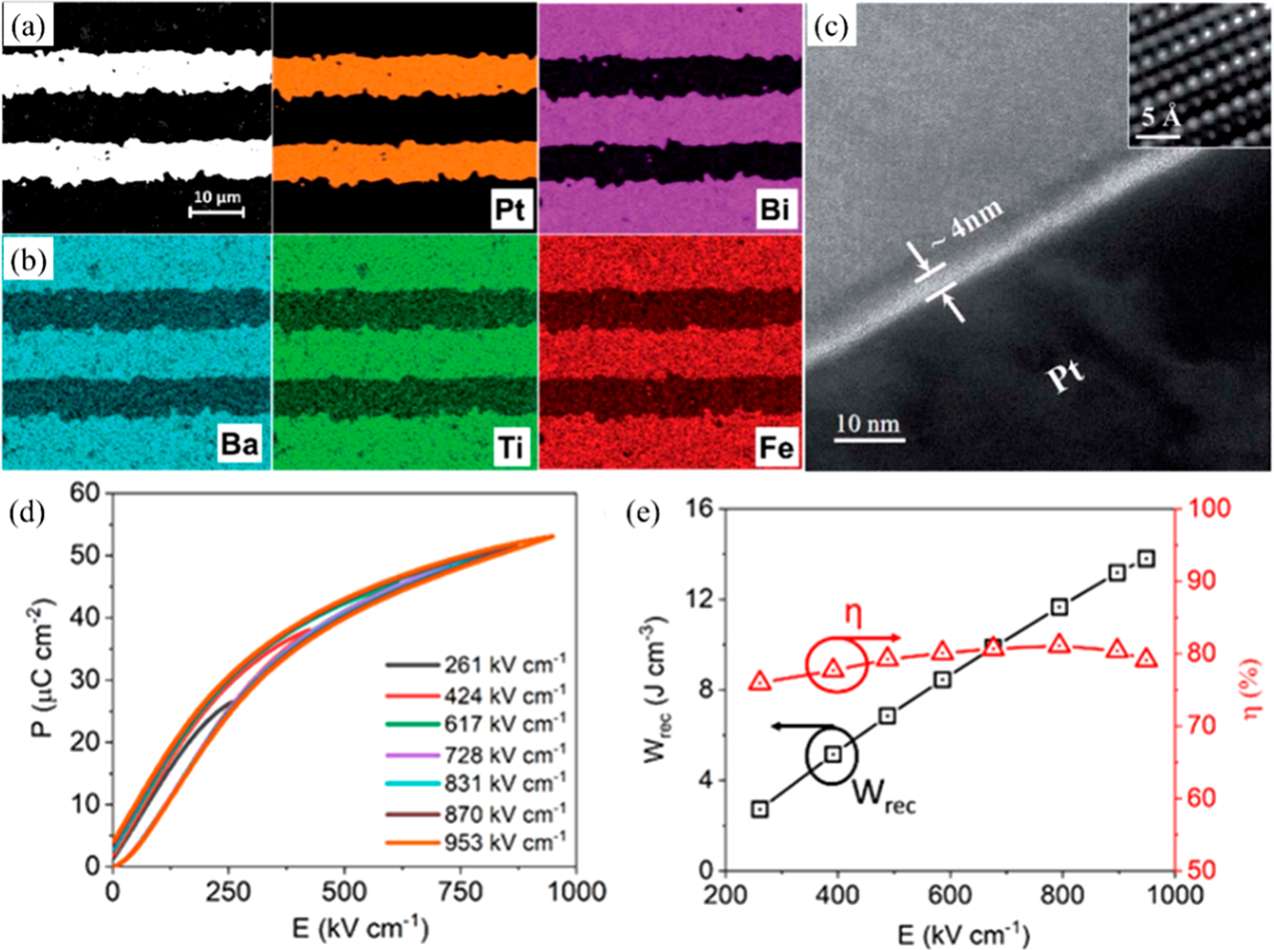
(a) Backscattering electron (BSE) cross section micrographs of BF–BT–0.13BLN ceramic MLs; (b) energy dispersive X-ray (EDX) mapping of all elemental distribution (c) transmission electron microscopy (TEM) micrograph obtained from an interface between a BF–BT–0.13BLN grain and a Pt grain (electrode); inset shows a high resolution TEM (HRTEM) image (filtered) obtained from the grain at a higher magnification. (d) Unipolar *P–E* loops and (e) calculated energy storage properties for BF–BT–0.13BLN ceramic MLs. Reproduced from ref (276). Copyright 2020 Royal Society of Chemistry.

Wang, Reaney, and co-workers have employed a range of different chemical dopants and alloying additions to investigate BF-(B,S)T-based ceramic multilayers.^34,45,90,276,416^ In 2018, Nd-doped BF-0.3BT was reported to exhibit *W*_rec_ ∼ 6.74 J cm^–3^ (more than 3 times higher than the bulk value) and η ∼ 77% at 540 kV cm^–1^ with a layer *t* of 33 μm.^90^ On the other hand, alloying BF–0.3BT with 8 mol % Nd(Zr_0.5_Zn_0.5_)O_3_ resulted in *W*_rec_ ∼ 10.5 J cm^–3^ with η of 87% at 700 kV cm^–1^ with a dielectric layer *t* of ∼17 μm, Figure 17.^34^ Further studies focused on promoting electrical homogeneity, which was considered to prevent conductive pathways developing in these composition, thereby avoiding the breakdown at high field and facilitating the improvement of *W*_rec_ by reducing the dielectric layer thickness. Finally, *W*_rec_ ∼ 13.8 J cm^–1^ at 953 kV cm^–1^ was achieved for BF–0.3BT–0.13 Bi(Li_0.5_Nb_0.5_)O_3_ (BF–BT–0.13BLN) ceramic MLs at a dielectric layer *t* of 8 μm, as shown in Figure 32.^276^

#### 3.2.2 Ceramic Films

Higher BDS and *W*_rec_ compared to MLs fabricated through powder-based technology have been reported for ceramic films deposited on LaNiO_3_/Si_(100)_ or Pt/Ti/SiO_2_/Si substrates by physical vapor or chemical deposition techniques, such as radio frequency magnetron sputtering,^449^ spin coating,^101,450,451^ pulsed laser deposition,^452^ and chemical solution deposition.^453^ The BDS of ceramic films is significantly improved due to the reduction of *t* (<1 μm) often attributed to fewer defects (grain boundaries) and/or pore/void concentration. Not only have higher figures of merit been reported for ceramic films, but several researchers have proposed novel underlying mechanisms (beyond reduction in defect density) behind the enhancement, such as the formation of polymorphic nanodomains.^452^ However, nonpowder based techniques are difficult to scale up into MLCCs, which may constrain these extraordinary results to lab-based fundamental research rather than promising practical output for commercial exploitation. The energy storage properties for ceramic films are summarized in Table 13.

**Table 13:** Energy Storage Properties for Different Ceramic Films

materials	*t* (μm)	*E* (kV cm^–1^)	*ΔP* (μC cm^–2^)	*W*_rec_ (J cm^–3^)	η (%)	ref
Pb_0.97_La_0.02_(Zr_0.97_Ti_0.03_)O_3_	1.7	1158	∼90	20.1	64	(460)
1 mol %Fe–doped 0.72NBT–0.18KBT–0.10ST	0.9	1200	58	20.34	35.17	(461)
Pb_0.97_Y_0.02_[(Zr_0.6_Sn_0.4_)_0.925_Ti_0.075_]O_3_	0.5	1300	70	21	91.9	(462)
Pb_0.92_La_0.08_(Zr_0.52_Ti_0.48_)O_3_	0.4	1600	35	22	77	(463)
Pb_0.85_La_0.1_ZrO_3_	0.45	1500	45	23.1	73	(464)
0.942(Na_0.535_K_0.480_NbO_3_)−0.058LiNbO_3_	5	1400	∼52	23.4	70	(465)
(Sr_0.85_Bi_0.1_)Ti_0.99_Mn_0.01_O_3_	0.25	1982	35	24.4		(466)
Pb_0.97_La_0.02_(Zr_0.98_Ti_0.02_)O_3_	2	984	∼120	25.2	52	(467)
Bi(Mg_0.5_Ti_0.5_)O_3_	0.11	900	70	26	55	(468)
(Pb_0.98_La_0.08_)(Zr_0.52_Ti_0.48_)O_3_	0.25	2200	40	27.5	62.2	(469)
6 mol % BF-doped (K_0.5_Na_0.5_)(Mn_0.005_Nb_0.995_)O_3_	1	2000	∼40	28	90	(470)
PbZrO_3_/PbZr_0.52_Ti_0.48_O_3_	0.35	2615	60	28.2	50	(471)
(Pb_0.92_La_0.08_)(Zr_0.65_Ti_0.35_)O_3_	0.32	3000	40	29.7	50.8	(472)
1 mol % Mn-doped NBT	1.2	2310	∼40	30.2	48	(455)
(K_0.5_, Na_0.5_)(Mn_0.005_, Nb_0.995_)O_3_-6 mol % BF	1	1900	45	31	90.3	(470)
0.9Pb(Mg_1/3_Nb_2/3_)O_3_–0.1PbTiO_3_	0.375	2640	35	31.3	40	(473)
0.9(0.94NBT–0.06BT)–0.1NN	0.3	3170	20	32	90	(474)
0.4 Bi(Mg_0.5_Zr_0.5_)O_3_–0.6PbTiO_3_	0.5	2000	43	32.3	51.4	(475)
2 mol % Fe-doped (Na_0.85_K_0.15_)_0.5_Bi_0.5_TiO_3_	1.15	2300	∼35	33.3	51	(456)
0.95NBT–0.05ST	1.5	1950	∼55	36.1	41	(476)
Ba_2_Bi_4_Ti_5_O_18_	0.41	2150	38	37.1	91.5	(477)
Pb_0.82_La_0.12_Zr_0.85_Ti_0.15_O_3_	1	2100	∼52	38	71	(478)
5.8 mol % SiO_2_ doped HfO_2_	0.01	∼5800		40	72	(479)
Pb_0.91_La_0.09_ (Zr_0.65_Ti_0.35_)_0.9775_O_3_	1	1998	∼65	40.2	62	(449)
0.89NBT–0.06BT–0.05BF	0.28	1750	150	42.9	65.7	(480)
BaBi_4_Ti_4_O_15_	0.45	2000	40	43.3	87.1	(481)
Bi_3.25_La_0.75_Ti_3_O_12_	0.5	2040	50	44.7	78.4	(482)
Hf_0.3_Zr_0.7_O_2_	0.0092	4500	30	46	53	(458)
Pb_0.97_La_0.02_Zr_0.66_Sn_0.23_Ti_0.11_O_3_	0.65	4000	55	46.3	84	(453)
0.5 Bi(N*i*_1/2_T*i*_1/2_)O_3_-0.5PT	0.455	2250	62	46.7		(483)
Pb_0.94_La_0.04_(Zr_0.98_Ti_0.02_)O_3_	2	3699	∼48	47.4	25	(454)
0.4ST-0.6 Bi_3.25_La_0.75_Ti_3_O_12_	0.3	2470	38	47.7	87.4	(484)
0.9 Bi_0.2_Sr_0.7_TiO_3_-0.1BF	0.46	4800	25	48.5	47.57	(485)
Si doped Hf_0.5_Zr_0.5_O_2_	0.01	∼3500	∼30	50	80	(486)
0.6NBT–0.4 Bi(Ni_0.5_Zr_0.5_)O_3_	1	2200	∼63	50.1	64	(457)
0.5 Bi(N*i*_1/2_T*i*_1/2_)O_3_-0.5PT-excess 20% PbO	0.45	2250	75	50.2		(487)
(Pb_0.97_La_0.02_)(Zr_0.7_Sn_0.25_Ti_0.05_)O_3_	1.8	3750	∼75	56	45	(488)
0.6BT–0.4 Bi_3.25_La_0.75_Ti_3_O_12_	0.3	3200	45	61.1	84.2	(489)
6 mol % Si-doped HfO_2_	0.01	4500		61.2	65	(490)
8 atom % Al doped HfO_2_	0.05	4900	14	63	90	(459)
Na_0.485_Bi_0.5_(Ti_0.96_W_0.01_Ni_0.03_)O_3_	0.6	2500	30	63.1	55	(491)
BF/Bi_3.25_La_0.75_Ti_3_O_12_	0.14	2753	50	65.5	74.2	(492)
Bi_0.525_Na_0.5_(Ti_.96_W_0.01_Ni_0.03_)O_3_	0.4	2500	78	65.8	52.9	(493)
0.25BF–0.75ST	0.5	4460		70	70	(494)
0.97(0.93NBT–0.07BT)–0.03BF	0.35	2285	∼120	81.9	64.4	(495)
(Ba_0.95_Sr_0.05_)(Zr_0.2_Ti_0.8_)O_3_	0.1	6200		102	87	(496)
0.25BF–0.3BT–0.45ST	0.45	4900	90	112	80	(452)
0.68PMN–0.32PT	0.15	5800	∼100	130	75	(497)

**Figure 33:**
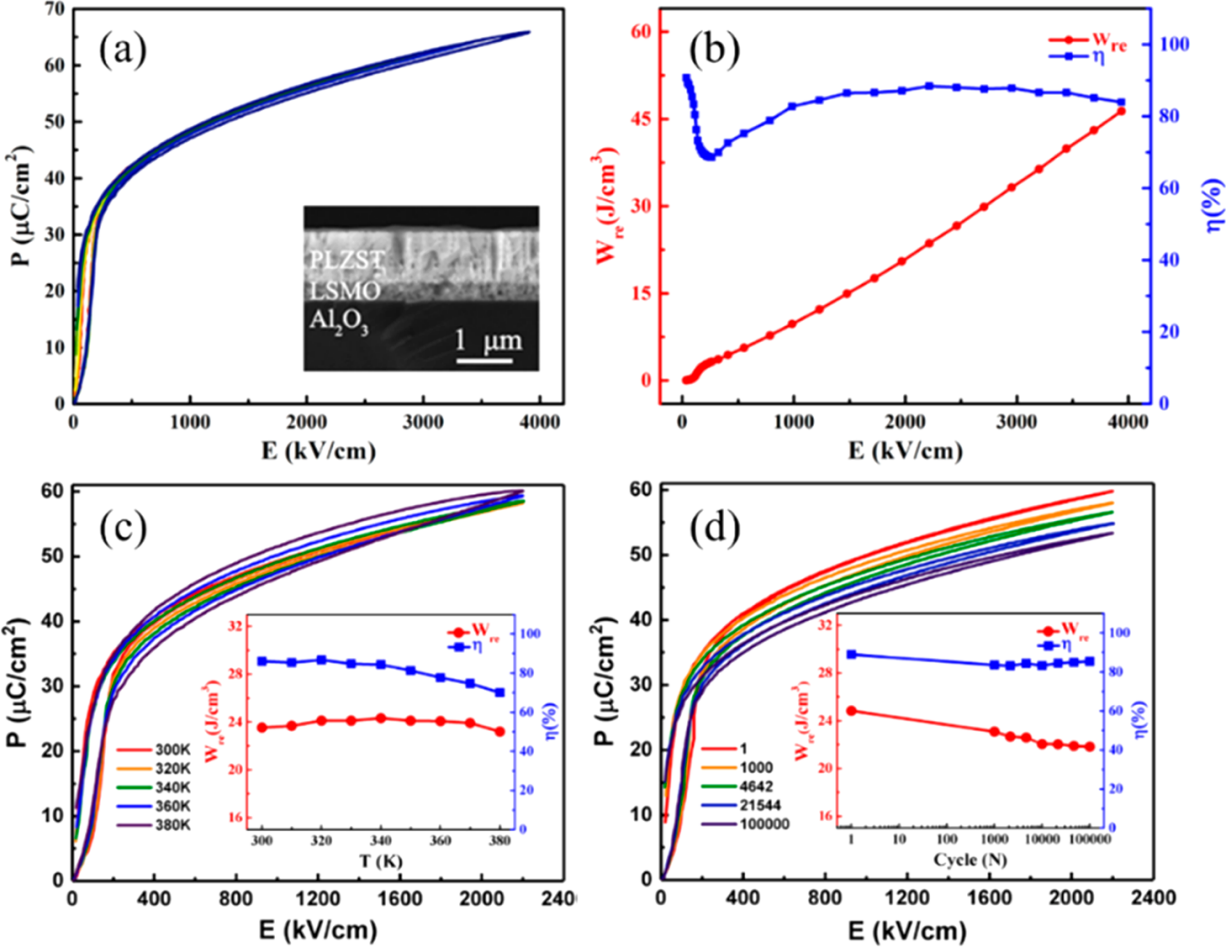
(a) Unipolar *P–E* loops, inset image is cross-section SEM image of ceramic film deposited on the substrate, and (b) *W*_rec_ and η of Pb_0.97_La_0.02_Zr_0.66_Sn_0.23_Ti_0.11_O_3_ ceramic film under various *E*. (c) Temperature and (d) cyclic unipolar *P–E* loops, *W*_rec_, and η of Pb_0.97_La_0.02_Zr_0.66_Sn_0.23_Ti_0.11_O_3_ ceramic film under E∼ 2200 kV cm^–1^. Reproduced with permission from ref (453). Copyright 2018 Elsevier.

Lead-based ceramic films have been studied heavily in the past decade using different preparation methods, particular for PLZT. (Pb_0.94_La_0.04_)(Zr_0.98_Ti_0.02_)O_3_ ceramic film with *t* of 2 μm was reported to exhibit 47.4 J cm^–3^ under electric field of 3700 kV cm^–1^ on a Pt_(111)_/TiO_2_/SiO_3_/Si_(100)_ substrate.^454^ Further increasing La concentration resulted in superior *W*_rec_ ∼ 40.2 J cm^–3^ and η ∼ 62%, achieved at *E*_max_∼ 1998 kV cm^–1^ for Pb_0.91_La_0.09_(Zr_0.65_Ti_0.35_)_0.9775_O_3_ on a LaNiO_3_/F-Mica substrates.^449^ In addition, for Pb_0.97_La_0.02_Zr_0.66_Sn_0.23_Ti_0.11_O_3_, an improved *W*_rec_ ∼ 46.3 J cm^–3^ was achieved at ∼4000 kV cm^–1^, accompanied by excellent temperature stability (up to 380 K) and cyclic reliability (up to 10^5^), Figure 33.^453^

**Figure 34:**
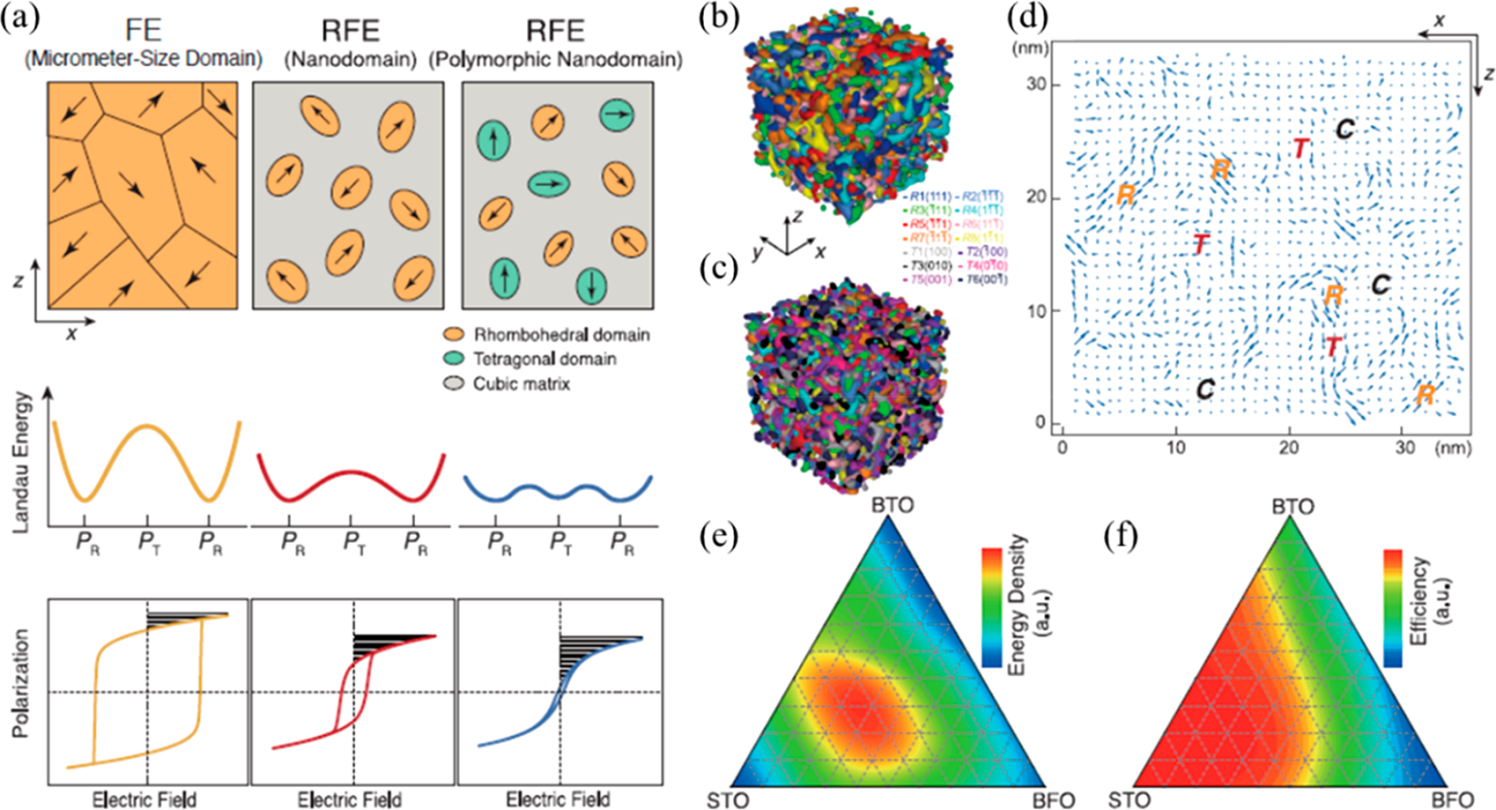
(a) Comparative display of Landau energy profiles and *P–E* loops of an FE with micrometer-size domains, an RFE with nanodomains, and an RFE with polymorphic nanodomains. Phase-field-simulated three-dimensional domain structures of (b) 0.45BF-0.55ST with rhombohedral nanodomains and (c) 0.20BF-0.25BT–0.55ST with coexisting rhombohedral and tetragonal nanodomains. (d) Simulation of the two-dimensional multiple nanodomain structure of 0.20BF-0.25BT–0.55ST in the cubic matrix. Contour plots of the simulated (e) *W*_rec_ and (f) η of BF–BT–ST solid solutions. Reproduced with permission from ref (452). Copyright 2019 The American Association for the Advancement of Science.

In the past few years, there has been increased focus on lead-free ceramic films due to concerns over toxicity of PbO. NBT and BF based relaxor compositions have dominated research and have competitive or even superior energy storage performance to lead-based films. Mn doped NBT ceramic films on a LaNiO_3_/Si_(100)_ substrates with *t* = 1.2 μm were reported to exhibit excellent *W*_rec_ ∼ 30.2 J cm^–3^ under *E*_max_ ∼ 2310 kV cm^–1^.^455^ Similar properties, *W*_rec_ ∼ 33.3 J cm^–3^ under *E*_max_ ∼ 2300 kV cm^–1^, were also obtained for Fe doped NBT–K_0.5_Bi_0.5_TiO_3_ ceramic film.^456^ Recently, even higher *W*_rec_ ∼ 50.1 J cm^–3^, η ∼ 63.9% accompanied by fast charge–discharge speed (∼210 ns) were achieved simultaneously at ∼2200 kV cm^–1^ in relaxor 0.6NBT–0.4 Bi(Ni_0.5_Zr_0.5_)O_3_ films.^457^ In addition, an ultrahigh *W*_rec_ ∼ 112 J cm^–3^ with η ∼ 80% was reported by Pan and co-workers in BF–BT–ST ceramic films recently (Figure 34). Polymorphic nanodomains with competitive rhombohedral and tetragonal phases with competitive free energy were considered critical for the extraordinary electrical properties. BF was chosen as a main component due to its large spontaneous polarization. BT was introduced to form a solid solution to encourage coexistence of rhombohedral and tetragonal phases and finally ST was incorporated to further disrupt the long-range polar coupling and induce polymorphic nanodomains. By tuning the ratio of BF, BT and ST, a highly disordered composition is produced with rhombohedral and tetragonal nanodomain. The experimental observations were validated by phase-field simulations in the optimized composition, 0.20BF–0.25BT–0.55ST.^452^ Apart from these perovskite lead-free relaxor candidates, HfO_2_-based ceramic films have also been explored and demonstrate promising energy storage properties, stabilities/reliabilities, scalability, and integration. *W*_rec_ ∼ 46 J cm^–3^ with excellent temperature stability (up to 175 °C) and cyclic fatigue resistant (up to 10^9^ time) was reported by Park in a 9.2 nm thick Hf_0.3_Zr_0.7_O_2_ film^458^ and *W*_rec_ ∼ 63 J cm^–3^ with η ∼ 85% were realized in 50 nm thick Al doped HfO_2_ ceramic films with excellent temperature and frequency stability.^459^

#### 3.2.3 Summary of State-of-the-Art in Ceramic MLs and Films

**Figure 35:**
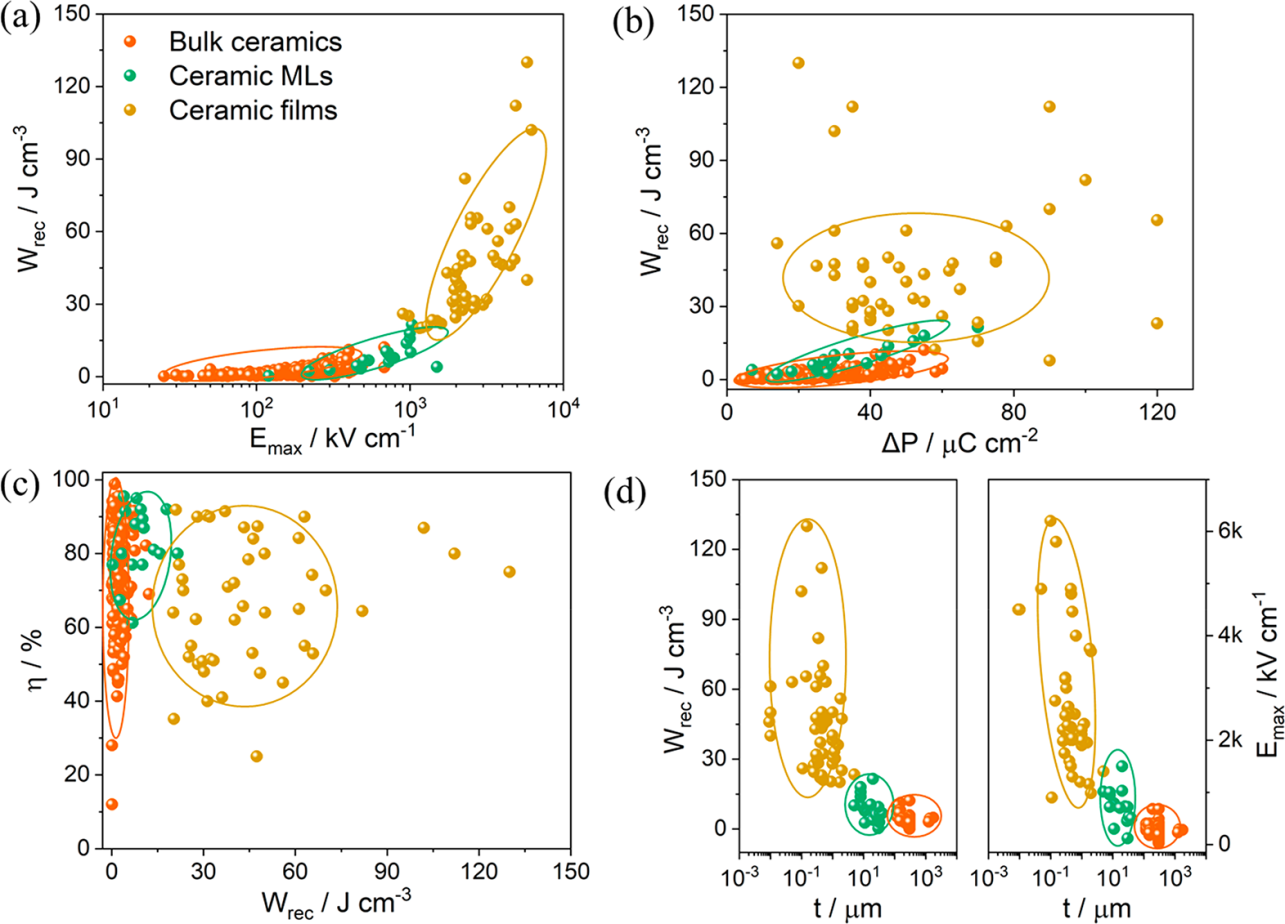
(a) *E*_max_ vs *W*_rec_, (b) *ΔP* vs *W*_rec_, (c) *W*_rec_ vs η, and (d) *t* vs *W*_rec_ and *E*_max_ between bulk ceramics, ceramic MLs and ceramic films.

The energy storage performances, *W*_rec_, η, and *ΔP*, between bulk ceramics, ceramic MLs, and ceramic films are shown in Figure 35. The highest *W*_rec_ (∼130 J cm^–3^) and *E*_max_ (∼5800 kV cm^–1^) are obtained in ceramic films, followed by ceramic MLs (∼21 J cm^–3^ and *E*_max_ ∼ 1000 kV cm^–1^) and bulk ceramics (*W*_rec_ (∼12 J cm^–3^ and *E*_max_ ∼ 650 kV cm^–1^) and scale primarily with *t* of the dielectric layer (Figure 35d). *W*_rec_ in ceramic films is also improved by higher *ΔP* (up to ∼120 μC cm^–2^) with respect to ceramic MLs (up to ∼70 μC cm^–2^) and bulk ceramics (up to ∼60 μC cm^–2^), as shown in Figure 35b. η for bulk ceramics, ceramic MLs and ceramic films varies significantly with composition and relates to factors such as, energy dissipation through a field induced transition to a long-range polar state, domain switching, polarization rotation, and leakage current relating to the presence of *V*_*O*_^..^ and associated defect dipoles.

From a commercial perspective, energy storage performance of lead-free ceramic MLs has improved significantly in the past few years with BF and NBT based ceramic MLs now rivalling lead-based ceramic MLs, delivering *W*_rec_ > 15 J cm^–3^ at ∼1000 kV cm^–1^. However, the selection of inner electrodes for these materials is limited to Pt, currently too costly for mass production. Commercial focus therefore, currently remains mainly on modified BT compositions which are compatible with Ni, Ag and Ag/Pd electrodes depending on composition and *p*O_2_ during fabrication. A huge stride forward in the industry would be the development of a low cost equivalent to Ag/Pd electrodes which could permit the fabrication of a wider range of MLs at higher *p*O_2_ which would inhibit the formation of *V*_*O*_^..^ and maintain high BDS.

## 4 Strategies for Improving Energy Storage Properties

The review of the state-of-the-art of ceramics, MLs, and films presented in section 3 clearly points to a set of criteria that are required to optimize energy storage performance. Though some of these have been alluded to in section 3 as part of the review of the state-of-the-art, it is worth collating these principals for RFE and AFE materials to act as guide for future materials development.

### 4.1 Optimization through an Induced Relaxor State

The most commonly utilized strategy to optimize energy storage properties is through inducing a relaxor state within a system that contains highly polarizable ionic species. It is typically carried out through strategic doping or alloying to form a pseudoternary solid solution. Levin and co-workers proposed that long-range ferroelectric correlations can be effectively “blocked” by using designed dopants with enhanced local polarizability.^498^ If this concept is married to dopant strategies to induce or maintain a homogeneous electrical microstructure, *ΔP* and BDS can be optimized leading to a large *W*_rec_.

**Figure 36:**
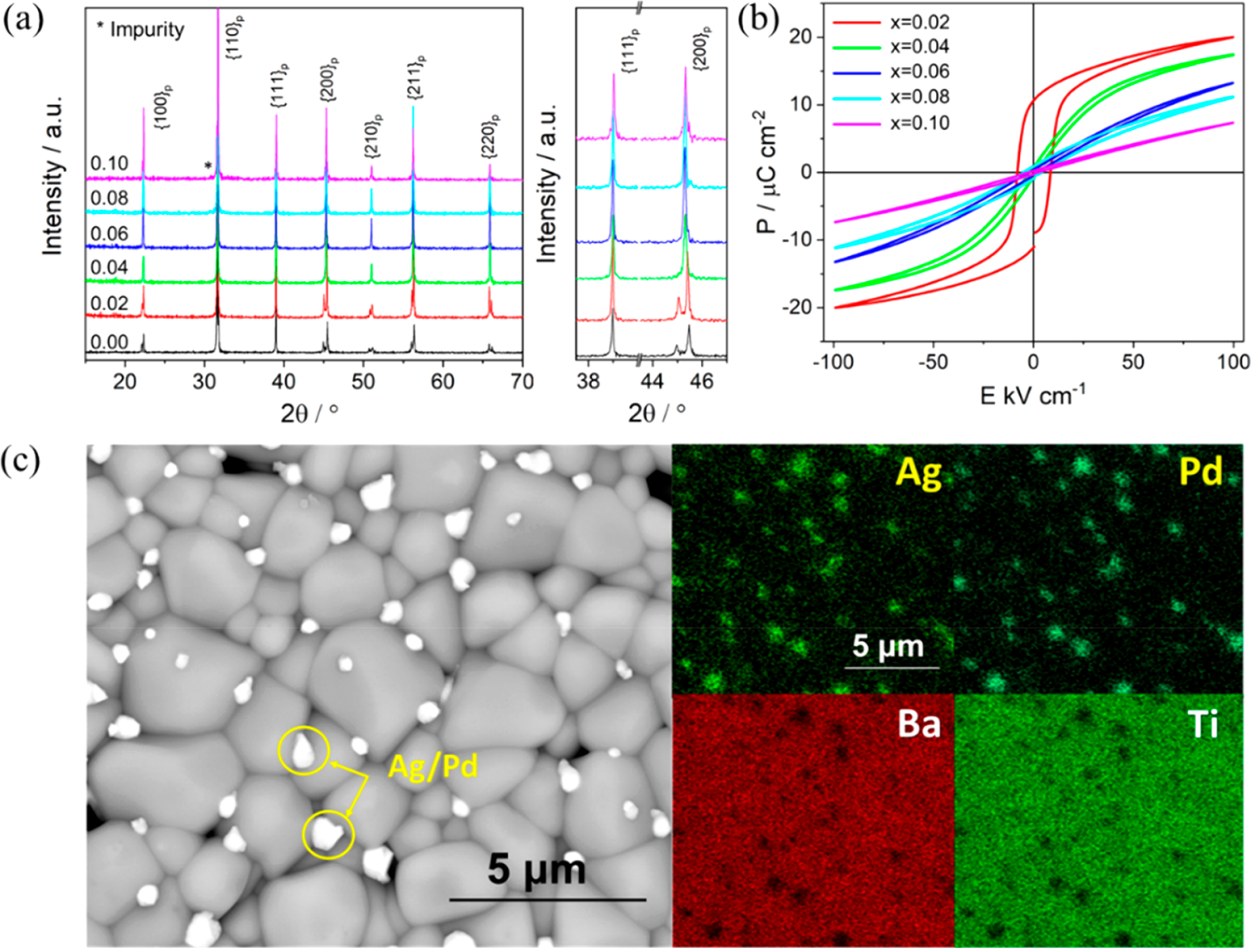
(a) XRD patterns with representative peaks and (b) Bipolar *P–E* loops for *x*B_2/3_MN–BT ceramics with *x* = 0.00–0.10. (c) BSE surface micrographs of Ag–Pd cofired 0.06B_2/3_MN–BT ceramics. (d) EDX mapping distribution of Ag, Pd, Ba, and Ti elements. Reproduced from ref (209). Copyright 2020 American Chemical Society.

These two simple precepts can be applied to most systems; e.g., a frequency dispersion of *ε*_r_ was observed after doping *x*Bi_2/3_(Mg_1/3_Nb_2/3_)O_3_ (B_2/3_MN) into BT ceramics, along with reduction on maximum dielectric constant (*ε*_m_) and associated temperature (*T*_m_).^209^ After fitting the *ε*_r_ and *T*_m_ using modified Curie–Weiss law (as follows)
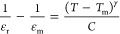
17γ was found to be within the range of 1.61 for BT–0.06B_2/3_MN ceramics with a Burns temperature ∼154 °C. In addition, XRD revealed a transformation from tetragonal to an average pseudocubic structure as increasing *x* concentration, coupled with a reduction in *P*_*r*_, confirming a relaxor state at room temperature, Figure 36a,b.

An optimum *W*_rec_ ∼ 4.55 J cm^–3^ at 520 kV cm^–1^ was recorded for BT–0.06B_2/3_MN (Figure 36)^209^ with similar properties reported for Bi(Mg_0.5_Ti_0.5_)O_3_,^208^ Bi(Li_0.5_Ta_0.5_)O_3_,^203^ Bi(Zn_0.5_Zr_0.5_)O_3_,^199^ and K_0.73_Bi_0.09_NbO_3_^499^ doped BT ceramics. However, the advantage of BT–B_2/3_MN is in the comparatively low concentration of dopant required to induce a relaxor state. The polar coupling is disrupted through a combination of A-site (Bi and *V*_*O*_^..^) and B-site (Nband Mg) dopants which provide a range of difference in ionic size, charge and electronegativity. However, only 4 mol % Bi is present on the A-site which minimizes reaction with Ag/Pd (Figure 36c). MLs with Ag–Pd internal electrodes may then be fired in ambient *p*O_2_, minimizing *V*_*O*_^..^ and reducing leakage current at high fields.

**Figure 37:**
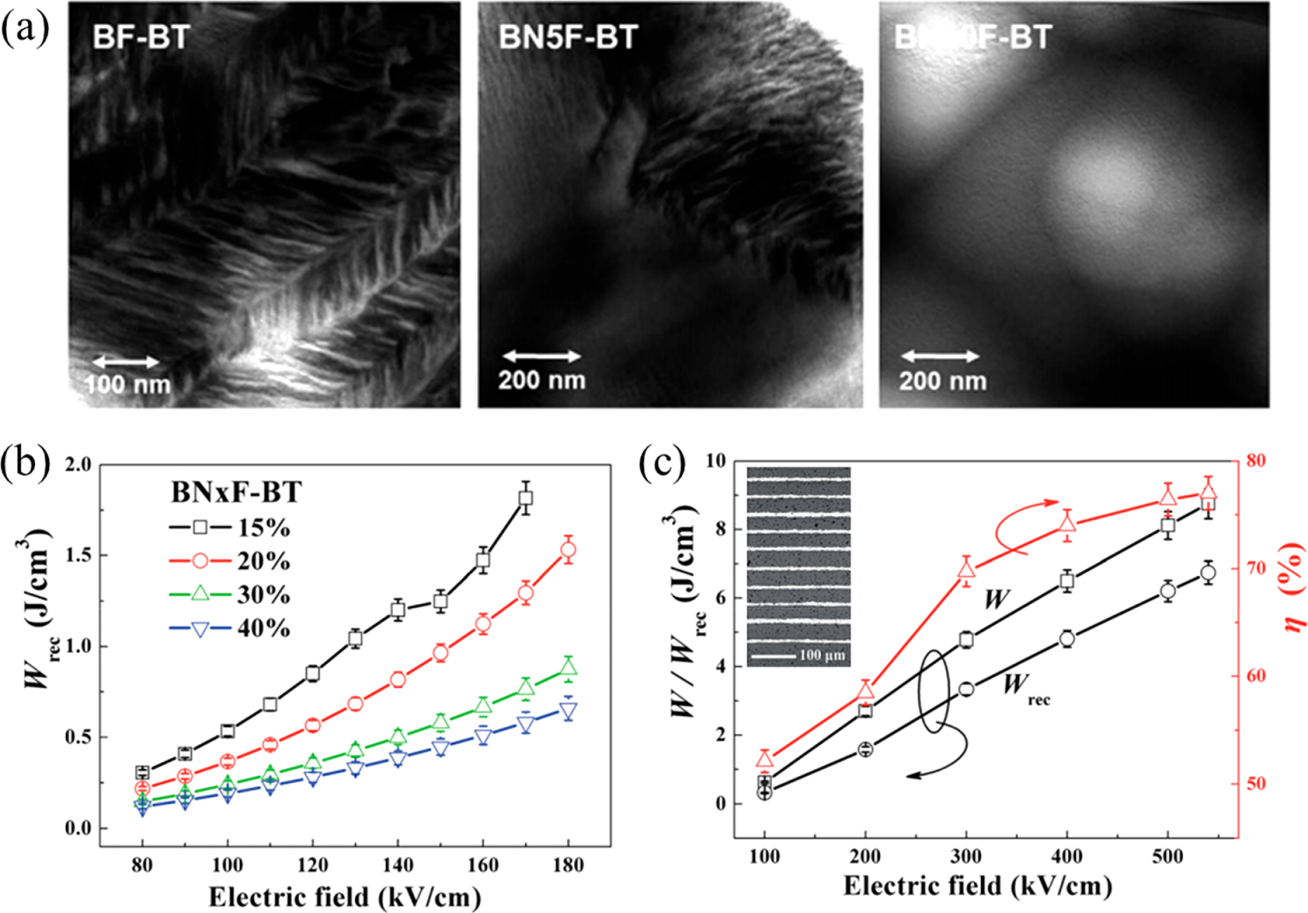
(a) TEM images of the domain structure in BF–BT, 5 mol % Nd–BT–BT and 10 mol % Nd–BF–BT. (b) The changes of *W*_rec_ as a function of electric field for x mol % Nd–BF–BT ceramics. (d) *W*, *W*_rec_, and η for 15 mol % Nd–BF–BT MLs, with ceramic MLs microstructure as inset figure. Reproduced with permission from ref (90). Copyright 2018 Royal Society of Chemistry.

Similar compositional modifications to induce a relaxor state have been adopted in BF-based solid solution. For example, in FE BF–BT compositions, macroscopic herringbone-type domains are observed in (Figure 37a) but doping with 5 mol % Nd, induced a nanodomain state, accompanied by a decrease in *P*_*r*_ and a frequency-dependent permittivity curve, confirming the transformation from FE to RFE. Simultaneously, average *G* was reduced from ∼10 μm (BF–BT) to ∼2 μm (Nd doped BF–BT). As a result, enhanced *W*_rec_ ∼ 1.8 J cm^–3^ was obtained for 15 mol % Nd doped BF–BT, which was further improved to 6.74 J cm^–3^ by multilayering (Figure 37b,c)^90^ with similar optimization reported in BF doped with Bi(Zn_2/3_Nb_1/3_)O_3_ and Bi(Zr_0.5_Zn_0.5_)O_3_.^34,266^ The same design strategy of inducing an RFE state is also used in thin film BF–BT–ST compositions.^452^

**Figure 38:**
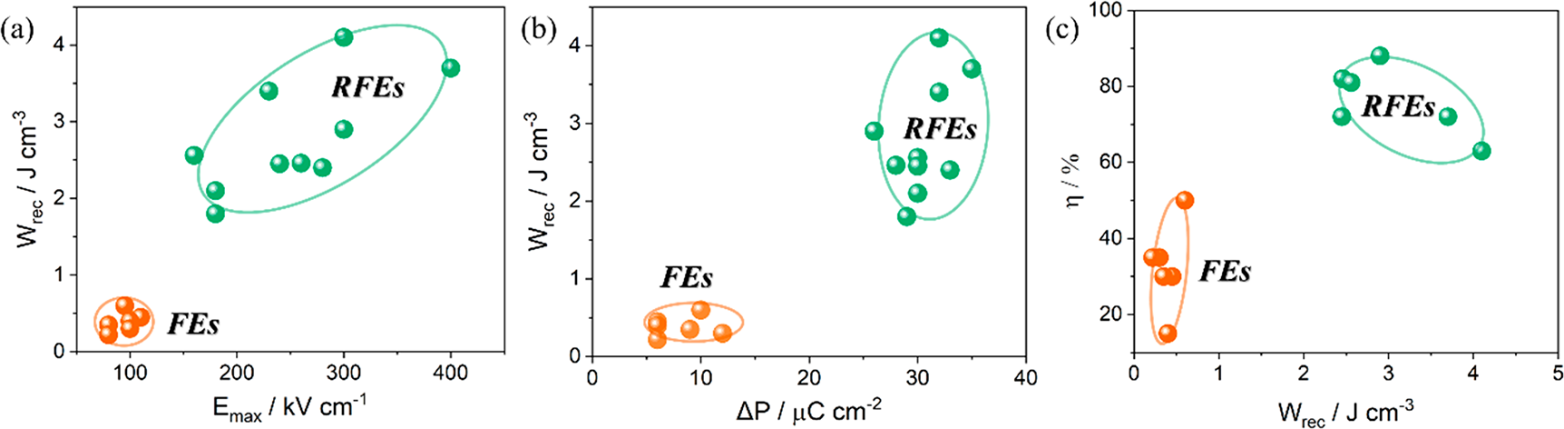
(a) *E*_max_ vs *W*_rec_, (b) Δ*P* vs *W*_rec_ and (c) *W*_rec_ vs η for FEs and RFEs bulk ceramics to demonstrate the relaxor optimization.

In summary, the inferior energy storage performance of all FEs can be improved by forcing a RFE state through strategic doping or alloying, Figure 38.^34,66,90,92,199,204,272^*P*_max_ is often unsaturated in RFE and increases with *E* which means *ΔP* is not only a function of the slimness of the *P–E* loop but also of the applied *E*. RFEs are therefore, among the most promising candidates for capacitors in power electronics (*E*_max_ > 300 kV cm^–1^).

### 4.2 Optimization of Antiferroelectrics

**Figure 39:**
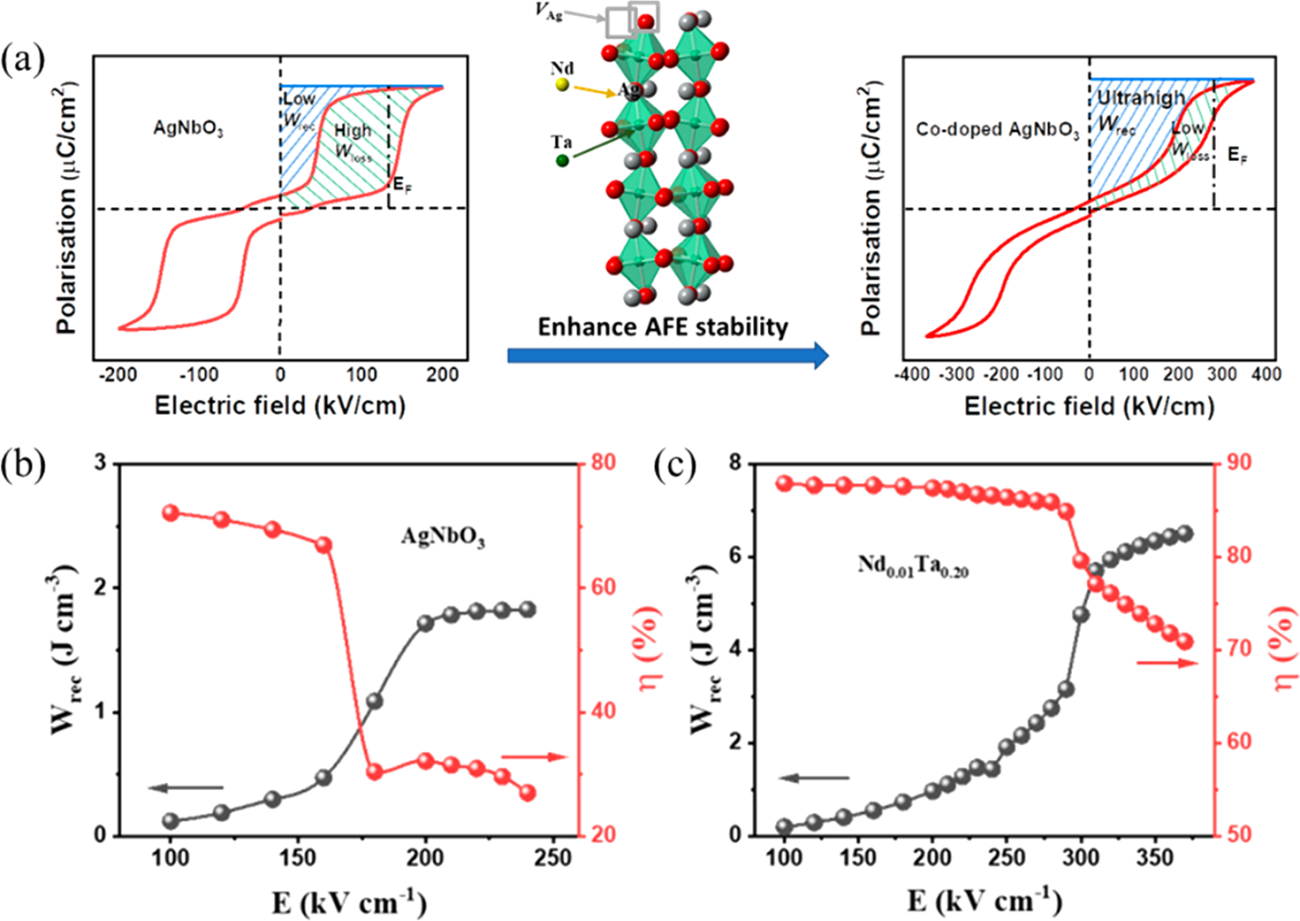
(a) Schematic illustrating how *W*_rec_ is optimized through doping in AN. Gray, yellow, green, dark green, and red spheres represent Ag, Nd, Nb, Ta, and O atoms, respectively. The *W*_rec_ and η of (b) AN and (c) Nd_0.01_Ta_0.20_ codoped AN under the respective electric fields.^366^

**Figure 40:**
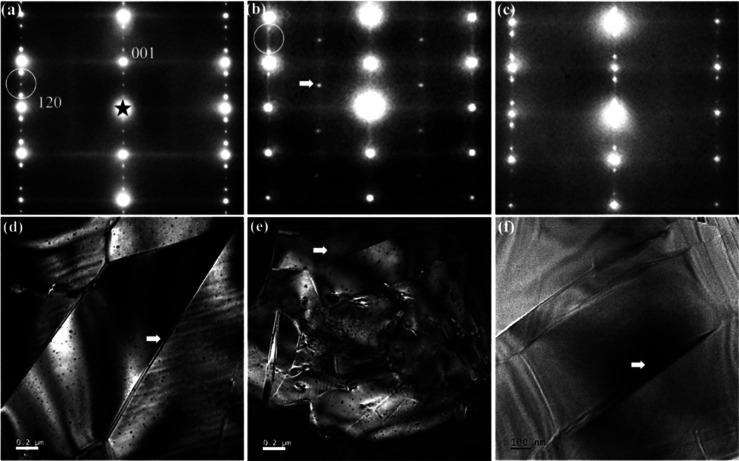
TEM [210]_c_ (c = cubic) zone axis diffraction patterns and corresponding dark field images obtained using (001) reflections from (a) and (b) AN and (c) and (d) Ag_0.91_Nd_0.03_NbO_3_ (e) [210]_c_ zone axis diffraction pattern of Ag_0.97_Nd_0.01_Ta_0.20_Nb_0.80_O_3_. (f) Bright field TEM image of domains in a grain of Ag_0.97_Nd_0.01_Ta_0.20_Nb_0.80_O_3_.^366^

Many recent publications have focused on optimization of energy storage in lead-free AN-based materials,^347,353^ although similar strategies date back to early studies of lead-based PLZT and PLZST AFE ceramics.^118,126,143−145^ The most comprehensive study of AN AFE ceramics was performed by Lu and co-workers in which they used A and B-site substitutions to develop, Ag_0.97_Nd_0.01_Nb_0.80_Ta_0.20_O_3_ which yielded *W*_rec_ ∼ 6.5 J cm^–3^ at 370 kV cm^–1^ with η∼ 71%, Figure 39.^366^ In their study, Lu and co-workers defined several key points required to optimize AN-based ceramics:

i) Optimization of *P*_max_ through local strain/field coupling around the smaller (with respect to Ag) Nd ion the A-site and its compensating V_A_, Figure 39a.
ii) Stabilization of the AFE structure through a combination of Nd and Ta doping which leads the induced AFE/FE transition to higher fields. Figure 39(a).
iii) Inducing a slim hysteresis curve in the field induced region of the *P–E* loop. This was also achieved, through Nd doping that disrupted polar and antipolar coupling which manifested itself as a decrease in domain width from ∼1 to 0.5 μm and streaking of ±1/4(001)_c_ superstructure reflections in electron diffraction patterns for *x* = 0.03, Figure 40.

All the above maximize the area of the *P–E* loop to the left of the curve in the positive quadrant and thus optimize *W*_rec_, Figure 39b,c.

**Figure 41:**
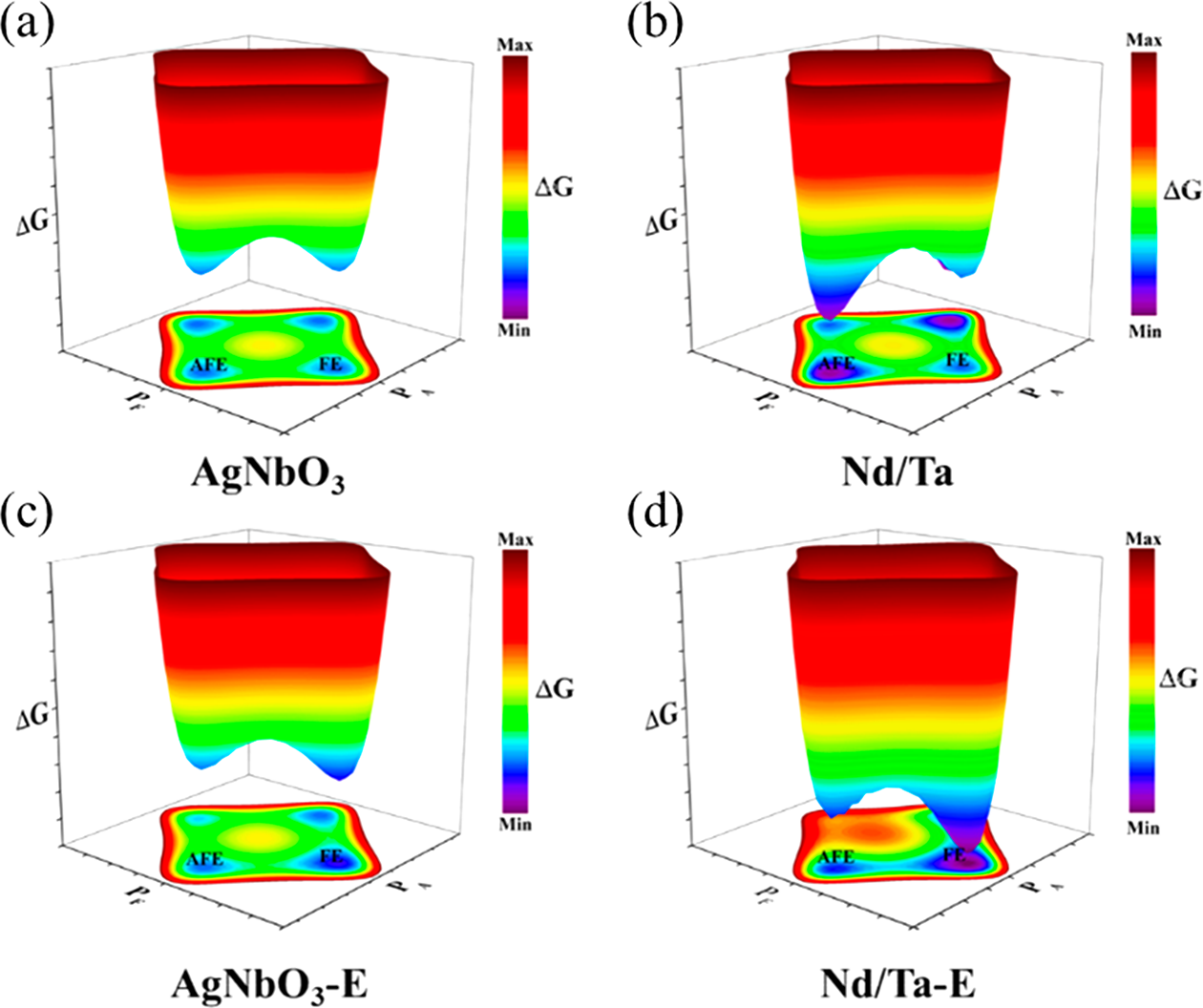
Schematic contour diagrams of the free energy difference (Δ*G*) for (a) AN- and (b) Nd/Ta-codoped AN without electric field. Schematic contour diagrams of GLD phenomenological theory of AFE-to-FIE phase transition for (c) AN- and (d) Nd/Ta-codoped AN under application of electric field.^366^

Stabilization of the AFE structure was also confirmed by First-principles calculation and Ginzburg–Landau–Devonshire (GLD) phenomenology, as illustrated in Figure 41.

**Figure 42:**
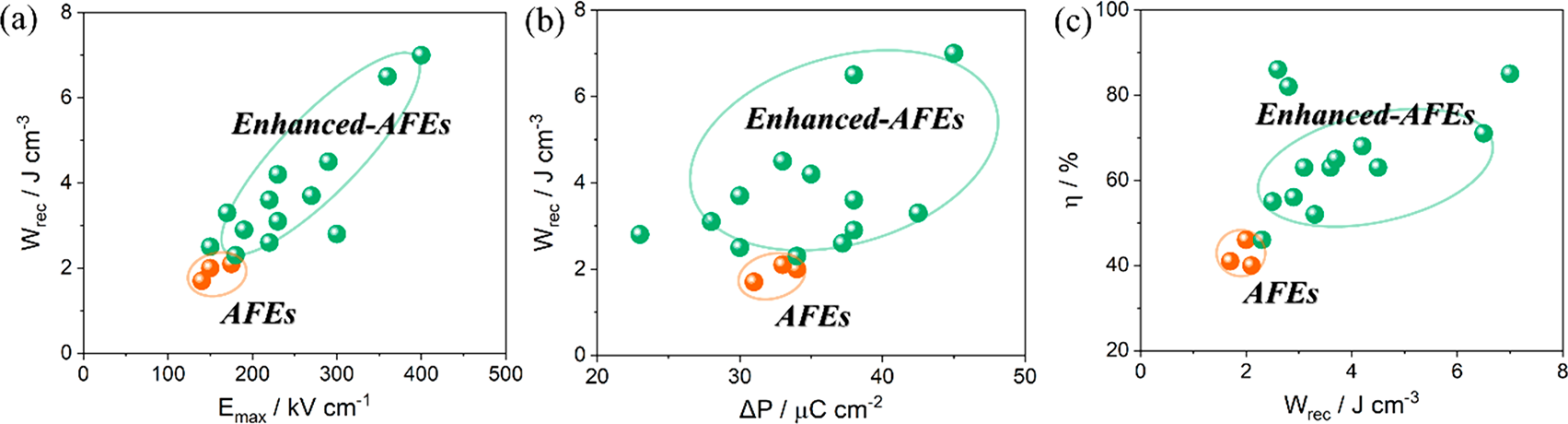
(a) *E*_max_ vs *W*_rec_ and (b) Δ*P* vs *W*_rec_ and (c) *W*_rec_ vs η for AFEs and stabilized AFEs bulk ceramics to demonstrate the AFE stabilized optimization.

Figure 42 summarizes the properties for many AFE systems. Most conventional AFEs exhibit low *W*_rec_ (∼1.5–2 J cm^–3^) and η (∼40%), which can be optimized to *W*_rec_ > 3 J cm^–3^ and η > 50% (enhanced-AFEs) by strategies described in the work of Lu and others.^348,350−352,357,359,363,366^ Similar values of *ΔP* (30–40 μC cm^–2^) are found for AFEs and enhanced-AFEs which reflects an intrinsic limitation of AFE materials, i.e. when antiparallel polar coupling is fully switched to polar under electric field, *P*_max_ reaches saturation and is difficult to enhance unlike for RFEs (section 4.1). η is also difficult to further improve (>80%) due to the hysteresis above the AFE/FE switching field. However, at intermediate electric fields (∼300 kV cm^–1^), much higher *ΔP* and *W*_rec_ are obtained for AFEs compared with RFEs, indicating that AFEs are more suitable for low/intermediate-voltage energy storage applications.

### 4.3 Other Strategies

#### 4.3.1 Chemical Coating

**Figure 43:**
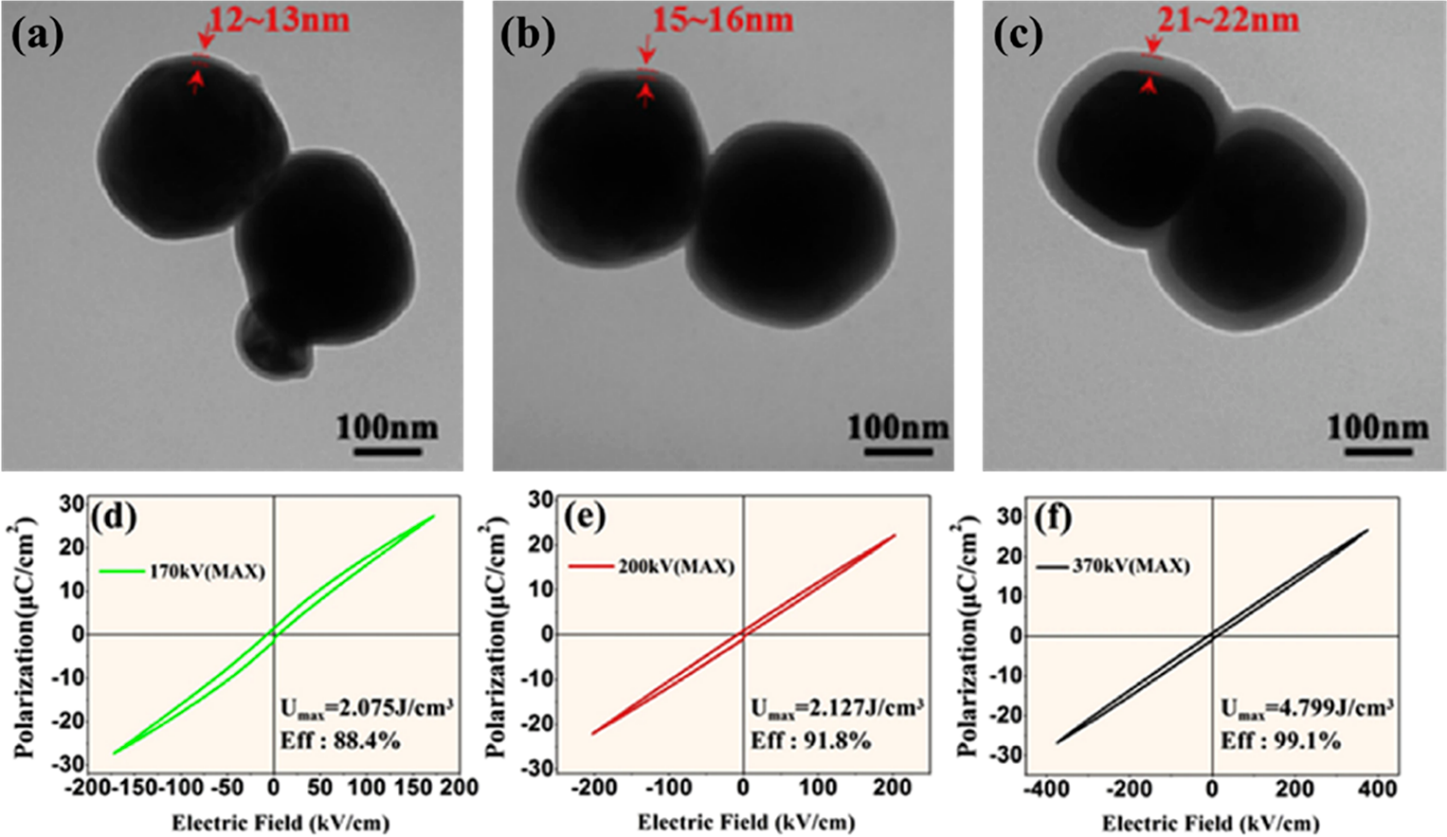
(a–c) TEM micrographs of BTnanoparticles coated with SiO_2_: (a) BT@10 wt %SiO_2_; (b) BT@15 wt %SiO_2_; (c) BT@20 wt %SiO_2_. The shell region is defined by red arrows and dash lines. Bipolar *P–E* loops of BT with (d) 10 wt % (e) 15 wt % (f) 20 wt % SiO_2_ composite ceramics at the highest applied electric field, measured at 10 Hz and room temperature. Reproduced with permission from ref (88). Copyright 2019 Elsevier.

Chemical coating is commonly reported as a strategy to optimize energy storage properties in BT ceramics. In general, chemical coatings are synthesized and applied using wet-chemical and sol–gel methods.^69,74,79−81^ Smaller grain sizes are typically obtained (on the order of a few nm), leading to the enhanced density and BDS. BT ceramics, for example, with a 4 nm thick SiO_2_ coating, have a good value of *W*_rec_ (∼1.43 J cm^–3^),^233^ in which SiO_2_ coating inhibits grain growth, thereby modifying the microstructure and reducing DC current leakage. The effect of SiO_2_ layer thickness on BT particles has been systematically investigated (Figure 43a-c). The highest *W*_rec_(∼4.8 J cm^–3^) and η (∼99.1%) were obtained for 20 wt % SiO_2_ coated BT, as shown in Figure 43d–f.^88^ Other coating materials such as Al_2_O_3_ and La_2_O_3_,^80,184^ are also reported to optimize energy storage properties, as listed in Table 14.

**Table 14:** Energy Storage Performance of Chemically Coated Materials

materials	coating/thickness	*E*_max_ (kV cm^–1^)	W_rec_ (J cm^–3^)	η (%)	*G* (nm)	ref
BT	Al_2_O_3_/2 nm	108	0.51	80	∼160	(80)
BT	La_2_O_3_ SiO_2_/20 nm	136	0.54	85	∼250	(184)
BT	BiScO_3_/ 4 nm	120	0.68	81	∼100	(500)
BT	SiO_2_ and Al_2_O_3_/6 nm	190	0.725	80	∼80	(73)
BT	SiO_2_/10 nm	200	1.2	53.8	∼200	(501)
BT	SiO_2_/4 nm	290	1.43	53	∼120	(86)
Pb_0.97_La_0.02_(Zr_0.33_Sn_0.55_Ti_0.12_)O_3_	SiO_2_/2 nm	238	2.68	95	∼180	(87)
BT	SiO_2_/21 nm	370	4.799	95	∼220	(88)
Pb_0.91_La_0.06_(Zr_0.552_Sn_0.368_Ti_0.08_)O_3_	PbO-B_2_O_3_–SiO_2_–Al_2_O_3_–ZnO-MnO_2_/4 nm	402	7.4		∼250	(77)

#### 4.3.2 Layered Structure

**Figure 44:**
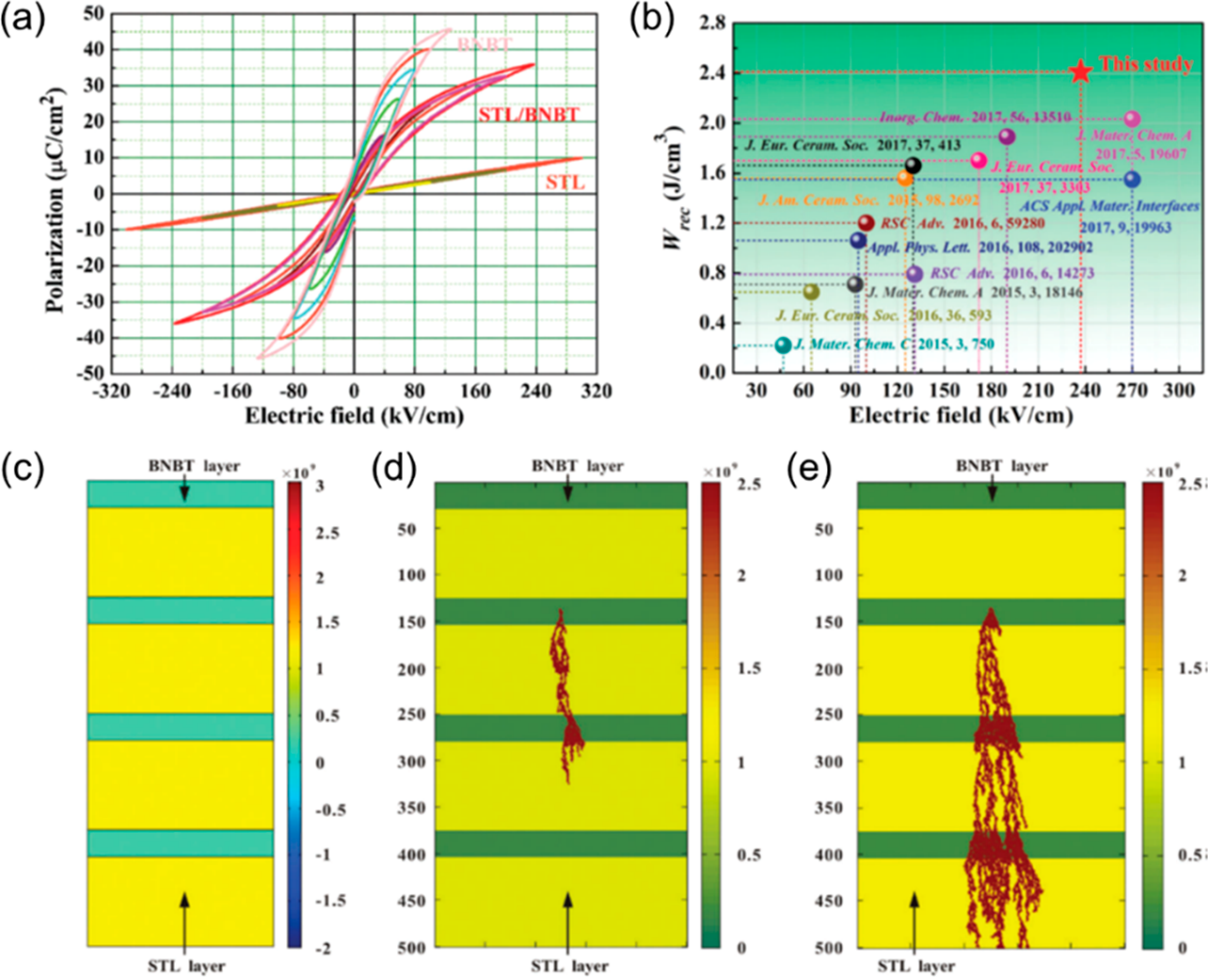
(a) Bipolar *P–E* loops of the ST+Li_2_O_3_, NBT–0.06BT and ST + Li_2_O_3_/NBT–0.06BT ceramic MLs. (b) Comparison of *W*_rec_ and electric field between the ST + Li_2_O_3_/NBT–0.06BT ceramic MLs and some recently reported lead-free ceramics. (c) Distribution of the electric field at 200 kV cm^–1^, model of electrical tree propagation simulated using the finite element method for the ST + Li_2_O_3_/NBT–0.06BT MLs ceramic under (d) 200 kV cm^–1^ and (e) 250 kV cm^–1^. Reproduced with permission from ref (442). Copyright 2018 Royal Society of Chemistry.

Layer-structures composed of multiple materials have been reported to optimize energy storage properties and are typically tape cast, followed by lamination. Both BDS and ε_r_ are optimized with the final properties related to the type of electroceramic material and the thickness of each layer. The BDS of BT-based ceramics was enhanced to >300 kV cm^–1^ by laminating layers between BT–*x* wt % SiO_2_ layers (*t* ∼ 20 μm) and BT layer (*t* ∼ 25 μm).^441^ ε_r_ decreased but this was compensated by an increased BDS with increasing SiO_2_ concentration in the BT–*x* wt % SiO_2_ layers. ST + Li_2_CO_3_ (*t* ∼ 50 μm) and 0.93NBT–0.07Ba_0.94_La_0.04_Zr_0.02_Ti_0.98_O_3_ (*t* ∼ 33 μm) layered structure were also fabricated via tape-casting with improved *W*_rec_ ∼ 2.72 J cm^–3^ at 294 kV cm^–1^.^502^ Enhanced *W*_rec_ ∼ 2.41 J cm^–3^ at 237 kV cm^–3^ was also obtained for layer-structure ceramics with ST + Li_2_O_3_ (*t* ∼ 50 μm) and NBT–0.06BT (*t* ∼ 50 μm), Figure 44a.b.^442^ The interface between the ST + Li_2_O_3_ and the NBT–0.06BT layer was further investigated using the finite element analysis. BDS was improved by reducing the breakdown paths between the ST + Li_2_O_3_ and the NBT–0.06BT layer, Figure 44c–e^442^ with electrical field redistribution and interface blocking playing essential roles.^503^ BDS was also influenced by the difference in ε_r_ and thickness ratio between the adjacent layers.

## 5 Summary and Perspectives

### 5.1 Lead-Based Energy Storage Ceramics

Lead-based ceramics have great potential as energy storage materials in modern microelectronics where high voltage and temperature are required, such as in pulsed power and power electronic applications. Lead-based AFE-type ceramics exhibit extremely high energy density but optimizing BDS, η and minimizing electrostrain is problematic. Low BDS (<300 kV cm^–1^) is often attributed to the volatilisation of lead/lead oxide which leads the formation of lead vacancy (*V*_*pb*_^..^) and *V*_*O*_^..^ that results in current leakage. Such issues may be partially solved by a combination of improved processing and dopants but achieving the values of BDS observed in lead-free materials has proved elusive. The low η in lead-based AFE-type ceramics (<80%) is mainly a result of opening of the hysteresis loop at high field due to the stabilization of a field induced FE phase. This results in a change in crystal class from tetragonal (AFE_T_) to rhombohedral (FE_R_) giving large strains (>0.3%) which may prevent long-term cycling through mechanical fatigue.

The lack of popularity in researching lead-based compared with lead-free materials in the academic community has meant that exploration of novel systems is rather limited, but there are, for example, interesting mixed Pb- and Bi-based systems with high ε_r_ and a spontaneous polarization that would mirror some of the design principles adopted in lead-free ceramics, particularly in solid solutions which combine AFEs and relaxor end members. In addition, further work is required to understand crystal structure and phase transition behavior. Many systems have incommensurate modulations and their influence on AFE/FE switching needs to be explored further using *in situ* XRD and Raman (temperature/electric field), as well as utilizing advanced aberration corrected TEM to study the local structure.

### 5.2 Lead-Free Energy Storage Ceramics

Lead-free candidates, including BT, ST, BF, KNN, NBT, AN and NN-based systems, are extensively studied and summarized in this review. Research into lead-free materials far outweighs that in lead-based, due to how the potential environmental legislation surrounding manufacturing and the end use of lead-based products has influenced funding bodies and awards. As a result, the optimization of energy storage properties has progressed rapidly in the last 5 years. Successful strategies to improve properties include, disrupting long-range polar coupling particularly if the average ionic polarizability is increased or unaffected, construction relaxor feature (PNRs) in FEs and AFEs, enhancing *E*_g_ and as a consequence *E*_a_, reducing the total electrical conductivity and promoting electrical homogeneity through the use of strategic dopants to modify defect chemistry. If these strategies are married with a reduction in the dielectric layer thickness, high values of *W*_rec_ ∼ 20 J cm^–3^ and η ∼ 90% can be achieved. Recent work on texturing of ceramic MLs has also proved successful in enhancing *W*_rec_ but the complexity of this approach may inhibit commercial uptake. However, the two overriding issues with the majority of lead-free compositions, particularly those whose *W*_rec_ are >10 J cm^–3^ are (i) the need to find an effective low cost internal electrode system that permits their commercial exploitation (currently almost all ML data is quoted with Pt internal electrodes) and (ii) pushing their operating window to >200 °C and >100 Hz. Interestingly, electrostrain, a major drawback in lead-based materials, is broadly speaking not an issue in most of the lead-free RFEs and AFEs since the measured values of strain are often significantly lower (<0.2%) even at high fields. As with lead containing ceramics, there are only a few comprehensive investigations of the energy storage mechanisms which require high field *in situ* studies to be performed. A greater understanding of the role of defect chemistry, doping and alloying is also required, particular on how this influences, *E*_g_, resistivity and electrical homogeneity, and thus the *W*_rec_ and η, thermal stability and cyclic reliability. In addition, in commercial MLCCs, the ripple current, equivalent series *R*, failure mode, voltage rating, the reliability in high humidity need to be evaluated and explored.

### 5.3 Glass Ceramics

Glass-ceramics have the advantages of facile manufacture, high *W*_rec_, ultrahigh η (low energy dissipation), ultrafast charge–discharge speed, excellent temperature/frequency stability. However, there are still challenges/problems. Generally, increasing the volume fraction of the crystal phase will increase the ε/*P* but decrease BDS. It is critical to balance the *ε*_r_ and BDS to obtain the highest *W*_rec_. The mechanism of crystallization and control of crystal phase/microstructure is still ambiguous, which should be further investigated using, advanced TEM and *in situ* XRD/TEM as a function of applied field and temperature. Furthermore, although the theoretical *W*_rec_ (>15 J cm^–3^, due to ultrahigh BDS, >1100 kV cm^–1^) of glass-ceramics are much higher than other bulk ceramics and even MLs (15–20 J cm^–3^), the measured/calculated *W*_rec_ by *P–E* loops and discharging processes is low (<2 J cm^–3^) due to the lower applied electric fields. As a result, we recommend using the same test method (*P–E* loops and discharging process) to evaluate the practical energy storage performance for glass-ceramics, consistent with other dielectrics.
